# 4,5-Disubstituted 1,2,3-triazoles: Effective Inhibition of Indoleamine 2,3-Dioxygenase 1 Enzyme Regulates T cell Activity and Mitigates Tumor Growth

**DOI:** 10.1038/s41598-019-54963-9

**Published:** 2019-12-05

**Authors:** Subhankar Panda, Nirmalya Pradhan, Soumya Chatterjee, Sudhir Morla, Abhishek Saha, Ashalata Roy, Sachin Kumar, Arindam Bhattacharyya, Debasis Manna

**Affiliations:** 1 Department of Chemistry, Indian Institute of Technology Guwahati, Guwahati, 781039 Assam India; 2 Department of Zoology, University of Calcutta, Kolkata, 700019 West Bengal India; 3 Department of Bioscience and Bioengineering, Indian Institute of Technology Guwahati, Guwahati, 781039 Assam India

**Keywords:** Drug discovery and development, Oxidoreductases

## Abstract

The improvement of body’s own immune system is considered one of the safest approaches to fight against cancer and several other diseases. Excessive catabolism of the essential amino acid, L-tryptophan (L-Trp) assists the cancer cells to escape normal immune obliteration. The formation of disproportionate kynurenine and other downstream metabolites suppress the T cell functions. Blocking of this immunosuppressive mechanism is considered as a promising approach against cancer, neurological disorders, autoimmunity, and other immune-mediated diseases. Overexpression of indoleamine 2,3-dioxygenase 1 (IDO1) enzyme is directly related to the induction of immunosuppressive mechanisms and represents an important therapeutic target. Several classes of small molecule-based IDO1 inhibitors have been already reported, but only few compounds are currently being evaluated in various stages of clinical trials as adjuvants or in combination with chemo- and radiotherapies. In the quest for novel structural class(s) of IDO1 inhibitors, we developed a series of 4,5-disubstituted 1,2,3-triazole derivatives. The optimization of 4,5-disubstituted 1,2,3-triazole scaffold and comprehensive biochemical and biophysical studies led to the identification of compounds, **3i**, **4i**, and **4k** as potent and selective inhibitors of IDO1 enzyme with IC_50_ values at a low nanomolar level. These potent compounds also showed strong IDO1 inhibitory activities in MDA-MB-231 cells with no/negligible level of cytotoxicity. The T cell activity studies revealed that controlled regulation of IDO1 enzyme activity in the presence of these potent compounds could induce immune response against breast cancer cells. The compounds also showed excellent *in vivo* antitumor efficacy (of tumor growth inhibition = 79–96%) in the female Swiss albino mice. As a consequence, this study describes the first example of 4,5-disubstituted 1,2,3-triazole based IDO1 inhibitors with potential applications for immunotherapeutic studies.

## Introduction

Immunotherapy is currently considered as one of the most remarkable and promising advances in cancer research. Recent achievements with the immune checkpoint inhibitors such as anti-PD-1, anti-CTLA4, and anti-PD-L1 monoclonal antibodies have demonstrated impressive therapeutic effects in multiple clinical trials against a wide range of cancers^1–5^. Until recently, only a few patients responded positively to this cancer immunotherapy. Hence, it is essential to pinpoint alternate immunosuppressive mechanisms in cancer. The catabolism of essential amino acid L-tryptophan (L-Trp) via the kynurenine pathway produces metabolites like kynurenine, 3-hydroxy kynurenine, kynurenic acid, excitotoxin quinolinic acid, and others that primarily regulate local immune responses. In nonhepatic cells, heme-containing enzyme indoleamine 2,3-dioxygenase 1 (IDO1) is known to catalyze the oxidative cleavage of the C2–C3 indole double bond of the L-Trp to produce *N*-formylkynurenine. The IDO1 expression is usually low in healthy humans and has little physiological effects. Whereas, under pathophysiological conditions (e.g., in tumor cells) inflammatory signals up-regulate the expression of IDO1 enzyme^5–8^. Inflammatory cytokines like interferon-γ (IFN-γ), tumor necrosis factor-α (TNF-α), bacterial lipopolysaccharides and others are also usually accountable for the over-expression of IDO1 enzyme within the immunosystem. Cytotoxic T lymphocytes are known to produce this interferon-γ, which possibly plunders the efficiency of other immune therapeutic approaches against cancer^4,5,8^.

In tumor-draining lymph nodes, the IDO1 enzyme is expressed within antigen-presenting cells (APCs) and augments peripheral tolerance to tumor-associated antigens (TAAs). Up-regulation of IDO1 enzyme alters local immune response by generating surplus kynurenine-pathway metabolites that act as natural ligands for the aryl hydrocarbon receptor (AhR) and reduce the local L-Trp concentration to trigger general control nonderepressible 2 (GCN2) kinase activation^5,9,10^. Thus, IDO1 mediated L-Trp metabolism produces a series of immunosuppressive metabolites that suppress T cell proliferation and also affect NK cell function that leads to immunosuppression^9^. For this reason, the IDO1 enzyme is known to perform as the sensor for tumor cells against T-cell attack. The overexpression of IDO1 enzyme is also correlated with poor prognosis in different types of cancers, including colorectal, pancreatic, and ovarian^6,7^. Various preclinical studies with cancer models suggest that overexpression of IDO1 enzyme induces tumor progression and metastasis. Numerous studies have also shown that inhibition of IDO1 enzyme activity improves the effectiveness of chemotherapeutic and radio-therapeutic treatment of malignant tumors^9,11–14^. Up-regulated IDO1 activity is also associated with the neurodegenerative disorder, HIV-1 encephalitis, and other diseases that are related to the pathological immune suppression^8,15–17^. These research findings highlight the efficiency of IDO1 in cancer immunotherapy and other treatments.

Several small molecule-based IDO1 inhibitors in combination with immune checkpoint inhibitors are currently being evaluated in various stages of clinical trials for the treatment of different types of cancers and other diseases^8^. However, several other potent IDO1 inhibitors with diverse structural classes, including *N*-hydroxyamidine, imidathiazole, tryptophan, triazole, imidazole, and others. have been reported^5,8^. Successful use of immune checkpoint inhibitors and recent clinical developments of the inhibitors of the IDO1 enzyme instigate scientists to develop potent small molecule-based IDO1 inhibitors that will adequately address this cancer immunotherapeutic approach.

In an endeavor to identify effective IDO1 inhibitor(s), we designed and synthesized a series of 4,5-disubstituted 1,2,3-triazole based compounds. Activity studies revealed that few 1,2,3-triazoles showed potent IDO1 inhibitory efficacies (IC_50_ values in the nanomolar range). These selected 1,2,3-triazoles also showed low nanomolar IDO1 inhibitory activity in MDA-MB-231 cells with very low cytotoxicity. Additional studies also revealed that these potent compounds have a stronger binding affinity and higher selectivity for the IDO1 enzyme in comparison with the tryptophan 2,3-dioxygenase (TDO) enzyme. These potent compounds up-regulate the proliferation and activity of cytotoxic CD8 + T cells. The *in vivo* studies showed that these selected compounds have excellent antitumor activity with tumor growth inhibition (TGI) = 79–96% in the female Swiss albino mice. The *in vitro* and *in vivo* efficacies of these compounds make the 4,5-disubstituted 1,2,3-triazole scaffold of overwhelming importance for further development of therapeutic agents targeting IDO1 enzyme and others.

## Result and Discussion

### Design and synthesis of 4,5-disubstituted 1,2,3-triazoles

Identification of potent IDO1 inhibitors based on a 4,5-disubstituted 1,2,3-triazole scaffold is of interest, as the triazoles have been used as an alternative to the imidazole scaffold for its efficacy in providing better specificity for IDO1 over other heme-containing proteins. Rationally designed 1,2,3-triazole derivative 4-chloro-2-(1*H*-1,2,3-triazol-5-yl)phenol (MMG-0358) showed nanomolar-level IDO1 inhibitory activities both in enzymatic and cellular assays^18^. The MMG-0358 also showed low cytotoxicity and higher selectivity for IDO1 over TDO enzyme^18,19^. The *N*-(2-Ethylphenyl)-1*H*-1,2,3-triazol-5-amine also showed nanomolar-level cellular IDO1 inhibitory activities^8^. Such higher potency of the 1,2,3-triazol derivatives instigates us to design, synthesize, and explore the efficacy and consequences of 4,5-disubstituted 1,2,3-triazole derivatives in IDO1 inhibition^8^. We hypothesize that the 4,5-disubstituted 1,2,3-triazole could provide higher inhibitory potency and specificity for the IDO1 enzyme because of its probable interaction with both heme-group and Ser-167, a crucial residue in substrate/inhibitor binding to the active site of IDO1 enzyme. Besides, apposite occupation of the active site by the 4,5-disubstituted 1,2,3-triazole derivatives could be advantageous for their higher selectivity and better inhibitory activities for the IDO1 enzyme. Whereas, MMG-0358 and other triazole based compounds, which partially occupy the active-site might lack the selectivity and inhibitory activities for the IDO1 enzyme in comparison with the other heme-containing proteins.

**Figure 1:**
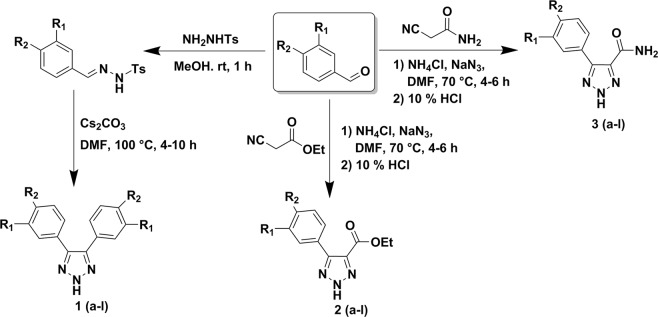
Synthesis of 4,5-diaryl 2*H*-1,2,3-triazole derivatives (**1a–l**), 4-carboxylate-5-aryl-2*H*-1,2,3-triazole derivatives (**2a–l**) and 4-carboxamide-5-aryl-2*H*-1,2,3-triazole derivatives (**3a–l**).

**Figure 2:**
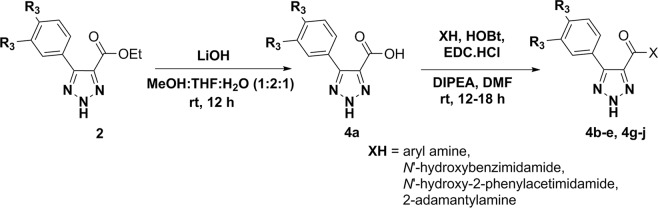
Synthesis of 4-carboxamide-5-aryl-2*H*-1,2,3-triazole derivatives (**4a–e,g–j**).

**Figure 3:**
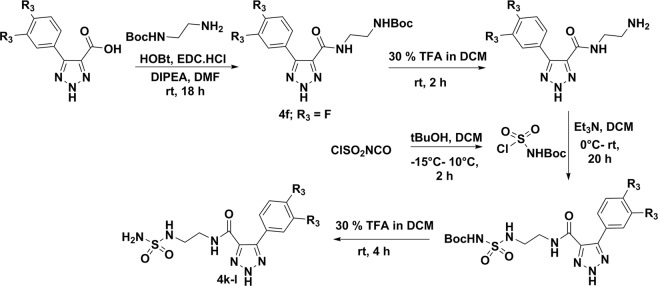
Synthesis of 4-carboxamide-5-aryl-2*H*-1,2,3-triazole derivatives (**4f,k,l**).

In this report, we described the synthesis of substituted 4,5-diaryl 2*H*-1,2,3- triazoles from the corresponding *N*-tosyl aryl hydrazone(s) under transition metal, azide, and oxidant-free conditions using a recently developed reaction method by our group (Fig. 1)^20^. The use of Cs_2_CO_3_ as the base and DMF as the solvent allowed the formation of these 2*H*-triazoles under ambient temperature (Fig. 1)^20^. We also synthesized 4-carboxylate-5-aryl-2*H*-1,2,3-triazoles and 4-carboxamide-5-aryl-2*H*-1,2,3-triazoles according to the reported procedures^21,22^. Condensation of cyanoethyl acetate or 2-cyanoacetamide with aromatic aldehyde in the presence of sodium azide and ammonium chloride allowed the formation of 4-carboxylate-5-aryl-2*H*-1,2,3-triazoles and 4-carboxamide-5-aryl-2*H*-1,2,3-triazoles, respectively with moderate to excellent yield (Fig. 1)^21,22^. Additional *N*- modifications of the 4-carboxamide moiety were synthesized according to the reported procedures^23^. Hydrolysis of 4-carboxylate-5-aryl-2*H*-1,2,3-triazoles in the presence of LiOH in MeOH:THF:H_2_O (1:2:1) solution resulted 5-aryl-2*H*-1,2,3-triazole-4-carboxylic acid^23^. Coupling of 5-aryl-2*H*-1,2,3-triazole-4-carboxylic acid also carried out with arylamine, *N’*- hydroxybenzimidamide, *N*’- hydroxy-2- phenylacetimidamide, 2-adamantylamine or tert-butyl (2-aminoethyl)carbamate in the presence of HOBt and EDC.HCl in DMF solvent resulting the formation of 4-carboxamide-5-aryl-2*H*-1,2,3-triazole derivatives (Figs. 2 and 3)^24^. Coupling of *N*-(2-aminoethyl)-5-aryl-2*H*-1,2,3- triazole-4-carboxamide with tert-butyl chlorosulfonylcarbamate, followed by removal of Boc- group resulted the target 5-aryl-*N*-(2-(sulfamoylamino)ethyl)-2*H*-1,2,3-triazole-4-carboxamide in moderate yields (Fig. 3)^25^. These short-step synthetic approaches are highly beneficial for rapid structural alterations and large-scale productions of 2*H*-triazoles.

### IDO1 Inhibitory activities of 4,5-disubstituted 1,2,3-triazoles

The IDO1 activities in the absence and presence of 4,5-disubstituted 1,2,3-triazole derivatives were initially evaluated using standard spectrophotometric method^8,19,26–30^. The absorption spectra of most of the compounds (10 nM to 100 μM) showed no or negligible interference with this spectrophotometric-based enzyme activity assay. The IC_50_ values of the reported compounds 4-amino-*N*-(3-chloro-4-fluorophenyl)-*N*´-hydroxy-1,2,5-oxadiazole-3-carboximidamide and MMG-0358 were 109 and 317 nM, respectively under the similar experimental conditions, which is in accordance with the reported IC_50_ values^18,31^. To improve the IDO1 inhibitory potency of the 4,5-disubstituted 1,2,3-triazole derivatives, we investigated two extensive modifications of the core-structure of the compounds: alteration of the 4,5-substitutions of the triazole ring and substitution of the aryl ring. In addition, we also investigated the effect of *N*-substituted 4-carboxamide derivatives of 1,2,3-triazoles on the IDO1 activity.

We first explored the role of 4,5-diaryl-substituted 1,2,3-triazole ring on IDO1 enzyme activity. The inhibitory activities of the lead compound **1a**, **2a**, and **3a** suggest that not only the presence of 1,2,3-triazole ring but also the nature and electronic properties of the substituents at the C4 and C5 positions of the triazole ring play a crucial role in their inhibitory efficiency against the purified hIDO1 enzyme. In comparison with 4,5-diphenyl 2*H*-1,2,3-triazole (**1a**, IC_50_ = 90.033 μM), the ethyl 5-phenyl-2*H*-1,2,3-triazole-4-carboxylate (**2a**, IC_50_ = 14.390 μM) or 5-phenyl-2*H*-1,2,3-triazole-4- carboxamide (**3a**, IC_50_ = 8.541 μM) showed 6- and 10-fold stronger IDO1 inhibitory activities, respectively (Table 1). To augment the efficiencies of these 1,2,3-triazoles, we then investigated the impact of halogen-substituted aryl ring on the IDO1 enzyme activity. We hypothesize that the presence of halogen-substituted aryl ring in the compound’s core structure could play a vital role in their IDO1 inhibitory efficiencies. The measured IC_50_ values suggested that appropriate halogen substitution(s) to the aryl ring of the 1,2,3-triazole moiety resulted in moderate to large change in inhibitory potency. For 4,5-diaryl containing 1,2,3-triazoles, the presence of 3-Br, 4-F-substituted diaryl moieties (**1 l**) showed 46-fold increase in inhibitory potency than the lead compound **1a**. Altogether, halogen derivatives of 4,5-diaryl substitutions showed reduced IDO1 inhibitory activity in comparison with that of mono-aryl substitution on the triazole ring. Among the tested halogen-substituted mono-aryl ring containing 1,2,3-triazoles, compound **2j**, **3i** and **3j** showed stronger IDO1 inhibition potency with IC_50_ values within the range of 70–200 nM (Table 1). This implies that the 3,4-dihalogen-substituted 5-aryl ring of 1,2,3-triazole scaffold might be adequate in maintaining the IDO1 inhibitory activity. Therefore, these data suggested that the presence of not only the halogen-substituted monoaryl ring but also carboxylate or carboxamide group to the triazole scaffold plays a vital role in displaying potent IDO1 inhibitory activity. The halogen-substituted aryl ring could be involved in interactions with the hydrophobic residues (Tyr126, Cys129, Val130, Phe163, Phe164, Ser167, Leu234, Gly262, Ser263, Ala264, and the heme ring) present within the ‘pocket-A’ of the IDO1 enzyme. In our previous reports, we successfully demonstrated that halogen substitution at the *meta-* and /or *para*-positions of the aryl ring augmented the IDO1 inhibitory efficiencies of *N*′-hydroxyamidines, fused-heterocycles^27,29^. Other research groups also confirmed such assistance of halogen-substituted aryl ring on the IDO1 inhibitory efficiencies^19,26,32^. Such stronger IDO1 inhibitory activities of the halogen-substituted monoaryl ring containing compounds could be because of the hydrophobic interactions, halogen bonding with the Lewis bases, and /or π-stacking interaction with aromatic amino acids present within the active site.

**Table 1:** Inhibitory activity of the 4,5-diaryl 2*H*-1,2,3-triazoles, 4-carboxylate-5-aryl-2*H*-1,2,3-triazoles and 4-carboxamide-5-aryl-2*H*-1,2,3-triazoles against purified human IDO1 enzyme.

Substituents		Compound 1		Compound 2		Compound 3	
R_1_	R_2_		hIDO1 IC_50_ (µM)^a^		hIDO1 IC_50_ (µM)^a^		hIDO1 IC_50_ (µM)^a^
H	H	**1a**	90.033 ± 2.942	**2a**	14.390 ± 3.357	**3a**	8.541 ± 0.084
H	Me	**1b**	54.871 ± 0.757	**2b**	1.699 ± 0.061	**3b**	7.082 ± 0.691
F	H	**1c**	4.991 ± 0.057	**2c**	31.440 ± 4.207	**3c**	6.611 ± 0.355
Cl	H	**1d**	2.513 ± 0.060	**2d**	2.550 ± 0.033	**3d**	2.687 ± 0.055
Br	H	**1e**	21.870 ± 1.270	**2e**	2.981 ± 0.012	**3e**	26.680 ± 2.199
H	F	**1f**	25.240 ± 1.703	**2f**	8.090 ± 0.210	**3f**	2.941 ± 0.034
H	Cl	**1g**	3.208 ± 0.100	**2g**	0.392 ± 0.084	**3g**	0.624 ± 0.023
H	Br	**1h**	40.720 ± 2.696	**2h**	18.450 ± 1.676	**3h**	5.156 ± 0.011
F	F	**1i**	7.721 ± 0.761	**2i**	0.545 ± 0.029	**3i**	0.111 ± 0.005
Cl	Cl	**1j**	12.910 ± 0.191	**2j**	0.163 ± 0.025	**3j**	0.069 ± 0.004
Cl	F	**1k**	6.913 ± 0.099	**2k**	1.691 ± 0.102	**3k**	1.831 ± 0.126
Br	F	**1 l**	1.935 ± 0.083	**2 l**	3.035 ± 0.091	**3 l**	1.993 ± 0.005

^a^hIDO1 IC_50_ values are the mean of three independent assays.

To augment the IDO1 inhibitory efficiencies of the 4,5-disubstituted 1,2,3-triazoles, we extended our activity studies with *N*-substituted 4-carboxamide-5-aryl-2*H*-1,2,3-triazoles. We hypothesize that the presence of *N*-substituted moiety in the core structural unit of the 1,2,3-triazoles could play a significant role in their IDO1 inhibitory efficiencies because of its interaction with the polar residues (Phe226, Arg231, Ser235, Phe291, Ile354, and Leu384) present within the ‘pocket B’, towards the entrance of the active site of the IDO1 enzyme. Structural modifications, including the installation of *N*′-hydroxyamidine (**4g–j**) and sulfamide (**4k** and **4l**) groups in the 4-carboxamide moiety of the compounds were also explored. Among the tested compounds **4i**, **4k** and **4l** showed high-potency in IDO1 inhibition with IC_50_ values in the range of 92–94 nM. Compound **4i**, **4k**, and **4l** showed around 71–86% and 82–93% of IDO1 inhibition at 4 and 100 μM concentrations, respectively (Table 2). However, other structural modifications of 4-carboxamide-5-aryl-2*H*-1,2,3-triazoles, including the installation of phenylamine (**4b–d**), adamantylamine (**4e**), or *N*′-Boc-ethylenediamine (**4f**) groups in the 4-carboxamide moiety of the 2*H*-triazole scaffold were ineffective against IDO1 activity.

**Table 2:** Inhibitory activity of the *N*-modified 4-carboxamide-5-aryl-2*H*-1,2,3-triazoles against purified human IDO1 enzyme.

Compound			hIDO1 IC_50_ (µM)^a^	Compound			hIDO1 IC_50_ (µM)^a^
	R_3_	X			R_3_	X	
4a	F	OH	17.420 ± 0.918	**4g**	F		0.880 ± 0.018
4b	Cl		19.400 ± 0.179	**4h**	F		3.809 ± 0.240
4c	Cl		2.007 ± 0.176	**4i**	F		0.094 ± 0.004
4d	F		0.271 ± 0.009	**4j**	F		2.357 ± 0.308
4e	Cl		18.650 ± 0.398	**4k**	F		0.092 ± 0.012
4f	F		2.745 ± 0.195	**4l**	Cl		0.093 ± 0.009

^a^hIDO1 IC_50_ values are the mean of three independent assays.

For additional validation of the IDO1 inhibitory activity of the selected compounds, we measured their IC_50_ values using kynurenine assay (Table S1)^27–30^. The IC_50_ values of the compounds were directly calculated from the amount of kynurenine generated from L-Trp in the presence of IDO1 enzyme. The results revealed that the IDO1 inhibitory activities of the selected compounds are within the range of 60–400 nM and the differences in the IC_50_ values of the compounds are in accordance with that of measured by the spectrophotometric method. However, the differences in the inhibitory activity values between the spectrophotometric and HPLC methods could be because of the reactivity of the *para*-dimethylaminobenzaldehyde (pDMAB) towards the kynurenine under the experimental reaction conditions^26–30^. The kynurenine assay also demonstrated that compounds **4i** and **4k** displayed stronger inhibitory activity (IC_50_ = 60–115 nM) among all the investigated compounds. Overall, both spectrophotometric and kynurenine assays revealed that the inhibitory efficacies of 4,5-disubstituted 1,2,3-triazoles are sensitive to halogenated aryl-substitution at the C5-position. Stronger inhibitory potencies of the 4-carboxylate-5-aryl-2*H*-1,2,3-triazoles and 4-carboxamide-5-aryl-2*H*-1,2,3-triazoles **2j**, **3i**, **3j**, **4i**, **4k**, and **4 l** could be because of both electronic properties of the C4 and C5-substituted 1,2,3-triazole scaffold, interaction of mono-aryl substitution at the C5-position with the residues present within the ‘pocket-A’ and interaction of 1,2,3-triazole moiety with the heme-group of IDO1. The inclination for the chloro/fluoro-substitution(s) of the C5-aryl ring of the 1,2,3-triazoles (**2j**, **3i**, **3j**, **4i**, **4k**, and **4 l**) could be because of the halogen bonding and/or π-stacking interaction with aromatic amino acids (Y126 and F163)^8,19,33^. Interactions of the 1,2,3-triazole ring, 4-carboxylate, 4-carboxamide, or *N*-substituted 4-carboxamide moiety with the polar amino acid residues and heme-group of the IDO1 play crucial roles for their inhibitory activities. It is essential to mention that these 4,5-disubstituted 1,2,3-triazole based potent compounds showed much stronger IDO1 inhibition activities than the other reported 1*H*-triazole based IDO1 inhibitors. The potent compounds like **3i** or **4k** were 3-fold stronger IDO1 inhibition activities than the 1*H*-triazole based compound MMG-0358, under the similar experimental conditions^18^.

**Figure 4:**
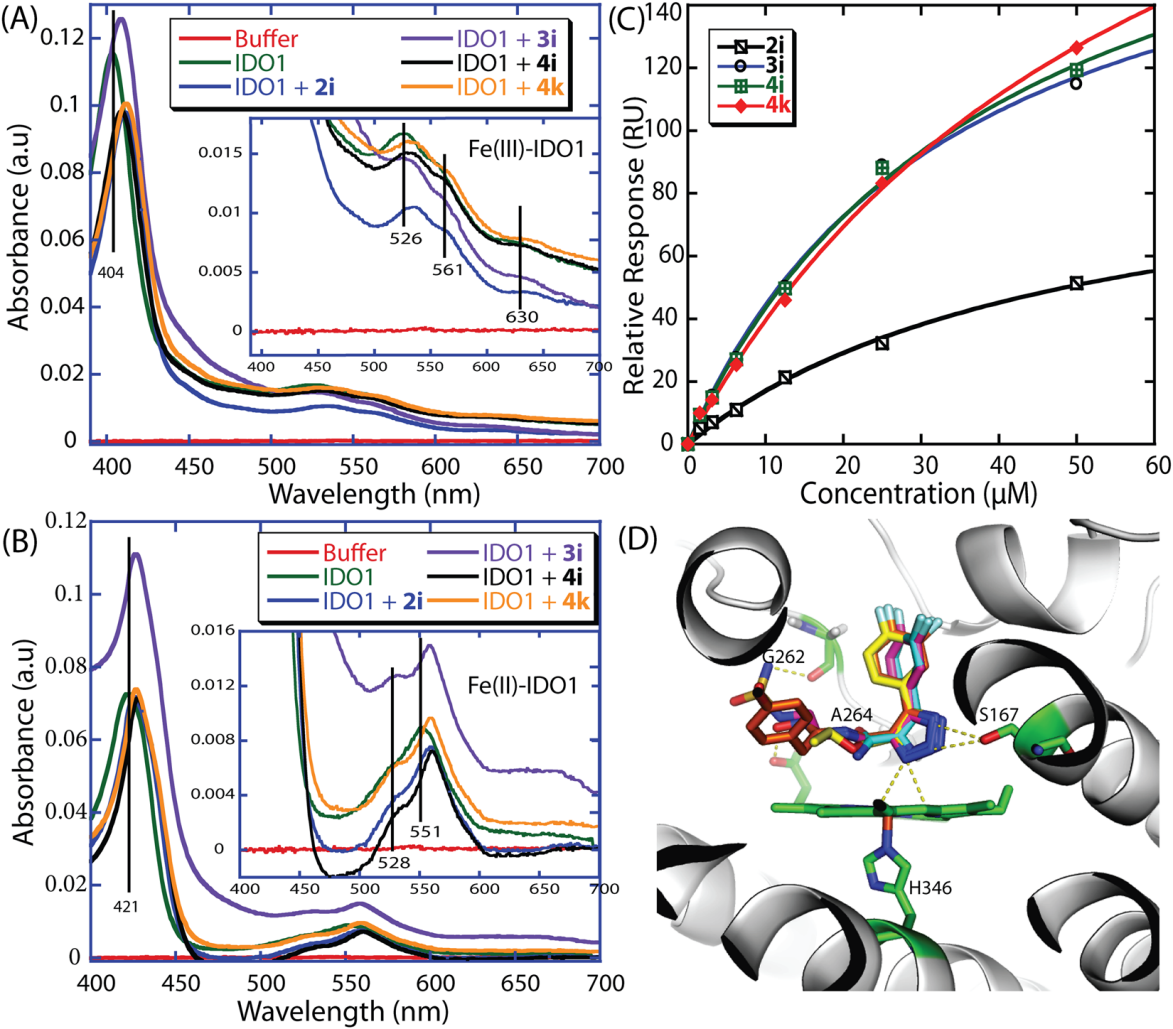
Binding properties of the potent compounds. Absorption spectra of ferric-IDO1 (**A**) and deoxy-ferrous-IDO1 enzyme (**B**) in the absence and presence of the compounds (20 μM) in 100 mM phosphate buffer at pH 6.5. [IDO1] = 0.65 μM. The ferrous-deoxy reaction environment was generated by adding Na_2_S_2_O_4_ to the solution under N_2_ atmosphere. The binding isotherms were generated from the response unit (RU) at 60 sec (average of triplicate measurements) versus the concentration of the compounds. A solid line represents a theoretical curve constructed from Rmax and K_d_ values determined by nonlinear least-squares analysis of the isotherms (**C**). 20 mm HEPES buffer, pH 7.4, with 0.16 m KCl was used for all SPR measurements. Probable mode of interactions (**D**) of the compounds **2i**, **3i**, **4i**, and **4k** with the active site of the IDO1 enzyme (4PK5). The model structures were generated using MoleGro Virtual Docker, version 6.0. The oxygen and nitrogen atoms are shown in red and blue, respectively. Residues involved in interactions through hydrogen bond formation are shown using dashed lines (yellow). Images were generated using PyMol.

The inhibitory activities of the compounds demonstrated their aptitude in inhibiting IDO1 activity but unsuccessful in offering any direct evidence of compound binding to the IDO1 protein. In this regard, the IDO1 binding efficiency of the selected compounds was examined by UV-Vis spectral analysis and surface plasmon resonance (SPR) measurements. The optical absorption spectra of the heme-group are highly sensitive to the local environment and provide substantial evidence of the ligand-binding aptitude to the IDO1 enzyme^26^. The distinctive heme-peaks in the absorbance spectra of the IDO1 enzyme have been used to authenticate a direct binding of the ligand to the heme-containing active site. The UV-Vis spectra of ferric-IDO1 and deoxy-ferrous-IDO1 were recorded in the absence and presence of the selected compounds. However, for both ferric-IDO1 and deoxy-ferrous-IDO1 enzyme, the Soret band got red-shifted by 5–8 nm (Fig. 4A and Table S2) in the presence of 4,5-disubstituted 1,2,3-triazole (**2i**, **3i**, **4i**, and **4k**). For deoxy-ferrous-IDO1 enzyme there was the Q-band (at 551 nm for only enzyme) got shifted by 10–13 nm in the presence of the compounds (Fig. 4B and Table S2). These UV-Vis spectral properties indicate direct binding of the 4,5-disubstituted 1,2,3-triazoles with the IDO1 enzyme. Additional SPR analysis was performed to measure direct interaction between these selected triazoles and IDO1 enzyme^34,35^. The binding affinity (K_d_) between 4,5-disubstituted 1,2,3-triazoles (**2i**, **3i**, **4i**, and **4k**) and IDO1 were within the range of 34–61 μM (Figs. 4C and S1; Table S3). Hence, both UV-Vis spectral analyses and SPR measurements undoubtedly ascertain the binding of compounds to the IDO1 enzyme.

The enzyme inhibition studies indeed describe that the compounds interact with the IDO1 protein. SPR studies also support their protein binding properties. Whereas, spectroscopic measurements in the absence and the presence of the compounds demonstrate that compounds directly interact with the heme-group present in the active site of IDO1 and alter its L-Trp catabolic activities. With the purpose of understanding the molecular determinants that regulate the inhibitory activity of these triazoles, molecular docking analyses were performed using the X-ray co-crystal structure of IDO1 complex (PDB code: 4PK5)^36^. The model structures suggest that the potent triazole-based compounds **2i**, **3i**, **4i**, and **4k** have a very similar pattern of interactions with the IDO1 protein. The molecular docking analysis proposed that the C5-aryl ring of these selected triazoles interacts with the protein ideally through its hydrophobic ‘pocket A’ in the distal heme site. The C5-aryl ring and the halogen substitutions in this ring could assist the compounds in interacting with the amino acids like Y126, F163, F164, and others present in ‘pocket A’ through hydrophobic, halogen bonding and π-stacking interactions^8,19,33^. The triazole ring could be involved in interaction with the heme-group and S167 amino acid residue. This proposed mode of interactions is in good agreement with the UV-Vis spectroscopic based binding studies. However, the C4-substituted moiety of these triazoles could interact differently though the hydrophilic ‘pocket B’ of the protein. The carbonyl group of the 4-carboxylate moiety of the compound **2i** could interact with the backbone NH-proton of A264 residue through H-bonding. The 4-carboxamide group of the compound **3i** could interact with the backbone NH-proton of A264 residue and porphyrin ring through H-bonding (Fig. 4D). The 4-carboxamide, and *N*’-hydroxy-2-(4-hydroxyphenyl)acetimidamide moieties of the compound **4i** could interact with the backbone carbonyl group of F226 and G262 residues, backbone NH-proton of A264 residue and 7-propionate of the porphyrin ring through H-bonding (Fig. 4D).

For compounds **4k**, the 4-carboxamide, and sulfamide moieties could interact with the backbone carbonyl group of G262 and backbone NH-proton of A264 residue through H-bonding (Fig. 4D). Therefore, non-covalent interactions such as H-bonding, halogen bonding, π-stacking, and hydrophobic interactions play decisive roles in the stronger binding of these triazoles to the IDO1 protein through its active site. Overall, the modes of interactions of the compounds with IDO1 are very similar. However, the variations in inhibitory activities suggest that their true-mode of protein interaction could be diverse than these probable ones obtained from the molecular docking analysis (Table S4). In addition to the substitution effects on the 4,5-disubstituted 1,2,3-triazole scaffold their overall molecular volume and pattern of interactions could be also critical for their interactions under physiological conditions. To understand whether the compound binding induces any structural changes of the IDO1 enzyme, we performed circular dichroism (CD) study. The presence of secondary structural elements of the IDO1 enzyme is in good agreement with the previous studies^37,38^. The CD results showed that binding of the compounds to the IDO1 enzyme causes no/negligible changes to its secondary structural elements (Table S5).

**Figure 5:**
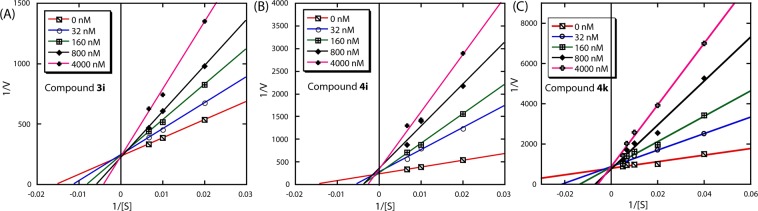
Determination of mode of IDO1 inhibition of the selected compounds. The plot of 1/V against 1/[S] at different concentrations of the compounds **3i** (**A**), **4i** (**B**), and **4k** (**C**). Concentrations of L-Trp were 0, 50, 100 and 150 μM. The concentrations of compounds were varied from 0.032 to 4 μM. All the absorption measurements were performed in 100 mM phosphate buffer pH 6.5 at room temperature.

The IDO1 inhibition and binding studies described that the selected triazoles bind to the active site of the IDO1 enzyme and actively inhibit its L-Trp catabolic activity. To identify the mode of enzyme inhibition by these compounds, we performed standard enzyme kinetics measurements in the absence and presence of these triazoles. The modes of IDO1 enzyme inhibition by these compounds were investigated using the standard Lineweaver-Burk plot. The plots of 1/V against 1/[S] showed that compounds **2i**, **3i**, **4i**, and **4k** followed competitive inhibition modes with respect to L-Trp (Figs. 5 and S2; Table S6). Where, V and [S] represent the initial reaction rate and the substrate concentration, respectively^28,30,34^. Nevertheless, this calculated competitive mode of IDO1 inhibition by the selected compounds may not exhibit their true-mode of enzyme inhibition. Detailed mechanistic studies of the IDO1 assisted catabolism of L-Trp in demonstrating that the generation of ferric-superoxide intermediate is the prime necessity for the subsequent oxidation process, suggesting both O_2_ and L-Trp as substrate for the enzyme. Henceforth, supplementary enzyme kinetic studies regarding O_2_ are required to recognize the precise mode of IDO1 enzyme inhibition^8,26–28,30^.

To investigate the selectivity of these triazoles at inhibiting the IDO1 enzyme, we also performed inhibitory activity against the tryptophan 2,3-dioxygenase (TDO) enzyme, which is also a member of the same enzyme family as IDO1. TDO enzyme also catalyzes the oxidation of L-Trp to *N*-formylkynurenine through the kynurenine pathway. TDO directly controls the >95% metabolism of *L*-Trp in liver. However, TDO is a homotetrameric enzyme, and its active site preferably binds to L-Trp and indole-related derivatives. However, the active site of IDO1 enzyme is more responsive and can adjust different types of small molecules. Both spectrophotometric and kynurenine assay revealed that these selected triazoles **2i**, **3i**, **4i**, and **4k** have only moderate to poor inhibitory efficacies against TDO enzyme, suggesting their higher selectivity for IDO1 over TDO enzyme (Table S7). The IC_50_ values of the compounds against TDO enzyme could not be adequately measured because of weaker and/or non-specific inhibitions (Table S9).

The indoleamine 2,3-dioxygenase 2 (IDO2) is a paralogue of IDO1 enzyme, and it also takes part in the L-tryptophan catabolism process through the kynurenine pathway. However, the IDO2 shows a low L-tryptophan affinity and low turnover rate. The cellular expression of IDO2 enzyme does not influence the tryptophan/kynurenine ratio at physiological tryptophan concentration. Until recently, there is no report that IDO2 reduces tumor volume. Therefore, neither IDO2 inhibitors were systematically developed nor it’s biological activates investigated. The structural investigation also revealed that the hIDO2 differs from hIDO1 by four amino acid side chain: Tyr126, Cys129, Phe164, and Ser167 residues of hIDO1 are replaced by His130, Leu133, Ile168 and Thr171 residues of hIDO2. Overall, IDO2 shares only 44% sequence homology with IDO1. As a result, the volume of ‘pocket A’ is around 15% larger in IDO2 than that in IDO1, which facilitates binding of larger (in size) inhibitors leading to the decrease in IDO2 selectivity. A recent study showed that 4-aryl-1,2,3-triazole derivatives moderately inhibit the hIDO2 activity (IC_50_ > 50 μM) and have only 2-fold selectivity over hIDO1 enzyme^19^. The structural investigation of our synthesized 4,5-disubstituted 1,2,3-triazoles derivatives revealed that few of these compounds could also inhibit the activity of IDO2 enzyme. However, due to lack of therapeutic value of IDO2 its inhibitory activities in the presence of our synthesized compounds were not investigated.

In addition, to explore whether the plasma protein would bind to these aqueous soluble compounds and regulate its biological activities, the serum binding properties of these potent compounds were qualitatively investigated using Trp-fluorescence analysis. The extent of Trp-fluorescence quenching ability of these compounds suggests that compounds **2i**, **3i**, and **4k** interacts more strongly with the human serum proteins than compounds **4i** (Fig. S3). The outcomes of this binding analysis suggest that the serum proteins could assist the transport of the compounds via blood to the sites of action throughout the body^39^.

To investigate the biological activities of these 4,5-disubstituted 1,2,3-triazoles under cellular environment, cytotoxicity and cellular IDO1 inhibitory activities were performed in MDA-MB-231 cells. The MTT assay of the compounds showed low level of cytotoxicity of the compounds both in HEK-293 and MDA-MB-231 cells (Figs. S4 and S5). Interferon-gamma (IFN-γ) is known to induce the expression of the native IDO1 enzyme from its mRNA in the MDA-MB-231 cells^27,29,40^. Hence, IDO1 inhibitory activities of the 4,5-disubstituted 1,2,3-triazoles were measured using IFN-γ induced MDA-MB-231 cells. Nevertheless, IDO1 inhibitory activities under the cellular conditions follow a comparable pattern as that of measured against the purified IDO1 enzyme. The measured EC_50_ values of the potent compounds were within the range of 60–422 nM (Table 3 and S10). Control compound, 4-amino-*N*-(3-chloro-4-fluorophenyl)-*N*′-hydroxy-1,2,5-oxadiazole-3- carboximidamide and MMG-0358, showed EC_50_ values of 63 and 120 nM, under similar experimental conditions, which are in agreement with the reported values^18,28,30,31^. However, smaller variations in the compound’s IDO1 inhibitory activities between the two activity assay systems, against the purified enzyme and under the cellular conditions, could be primarily due to the obstacles in regulating the redox activity of the enzyme and/or environmental effect on the assay systems. Overall, a good data correlation between these assays corroborates the IDO1 inhibitory efficiencies of these selected triazoles.

**Table 3:** IDO1 inhibitory activity of the selected compounds under the cellular environments.

Compound	MDA-MB-231 cells^a^ EC_50_ (μM)^b^
2i	0.162 ± 0.015
3i	0.074 ± 0.017
4i	0.088 ± 0.010
4k	0.077 ± 0.005

^a^IDO1 protein expression in MDA-MB-231 cells was induced by human IFN-γ (50 ng/mL).

^b^EC_50_ values are the mean of three independent assays.

**Figure 6:**
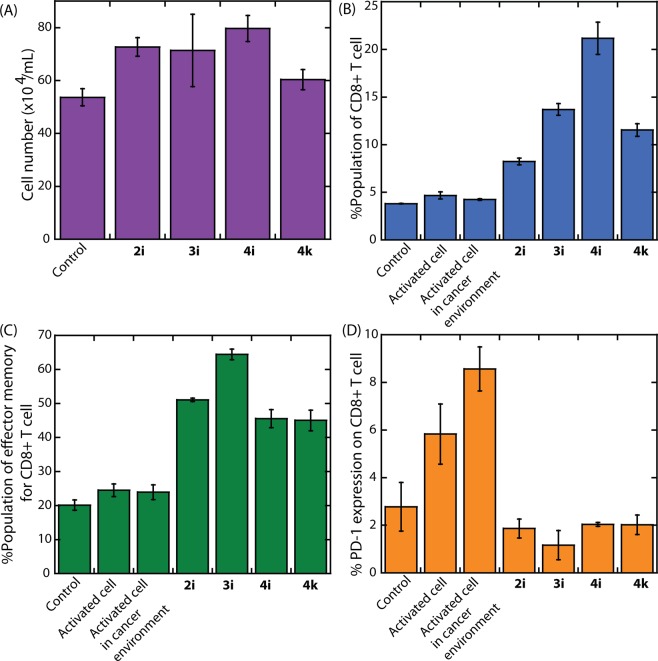
T cell activity studies in the presence of the compounds. The proliferation of the peripheral blood mononuclear cells (PBLs; P = 0.0075) in the presence of the compounds. (**A**) Regulation of the CD8+ T cells in the tumor micro-environment (P < 0.0001) in the presence of the compounds. (**B**) Regulation of effector memory CD8+ T cells in tumor micro-environment (P < 0.0001) in the presence of the compounds. (**C**) Regulation of the PD-1 expression on CD8+ T cells in tumor micro-environment (P < 0.0001) in the presence of the compounds. (**D**) The results were found statistically significant post statistical analysis with one way ANOVA. The concentration of the compounds was 50 μM for all the T cell activity studies.

The above experimental evidence confirmed that the selected 4,5-disubstituted 1,2,3-triazole derivatives are potent inhibitors of the IDO1 enzyme. It is well documented that the immunoregulatory enzyme IDO1 promotes immunologic tolerance in tumor-microenvironment^41^. The macrophages or dendritic cells present in peripheral blood lymphocytes (PBL) also produce IDO1 enzyme that also inhibits the T cell activation and proliferation^42^. In the tumor-microenvironment the immune suppression follows several paths. The CD8 + T cells are one of the critical immune cells that promote tumor rejection. However, dysfunction of cytotoxic CD8 + T cells is known to elevate the immune evasion of tumor cells. The IDO1-mediated Trp metabolites were reported to be protective against T cell-mediated fetal rejection^43^. The IDO1 inhibition has shown protective effect and boosts up CD8 + T cells activity against host influenza viral infection^44^. However, the effect of IDO1 inhibition on rejuvenation of CD8 + T cells in the tumor micro-environment of the breast cancer cells have never studied before. Hence, to explore the therapeutic potential of these selected IDO1 inhibitors, we explored the activity of the cytotoxic T cells in breast tumor micro-environment. Initially, freshly isolated PBLs were cultured in RPMI with 10% FBS and treated with IDO1 inhibitor for 48 hours. The compound treatments showed significant up-regulation in PBL cells than the control group (Fig. 6A, P < 0.05). Since, IDO1- mediated Trp metabolism promotes dampening of CD8 + T cell responses; we sought to check whether IDO1 inhibitor treatment elevates proliferation of cytotoxic T cells in the tumor micro-environment. The PBLs were cultured in 20% conditioned medium of human metastatic breast cancer cell line MDA-MB-231 for 48 hours. Prior cultures the cells were allowed to be stimulated with PMA and ionomycin. The outcome of the FACS analysis showed that the treatment of IDO1 inhibitors significantly induced CD8 + T cell proliferation (Figs. 6B and S6; P < 0.001). The increase in total CD8 + T cell number inspired us to investigate whether the potent IDO1 inhibitors are regulating the effector memory phenotype of cytotoxic T cells or not. The CD8 + T cells were phenotyped for CCR7 and CD45RA expression, where CCR7^low^CD45RA^low^ phenotype is designated as effector memory phenotype. Two days post-treatment with IDO1 inhibitors a remarkable aggrandize effector memory phenotype of cytotoxic T cells (Figs. 6C and S7; P < 0.001) was observed. These results support IDO1 mediated abrogation of effector memory CD8 + T cell production. Tumor cells evade cytotoxic T cells mediated rejection due to the enhanced dysfunction in the tumor-reactive T cells. Germination of T cell dysfunction is believed to be mediated by the elevation in surface protein PD-1 in cytotoxic T cells. Detailed studies demonstrated that the binding of surface protein PD-L1 of the tumor cells to PD-1 or B7.1 of the T cells conveys an inhibitory signal that decreases the proliferation of antigen-specific T-cells in lymph nodes. The activation of cytotoxic T cells by PD-1/PD-L1 blockade therapy has astonishing responses in different cancer patients. Surprisingly, in breast cancer the effect of PD-1/PD-L1 blockade therapy is not so encouraging. The germination of malfunctioned cytotoxic T cells in breast cancer could be one of reasons. In this study, we investigated whether IDO1 inhibition attenuates the boosted PD-1 expression of CD8 + T cells in breast tumor micro-environment. The outcome of the studies showed that the IDO1 inhibition prodigiously suppressed the PD-1 level of cytotoxic T cells in the tumor micro-environment (P < 0.001), suggesting that these compounds may rejuvenate the cytotoxic T cells in breast cancer (Figs. 6D and S8). Hence, the results from this study depict that IDO1 regulation by these potent inhibitors can elevate aspects of adaptive immune response against breast cancer.

**Figure 7:**
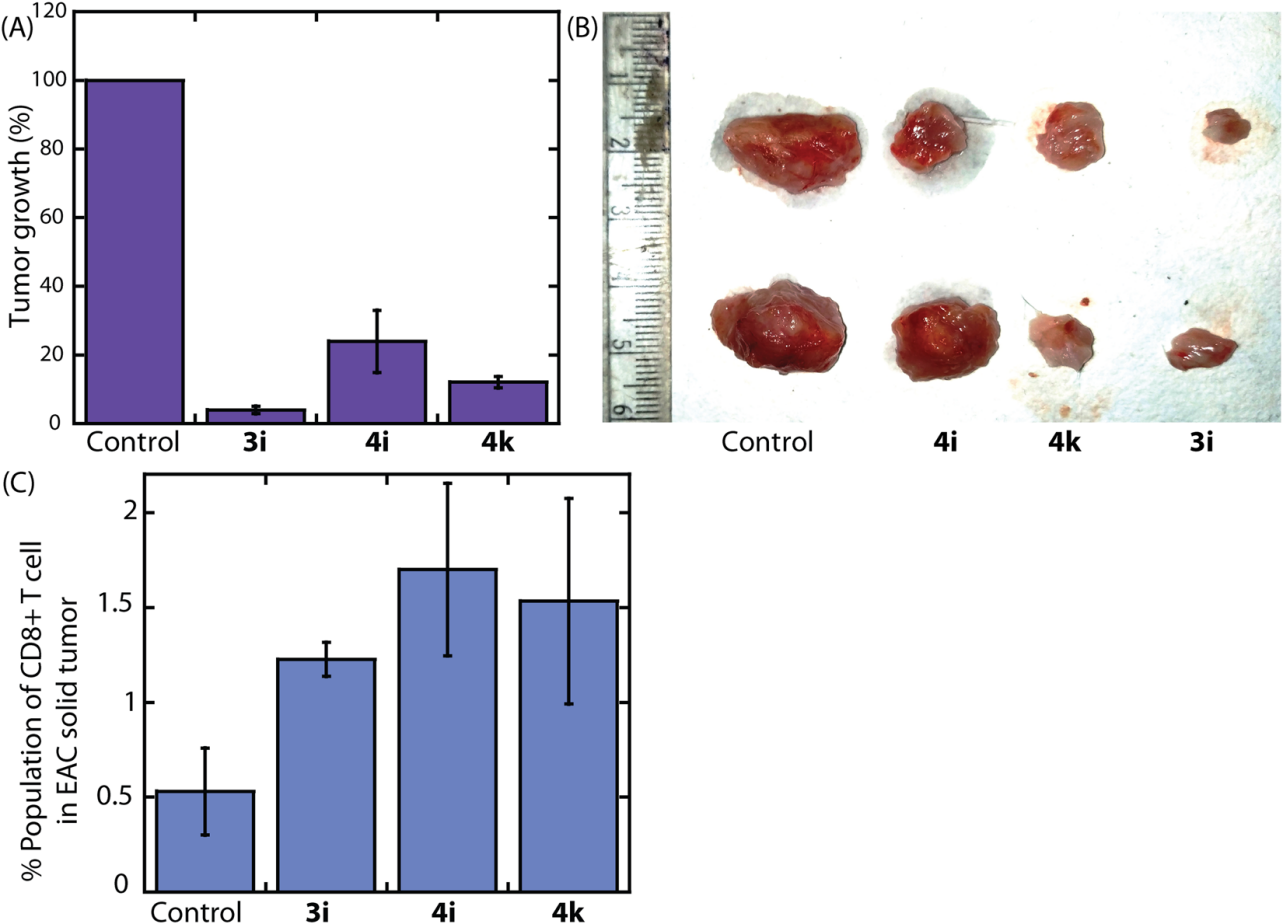
The effect of compounds (5 mg/kg body weight) on the growth of EAC solid tumor model in female Swiss albino mice (n = 6; **A**,**B**). The compounds were injected intravenously at alternate days from the 5^th^ day of the tumor implant. CD8+ T cell population in *vivo* solid tumor (**C**).

These IDO1 inhibitors promoted enhancement of CD8+ T cell activities inspired us to investigate the *in vivo* antitumor efficacy in female Swiss albino mice^45^. For the *in vivo* experiments the EAC solid tumor model was used to understand the effect of IDO1 inhibition on tumor burden. The EAC solid tumor model is popular and well recognized *in vivo* tumor model for anti-tumor therapy^46–48^. As shown in Fig. 7, the treatment with compounds **3i**, **4i** and **4k** showed remarkable regression in tumor growth with TGI = 79–96%. Compound **3i** was most effective in attenuating tumor growth with TGI = 96%. Post-treatment tumor tissues were found to have high infiltration of CD8+ T cells (Figs. 7C and S9)^45,49^.

This study describes the design and synthesis of 4,5-disubstituted 1,2,3-triazoles as IDO1 enzyme inhibitor. Consequential modification of the electronic properties of the 1,2,3-triazole scaffold allowed us to pinpoint potent compounds with nanomolar-level IDO1 enzyme inhibitory efficacies under the *in vitro* conditions. Both, spectrophotometric and HPLC-based kynurenine assays revealed that the presence of dihalogensubstituted aryl ring, 4-carboxylate, 4-carboxamide, and hydroxyamidine or sulfamide modified 1,2,3-triazole moieties could substantially augment the inhibition effectiveness of these triazoles. Spectroscopic studies and SPR analysis confirmed that the selected triazoles interact with the IDO1 enzyme. Molecular modeling studies proposed that the electronic properties of the substituents at the C4- and halogen-substituted aryl ring at the C5- position of the triazole scaffold assist these compounds in binding to the IDO1 enzyme through non-covalent interactions including hydrogen bonding, halogen bonding, hydrophobic and pi-stacking interactions. Calculated inhibitory constant (*K*_i_) values of these potent compounds are within range of 98–334 nM range (Table S5). These compounds also showed stronger inhibitory activity for the IDO1 enzyme than the TDO enzyme under similar experimental conditions. The MTT and cellular IDO1 activity assays showed that these compounds have trivial cytotoxicity and low-nanomolar level inhibitory activities, respectively. Until now, limited triazole-based compounds have been described with such selective and stronger IDO1 enzyme inhibitory activities. The T cell activity studies identified that the IDO1 enzyme has an immune-damping effect in breast tumor micro-environment, which can be reversed by the treatment with IDO1 inhibitor. The IDO1 inhibitor treatment uplifts the total CD8 + T cell number in tumor micro-environment and elevates its effector memory phenotype. The IDO1 inhibitor also attenuates PD-1 expression on cytotoxic T cells, which could help reverse the immune dormancy of these CD8 + T cells to show their effector function. The observations from these study findings clarify that controlled regulation of IDO1 enzyme can elevate the robustness of cytotoxic T cell response, attributing a better tumor regression and progression-free survival of breast cancer. Intravenous treatment of the compounds showed excellent *in vivo* antitumor efficacy in the female Swiss albino mice. These results suggest that 4,5-disubstituted 1,2,3-triazole derivatives represent a promising class of IDO1 inhibitors, but further structural modifications are required to enhance the *in vivo* antitumor efficacy. It is important to mention that, although we have chemically synthesized and characterized a series of 4,5-disubstituted 2*H*-1,2,3-triazole but multiple tautomers of 2*H*-triazoles may exist in aqueous medium, and same is applicable for other reported 1*H*-triazoles^18,19^.

## Conclusion

Developments of small molecules based IDO1 inhibitors are becoming increasingly important, because of the crucial role of the IDO1 enzyme in cancer immunotherapy. In this report, we described the synthesis of a series of 4,5-disubstituted 1,2,3-triazole derivatives. Comprehensive biochemical, biophysical, cellular studies identified triazole-based compounds as potent inhibitors of IDO1 enzyme. The presence of dihalogen containing aryl ring and suitably substituted 4-carboxylate or 4-carboxamide moieties could be the key factor for the inhibitory efficacies of these triazoles. The potent compounds up-regulated the proliferation, activity and effector memory of the CD8 + T cells in the tumor micro-environment. An excellent *in vivo* antitumor efficacy in the female Swiss albino mice was observed in the presence of these compounds. Overall, these findings suggest that suitably substituted 4,5-disubstituted 1,2,3-triazole derivatives are potent inhibitors of IDO1 enzyme and could be of interest as drug targets in cancer and other life-threatening diseases.

## Methods

### General information

All reagents were purchased from different commercial sources and used directly without further purification. Reactions were monitored by thin-layer chromatography (TLC) on silica gel 60 F254 (0.25 mm). ^1^H NMR and ^13^C NMR were recorded at 400 and 100 MHz, respectively, with Varian AS400 spectrometer and 600, 151, 100, 75 MHz, respectively, with Brucker spectrometer, using TMS as an internal standard with CDCl_3,_ DMSO-*d*_6_, D_2_O, and MeOD-d_4_. The coupling constant (*J* values) and chemical shifts (*δ*_ppm_) were reported in Hertz (Hz) and parts per million (ppm) respectively. Multiplicities are reported as follows: s (singlet), d (doublet), t (triplet), q (quartet), m (multiplet), and br (broadened). High-resolution mass spectra (HRMS) were recorded at Agilent Q-TOF mass spectrometer with a Z-spray source using built-in software for analysis of the recorded data. Melting point data were recorded at Buchi Melting Point B-540 apparatus. All the experimental procedures and protocols were followed in accordance with the institutional guidelines and regulations.

### Synthesis of the compounds

#### General procedure for the synthesis of N-tosyl aryl hydrazones

To a stirring solution of aromatic aldehyde (3 mmol) in ethanol (6 mL) was added *p*-toluenesulfonyl hydrazide (3.3 mmol) and allowed to stir for two hours at room temperature^20^. The progress of the reaction was monitored by TLC. After completion of the reaction, the reaction mixture was cooled down to room temperature, and the solvent was removed under reduced pressure. The observed solid product was washed with ethanol and dried under reduced pressure.

#### General procedure for the synthesis of hydroxyamidines derivatives

To a stirring solution of substituted benzonitrile/phenylacetonitrile (4 mmol) and hydroxylamine hydrochloride (6 mmol) in MeOH (5 mL) was added triethylamine (14 mmol), and the mixture was allowed to reflux at 60 °C for 12 hours^29^. The progress of the reaction was monitored by TLC technique. After completion of the reaction, it was cooled down to room temperature, and the solvent was removed under reduced pressure. Then the reaction mixture was diluted with ethyl acetate. The organic layer was extracted and washed with brine and dried over anhydrous Na_2_SO_4_. The organic solvent was further removed under reduced pressure. The reaction mixture was purified by column chromatography.

### Expression and purification of recombinant human IDO1 and TDO enzymes

Expression and purification of the enzymes were carried out as described previously with the following minor modifications^26–30,50,51^. Briefly, *N*-terminus 6× -histidine tagged recombinant human IDO1, and TDO enzymes were expressed by *Escherichia coli* (M15 cell for IDO1 and BL21 (DE3) cell for TDO) using cDNA of human IDO1 (in the vector pQE30 and pREP4 plasmid) and TDO (in the vector pET 28a) respectively. A single colony of *Escherichia coli* cells with cDNA of the mentioned enzymes was inoculated in 5 mL of Luria-Bertani (LB) medium containing appropriate antibiotics (i.e. 100 µg/mL ampicillin, 50 µg/mL kanamycin for IDO1 and 50 µg/mL kanamycin for TDO enzyme) and the cells were grown for overnight at 37 °C and 180 rpm. Then, 1 mL of this overnight culture was added to 1 L of the same culture media and incubated at 37 °C and 120 rpm to achieve an OD_600_ of 0.6 to 0.9 of the culture media. The culture was then cooled down, and protein expression was induced by the addition of isopropyl β-D-1-thiogalactopyranoside (IPTG), hemin, and phenylmethylsulfonyl fluoride (PMSF) with the final concentration of 1 mM, 7 µM and 1 mM, respectively. Induced cells were grown for 3 hours at a specific temperature (i.e. 25 °C for IDO1 and 22 °C for TDO) and 120 rpm. After this incubation period ethylenediaminetetraacetic acid (EDTA) was added to the culture at a final concentration of 1 mM and cells were grown for another 12 hours. The cells were collected by centrifugation at 5000 rpm for 10 minutes (at 4 °C). The cell pellet was re-suspended in 20 mL of ice-cold phosphate-buffered saline (PBS) containing 1 mM PMSF and then centrifuged at 15000 rpm for 10 minutes at 4 °C to remove EDTA and stored at −80 °C.

For the purification of the enzymes from the *Escherichia coli* cells the stored pellet was re-suspended in 15 mL of ice-cold 50 mM potassium phosphate buffer (KPB) at a specific pH (i.e. pH 6.5 for IDO1 and pH 8.0 for TDO) containing 300 mM potassium chloride (KCl), 10 mM imidazole, 10 mM magnesium chloride (MgCl_2_), protease inhibitors (complete EDTA free) and DNase (<1 mg). Cell membranes were disrupted on ice, and the lysate was centrifuged at 20,000 rpm for 30 minutes at 4 °C followed by filtering through 0.22 µm filter. Then, 1 mL of nickel-nitrilotriacetic acid resin (Qiagen) was added to the clear supernatant, and the mixture was incubated for 2 hours at 4 °C and 80 rpm. After that the mixture was poured into the column (which was equilibrated with 50 mM KPB containing 300 mM KCl and 10 mM imidazole at specific pH (i.e. pH 6.5 for IDO1 and pH 8.0 for TDO)). For IDO1 enzyme column was sequentially washed with 10 mL of KPB at pH 6.5 containing 300 mM KCl and 20 and 30 mM imidazole, respectively to remove the non-specifically bound protein. The IDO1 protein was eluted with 50 mM KPB at pH 6.5 containing 300 mM KCl and 190 mM imidazole. For TDO enzyme column was sequentially washed with 10 mL of KPB at pH 8.0 containing 300 mM KCl and 20 and 30 mM imidazole, respectively, to remove the non-specifically bound protein. The TDO protein was eluted with 50 mM KPB at pH 8.0 containing 300 mM KCl and 190 mM imidazole. Both IDO1 and TDO proteins were then buffer exchanged by 50 mM Tris-buffer at pH 7.4 and 50 mM Tris-buffer at pH 8.0, respectively, using Sephadex-G25 column. The purity of the enzyme was confirmed by Coomassie-blue stained SDS-PAGE analysis, and it showed 85–90% purity. The ratio of absorbance of the purified IDO1 and TDO enzyme at 404 nm to that at 280 nm was around 1.3 and 1.2, respectively.

### IDO1 and TDO inhibition assay by the spectrometric method

Both IDO1 and TDO inhibition assays were performed according to the reported procedures^26–30,50,51^. The solubility of the compounds in water was either moderate or poor. Hence, stock solutions of the compounds were prepared by first dissolving the compounds in DMSO and then diluted with buffer. In the assay system, the minimum and maximum amount of DMSO was 0.02% and 2%, respectively. The standard reaction mixture (500 μL) containing KPB (100 mM, pH 6.5 for IDO1 enzyme and 50 mM, pH 8.0 for TDO enzyme), sodium ascorbate (20 mM), methylene blue (10 µM), catalase (240 nM, from bovine liver), L-Trp (150 µM), purified enzyme (40 nM for IDO1 and 25 nM for TDO), DMSO (0.05%, v/v), Triton-X 100 (0.01%, v/v) and inhibitors was incubated at 37 °C for 1 hour. The concentration of the inhibitors was varied from 100 µM to 32 nM by serial dilution technique. Then, the reaction was quenched with 100 µL of 30% (w/v) trichloroacetic acid and was incubated for further 15 minutes at 65 °C. After that, 2% (w/v) pDMAB was used to quantify the amount of kynurenine formation. The absorbance of the reaction mixture was recorded by UV-Vis spectrophotometer at 480 nm. The K_m_ = 59.76 µM and K_cat_ = 6.18 sec^−1^.

### Kynurenine assay by HPLC analysis

HPLC based IDO1, and TDO inhibition assays were performed according to the earlier reported procedure^19,27–30^. The reaction mixture (250 µL) contained KPB (100 mM, pH 6.5 for IDO1 and 50 mM, pH 8.0 for TDO), sodium ascorbate (20 mM), methylene blue (10 µM), catalase (240 nM, from bovine liver, Sigma), L-Trp (150 µM), purified enzyme (40 nM for IDO1 and 25 nM for TDO), DMSO (0.05%, v/v), Triton-X 100 (0.01%, v/v) and inhibitors. The concentration of the compound was varied from 100 µM to 32 nM by serial dilution. The mixture was incubated at 37 °C for 1 hour in the absence and presence of the synthesized compounds. The reaction was then quenched using 50 µL of 30% (w/v) trichloroacetic acid and incubated at 65 °C for additional 15 minutes to allow complete hydrolysis of *N*-formylkynurenine to kynurenine. After that, the mixture was centrifuged at 10,000 rpm for 10 minutes, and 100 µL of clear solution was transferred to another tube for HPLC analysis. Then, 20 µL of the reaction mixture was then injected through Ascentis® express C18, 2.7 µm HPLC column with a flow rate of 0.5 mL/minute of the mobile phase containing 50% sodium citrate buffer (40 mM, pH 2.25) and 50% methanol (v/v) with 400 µM SDS. The area under the curve at 365 nm chromatogram corresponding to the kynurenine formation was recorded, and % of inhibition was calculated using standard curve prepared with pure kynurenine (from Sigma) under similar experimental conditions.

### Binding analysis by spectroscopic measurement

The efficacy of the compound’s direct binding to the enzyme active site was measured by UV-Vis spectroscopic analysis^26–28,30^. All the measurements were performed at room temperature using 100 mM KPB (pH 6.5), purified hIDO1 enzyme (3 µM), and selected compounds (20 µM. The deoxy-reaction system was generated by injecting Na_2_S_2_O_4_ (~10-fold excess) into the solution pre-purged with N_2_ gas.

### Binding analysis by Surface Plasmon Resonance (SPR) analysis

The surface plasmon resonance (SPR) analysis was carried out according to the reported procedure with minor modifications^34,52^. In brief, the measurements were performed at 25 °C on the Biacore-X100 instrument using CM5 sensor chip (GE Healthcare). PBS buffer at pH 7.4 was used as running buffer. The surface of the CM5 sensor chip was first modified with Anti-His Antibody using amine-coupling kit at a flow rate of 30 µL/ minute, followed by IDO1 enzyme (30 µg/mL) immobilization using His-capture kit at a flow rate of 10 µL/minute for 2 minutes. The IDO1 enzyme capture level was 4000 response units (RU). The binding efficacy (association constant; *k*_*a*_) of the compounds was determined by measuring the change in RU of the SPR sensorgram in absence or presence of the compounds (0 to 50 µM).

### Molecular docking analysis

MoleGro Virtual Docker version 6.0 (MoleGro Aps, Aarhus, Denmark) was used for molecular docking analysis of the compounds with IDO1 enzyme (PDB code: 4PK5)^27,30,36^. The apo-protein was generated and processed by energy minimization. The energy minimized 3D-structure of the ligand was prepared by the Automated Topology Builder (ATB) and Repository server (http://atb.uq.edu.au/). The occupied position of the ligand (in the crystal structure) was used as the center of docking site (radius: 12 Å; and center: x = 61, y = 51, z = 19), and the other parameters were set default during docking analysis. Two hundred docked structures were generated in each docking run for an individual ligand, and energetically favored docked conformations were evaluated based on the moledock and re-rank scores (docking score-based on energy function such as a force field with repulsive and attractive van der Waals terms and electrostatic term). The docking poses were analyzed using PyMOL software (The PyMOL Molecular Graphics System, Version 1.0r1, Schrödinger, LLC).

### Circular Dichroism analysis

The Circular Dichroism (CD) spectra of the IDO1 enzyme in the absence or presence of compounds were recorded with a Jasco-810-spectropolarimeter according to the reported procedure^37,38^. Samples of rIDO1 (2 μM) were prepared in 10 mM KPB buffer (pH 6.5) in a 1 mm path length quartz cuvette. Spectra were recorded by varying the concentration of the compound (250 nM to 4 µM) using a 1 mL quartz cuvette of 1 cm path length with a Jasco J-1500 spectropolarimeter at 17 °C. Also the spectra were collected at a scan rate 100 nm/sec and 1 nm bandwidth from 200 nm to 260 nm with three times scan for averaging. All final spectra were corrected for buffer background CD signals. The % of the secondary structures of the IDO1 enzyme was analyzed using the inbuilt software of the instrument.

### Determination of mode of enzyme inhibition

The mode of IDO1 enzyme inhibition was measured according to the reported spectrophotometric method^30^. The assay was performed by varying both tryptophan concentration from 50 to 150 µM and inhibitor concentration from 32 nM to 4 µM. The amount of formation of *N*-formylkynurenine was recorded at different time intervals. The mode of enzyme inhibition was determined from the plot of 1/V against 1/[S], where V is the initial rate of the enzymatic reaction and [S] is the L-Trp concentration.

### Analysis of the serum binding ability of the potent compounds

Tryptophan fluorescence measurements of the human serum were performed in the presence of compounds on a Fluoromax-4 spectrofluorometer at room temperature^53^. For fluorescence measurements, 30 µL of 100% human serum and varying concentrations (0–220 µM) of compounds were incubated in 10 mM PBS buffer, pH 7.4. The solution was excited at 290 nm, and emission spectra were recorded from 300 to 560 nm. The resulting tryptophan emission spectra for each concentration of the compounds were plotted.

### Cell viability analysis

Cell viability analysis was performed in HEK-293 and MDA-MB-231 cells by using MTT (3-(4,5-dimethylthiazol-2-yl)-2,5-diphenyltetrazolium bromide) dye. For this experiment, 10,000 cells were seeded in 0.5 mL of DMEM/F12 complete medium, and after 12 hours of incubation cells were washed with cell-culture-grade PBS buffer. After that, the compounds (at a concentration range 32 nM to 100 µM) were added into the incomplete medium and incubated for another 48 hours. After that, 100 µL of MTT dye (0.5 mg/mL in PBS) was added into the culture medium and incubated for 4 hours at 37 °C with 5% CO_2_. Then, MTT solution was removed, and the formazan crystal was dissolved in 100 µL cell culture grade DMSO. The absorbance was determined using spectrophotometer (FLoid*®* Cell Imaging Station) at 570 nm, and 600 nm^27,28,30,52^. All the cell culture related experiments were conducted in the designated biosafety level facility available at the Indian Institute of Technology Guwahati.

### Cellular activity assay

The cellular enzyme activity assay was performed according to the reported procedure with minor modifications^19,27–30,33,54^. Briefly, MDA-MB-231 breast cancer cells were selected for this assay because of the presence of IDO1 mRNA in this cell line. First, cells were grown in DMEM/F12 complete media overnight^40^. Then different concentration of human interferon-gamma (IFN-γ) (from 5–1000 ng/mL) was added to it and incubated for 48 hours. This treatment is reported to allow over-expression of IDO1 enzyme in MDA-MB-231 cells. After that, L-Trp (150 µM) was added to medium and incubated for additional 5 hours. Sterile cell-culture grade PBS was used to wash the cells by centrifugation technique. Then, the pellet was lysed in 10 mM HEPES buffer by passing through a sterile syringe. The lysate was used for standard IDO1 assay as mentioned earlier. The relative amount of *N*-formylkynurenine generated in the presence of the different concentration of IFN-γ was used to optimize the required concentration of IFN-γ for the IDO1 inhibition assay. The result showed that 50 ng/mL of IFN-γ generated the maximum IDO1 enzyme expression level under the experimental conditions and this optimized IFN-γ concentration was used for further IDO1 inhibition assay under the cellular environment. Selected compounds with concentration from 32 nM to 100 µM were used for the inhibition assay (incubated period-5 hours; L-Trp concentration 150 µM). Cells stimulated with IFN-γ alone were used as negative control, while cells stimulated with L-Trp without compound served as positive control. The cells were washed with sterile cell culture grade PBS and lysed in 10 mM HEPES buffer by sterile syringe. The lysate was used for standard IDO1 assay, as mentioned earlier, and the extent of IDO1 enzyme inhibition was determined for the selected compounds.

### T cell activity studies

We designed this study to evaluate the effects of IDO1 inhibition on cytotoxic T cells present in the breast tumor micro-environment. To mimic breast tumor micro-environment, we used 20% conditioned medium of human metastatic breast cancer cell line MDA-MB-231 with fresh RPMI and 10% FBS. Freshly isolated PBL cells were stimulated with PMA and ionomycin prior to culture in 20% conditioned medium and compounds at selected concentrations. Drug treatment was done for 48 hours followed by immunophenotyping by flow cytometry. For cell proliferation experiments freshly isolated PBLs were cultured in RPMI with 10% FBS and inhibitors for 48 hours. After that the cells were counted with hemocytometer. All the experiments were repeated thrice before analyzing data. The concentration of the compounds was 50 μM for all the T cell activity studies. All the T cell culture related experiments were conducted in the designated biosafety level facility available at the University of Calcutta.

#### Peripheral blood lymphocyte (PBL) isolation and culture

Peripheral blood lymphocyte was isolated from freshly isolated blood samples of healthy volunteers in a previously described protocol^55^. Briefly, blood was diluted with equal volume of sterile PBS and allowed in density gradient centrifugation over histopaque. Isolated PBLs were washed and cultured in RPMI medium with 10% FBS. Peripheral blood lymphocytes (PBLs) were isolated from fresh blood of healthy volunteers and cultured in RPMI medium with or without compound at selected concentration for 48 hours. Treatment of the compound uplifted cell proliferation. To evaluate whether 4,5-disubstituted 1,2,3-triazole regulate T cell activity, PBL was isolated and cultured at 20% conditioned medium of human metastatic breast cancer cell line MDA-MB-231. To perform this experiment PBL was isolated from three healthy volunteers and three independent experiments were performed. All the volunteers signed informed consent form^45,49^.

#### T cell activation with PMA and ionomycin

The T cells were activated according to standardized protocol published elsewhere^56^. 100 ng/mL PMA and 1 μg/mL ionomycin were used to stimulate the T cells for 2 hrs. PBLs freshly cultured in RPMI with 10% FBS was added in the culture plate at 2 × 10^5^ cells/ well and cultured at 37 °C with 5% CO_2_ for the duration of activation.

#### Immunophenotyping by flowcytometry

After treatment for given period of time the lymphocytes were analyzed for alteration of protein expression by flow cytometry according to previously published protocols^57^. Briefly the cells were pelleted down resuspended in cell staining buffer. After cell counting cells were stained against PE-CD8 (Miltenyi Bioteh.), PerCP-Cy 5.5-CCR-7 (Miltenyi Bioteh.), APC-CD45RA (Miltenyi Bioteh.), VioBright-FITC-PD-1 (Miltenyi Bioteh.) antibodies as per the manufacturer recommended concentration and incubated in ice for given period of time. Post incubation, the cells were washed, suspended in cell staining buffer before data was acquired using FACS-Verse flow cytometer. The results were analyzed with FlowJo software (v X.0.7). The experiments were repeated thrice prior data analysis.

### *In vivo* activity studies

#### Animals and ethical statement

Female Swiss albino mice of age 6 weeks (weight = 20 gm) were selected for the experiments. Animals were housed in animal facility room, Department of Zoology, University of Calcutta at 25 ± 2 °C, relative humidity of nearly 45 ± 5% with alternative 12 hours day/ night cycle with access of food and water. All the experiments were carried out as per guideline of the Committee for the Purpose of Control and Supervision of Experimental Animals (CPCSEA), Government of India (Registration No: 885/GO/Re/S/05/CPCSEA) and approved by the Institutional animal ethical committee (IAEC), University of Calcutta.

#### Tumor cell preparation and transplantation

Ehrlich ascites carcinoma (EAC) cells were maintained in the ascetic form in female Swiss albino mice by intraperitoneal transplantation every 10^th^ day on each mice. The ascetic fluid was drawn on 7^th^ day on implantation according to previously described protocol^58^. Isolated cells were diluted in sterile PBS. 2 × 10^6^ cells were then implanted on thigh muscle of all the animals selected for experiment.

#### Experiment design

Female Swiss albino mice were divided into four groups (n = 6 each group). Post inoculation of the cells in thigh muscle on day 0 mice in their respective group was allowed to treat for 5 days on every alternate day at treatment of 5 mg/ kg body weight described below. All the mice were sacrificed on day 10.

A. Placebo control group (5% DMSO at 10 mL/kg)

B. Compound **4i**

C. Compound **4k**

D. Compound **3i**

#### CD8+ T cell populations in vivo solid tumors

Tumor tissues post sacrifice were digested in collagenase IV medium for 2 hours. The tissues were passed through wire mesh and single-cell strainer. Then the cells were washed in PBS, and immunophenotyping was performed, and cells were stained against APC-CD8 for finding infiltrated CD8 T cell populations by flow cytometry analysis.

## Supplementary information

Supplementary information

## References

[1] Mahoney KM, Rennert PD, Freeman GJ (2015). Combination cancer immunotherapy and new immunomodulatory targets. Nat. Rev. Drug Discov..

[2] Dunn GP, Old LJ, Schreiber RD (2004). The immunobiology of cancer immunosurveillance and immunoediting. Immunity.

[3] Kershaw MH, Westwood JA, Slaney CY, Darcy PK (2014). Clinical application of genetically modified T cells in cancer therapy. Clin. Transl. Immunol..

[4] Zou WP (2005). Immunosuppressive networks in the tumour environment and their therapeutic relevance. Nat. Rev. Cancer.

[5] Qian S (2016). IDO as a drug target for cancer immunotherapy: recent developments in IDO inhibitors discovery. Rsc Adv..

[6] Okamoto A (2005). Indoleamine 2,3-dioxygenase serves as a marker of poor prognosis in gene expression profiles of serous ovarian cancer cells. Clin. Cancer Res..

[7] Uyttenhove C (2003). Evidence for a tumoral immune resistance mechanism based on tryptophan degradation by indoleamine 2,3-dioxygenase. Nat. Med..

[8] Rohrig UF, Majjigapu SR, Vogel P, Zoete V, Michielin O (2015). Challenges in the Discovery of Indoleamine 2,3-Dioxygenase 1 (IDO1) Inhibitors. J. Med. Chem..

[9] Munn DH, Mellor AL (2007). Indoleamine 2,3-dioxygenase and tumor-induced tolerance. J. Clin. Investig..

[10] van Baren, N. & Van den Eynde, B. J. Tryptophan-degrading enzymes in tumoral immune resistance. *Front. Immunol*. **6** (2015). 10.3389/fimmu.2015.00034 PMC4315104 25691885

[11] Hou DY (2007). Inhibition of indoleamine 2,3-dioxygenase in dendritic cells by stereoisomers of 1-methyl-tryptophan correlates with antitumor responses. Cancer Res..

[12] Muller AJ, DuHadaway JB, Donover PS, Sutanto-Ward E, Prendergast GC (2005). Inhibition of indoleamine 2,3-dioxygenase, an immunoregulatory target of the cancer suppression gene Bin1, potentiates cancer chemotherapy. Nat. Med..

[13] Koblish HK (2010). Hydroxyamidine Inhibitors of Indoleamine-2,3-dioxygenase Potently Suppress Systemic Tryptophan Catabolism and the Growth of IDO-Expressing Tumors. Mol. Cancer Ther..

[14] Smith C (2012). IDO Is a Nodal Pathogenic Driver of Lung Cancer and Metastasis Development. Cancer Discov..

[15] Favre, D. *et al*. Tryptophan Catabolism by Indoleamine 2,3-Dioxygenase 1 Alters the Balance of T(H)17 to Regulatory T Cells in HIV Disease. *Sci. Transl. Med*. **2** (2010). 10.1126/scitranslmed.3000632 PMC3034445 20484731

[16] Yu D (2015). The IDO Inhibitor Coptisine Ameliorates Cognitive Impairment in a Mouse Model of Alzheimer’s Disease. J. Alzheimers Dis..

[17] Potula R (2005). Inhibition of indoleamine 2,3-dioxygenase (IDO) enhances elimination of virus-infected macrophages in an animal model of HIV-1 encephalitis. Blood.

[18] Huang Q (2011). Structure-activity relationship and enzyme kinetic studies on 4-aryl-1H-1,2, 3-triazoles as indoleamine 2,3-dioxygenase (IDO) inhibitors. Eur. J. Med. Chem..

[19] Rohrig UF (2012). Rational design of 4-aryl-1,2,3-triazoles for indoleamine 2,3-dioxygenase 1 inhibition. J. Med. Chem..

[20] Panda S, Maity P, Manna D (2017). Transition Metal, Azide, and Oxidant-Free Homo- and Heterocoupling of Ambiphilic Tosylhydrazones to the Regioselective Triazoles and Pyrazoles. Org. Lett..

[21] Madadi NR (2015). Synthesis and biological evaluation of novel 4,5-disubstituted 2H-1,2,3-triazoles as cis-constrained analogues of combretastatin A-4. Eur. J. Med. Chem..

[22] Ponpandian T, Muthusubramanian S (2012). Tandem Knoevenagel-[3+2] cycloaddition-elimination reactions: one-pot synthesis of 4,5-disubstituted 1,2,3-(NH)-triazoles. Tett. Lett..

[23] Buckman, B. O., Nicholas, J. B., Emayan, K. & Seiwert, S. D. Lysophosphatidic acid receptor antagonists. WO 2013025733 A1 (2013).

[24] Glossop, P. A. & Lane, C. A. L. Novel compounds active as muscarinic receptor antagonists. WO 2010007561 A1 (2010).

[25] Yue EW (2017). INCB24360 (Epacadostat), a Highly Potent and Selective Indoleamine-2,3-dioxygenase 1 (ID01) Inhibitor for Immunooncology. Acs Med. Chem. Lett..

[26] Malachowski WP (2016). O-alkylhydroxylamines as rationally-designed mechanism-based inhibitors of indoleamine 2,3-dioxygenase-1. Eur. J. Med. Chem..

[27] Panda S, Roy A, Deka SJ, Trivedi V, Manna D (2016). Fused Heterocyclic Compounds as Potent Indoleamine-2,3-dioxygenase 1 Inhibitors. Acs Med. Chem. Lett..

[28] Paul S (2017). Synthesis and evaluation of oxindoles as promising inhibitors of the immunosuppressive enzyme indoleamine 2,3-dioxygenase 1. Medchemcomm.

[29] Paul S (2016). Nitrobenzofurazan derivatives of N ‘-hydroxyamidines as potent inhibitors of indoleamine-2,3-dioxygenase 1. Eur. J. Med. Chem..

[30] Pradhan N (2017). Identification of Substituted 1H-Indazoles as Potent Inhibitors for Immunosuppressive Enzyme Indoleamine 2,3-Dioxygenase 1. Chemistryselect.

[31] Yue EW (2009). Discovery of Potent Competitive Inhibitors of Indoleamine 2,3-Dioxygenase with *in Vivo* Pharmacodynamic Activity and Efficacy in a Mouse Melanoma Model. J. Med. Chem..

[32] Matsuno K (2010). S-Benzylisothiourea derivatives as small-molecule inhibitors of indoleamine-2,3-dioxygenase. Bioorganic & Medicinal Chemistry Letters.

[33] Rohrig UF (2010). Rational Design of Indoleamine 2,3-Dioxygenase Inhibitors. J. Med. Chem..

[34] Yang SS (2013). Discovery of Tryptanthrin Derivatives as Potent Inhibitors of Indoleamine 2,3-Dioxygenase with Therapeutic Activity in Lewis Lung Cancer (LLC) Tumor-Bearing Mice. J. Med. Chem..

[35] Gao DD, Li YX (2017). Identification and preliminary structure-activity relationships of 1-Indanone derivatives as novel indoleamine-2,3-dioxygenase 1 (IDO1) inhibitors. Bioorg. Med. Chem..

[36] Tojo S (2014). Crystal Structures and Structure Activity Relationships of Imidazothiazole Derivatives as IDO1 Inhibitors. Acs Med. Chem. Lett..

[37] Terentis AC (2010). The Selenazal Drug Ebselen Potently Inhibits Indoleamine 2,3-Dioxygenase by Targeting Enzyme Cysteine Residues. Biochemistry.

[38] Littlejohn TK, Takikawa O, Truscott RJW, Walker MJ (2003). Asp(274) and His(346) are essential for heme binding and catalytic function of human indoleamine 2,3-dioxygenase. J. Biol. Chem..

[39] Nayar S, Mir A, Ashok A, Guha A, Sharma V (2010). Bovine Serum Albumin Binding and Drug Delivery Studies with PVA-Ferrofluid. J. Bionic. Eng..

[40] Travers MT, Gow IF, Barber MC, Thomson J, Shennan DB (2004). Indoleamine 2,3-dioxygenase activity and L-tryptophan transport in human breast cancer cells. Biochim. Biophys. Acta, Biomembr..

[41] Beatty GL, Gladney WL (2015). Immune Escape Mechanisms as a Guide for Cancer Immunotherapy. Clinic. Cancer Res..

[42] Prendergast GC, Malachowski WP, DuHadaway JB, Muller AJ (2017). Discovery of IDO1 Inhibitors: From Bench to Bedside. Cancer Res..

[43] Munn DH (1998). Prevention of allogeneic fetal rejection by tryptophan catabolism. Science.

[44] Fox JM (2013). Inhibition of indoleamine 2,3-dioxygenase enhances the T-cell response to influenza virus infection. J. Gen. Virol..

[45] Zhang N, Bevan MJ (2011). CD8(+) T cells: foot soldiers of the immune system. Immunity.

[46] Srivastava M (2014). Sapodilla plum (Achras sapota) induces apoptosis in cancer cell lines and inhibits tumor progression in mice. Sci. Rep..

[47] Saha A (2019). Fatty-Amine-Conjugated Cationic Bovine Serum Albumin Nanoparticles for Target-Specific Hydrophobic Drug Delivery. ACS Appl. Nano Mater..

[48] Sharma S (2013). A novel DNA intercalator, 8-methoxy pyrimido[4′,5′:4,5]thieno (2,3-b)quinoline-4(3H)-one induces apoptosis in cancer cells, inhibits the tumor progression and enhances lifespan in mice with tumor. Mol. Carcinogen.

[49] Maimela NR, Liu S, Zhang Y (2019). Fates of CD8+ T cells in Tumor Microenvironment. Comput. struct. biotech. j..

[50] Austin CJD (2004). Optimised expression and purification of recombinant human indoleamine 2,3-dioxygenase. Protein Expres. Purif..

[51] Takikawa O, Kuroiwa T, Yamazaki F, Kido R (1988). Mechanism of Interferon-Gamma Action - Characterization of Indoleamine 2,3-Dioxygenase in Cultured Human-Cells Induced by Interferon-Gamma and Evaluation of the Enzyme-Mediated Tryptophan Degradation in Its Anticellular Activity. J. Biol. Chem..

[52] Gorai S (2015). Inhibition of phosphatidylinositol-3,4,5-trisphosphate binding to the AKT pleckstrin homology domain by 4-amino-1,2,5-oxadiazole derivatives. Medchemcomm.

[53] Talukdar D, Panda S, Borah R, Manna D (2014). Membrane Interaction and Protein Kinase C-C1 Domain Binding Properties of 4-Hydroxy-3-(hydroxymethyl) Phenyl Ester Analogues. J. Phys. Chem. B.

[54] Takikawa O, Yoshida R, Kido R, Hayaishi O (1986). Tryptophan Degradation in Mice Initiated by Indoleamine 2,3-Dioxygenase. J. Biol. Chem..

[55] Johnston L, Harding SA, La Flamme AC (2015). Comparing methods for *ex vivo* characterization of human monocyte phenotypes and *in vitro* responses. Immunobiology.

[56] Crawford TQ, Jalbert E, Ndhlovu LC, Barbour JD (2014). Concomitant Evaluation of PMA plus Ionomycin-Induced Kinase Phosphorylation and Cytokine Production in T Cell Subsets by Flow Cytometry. Cytom. Part A.

[57] Chakraborty K, Chatterjee S, Bhattacharyya A (2018). Impact of Treg on other T cell subsets in progression of fibrosis in experimental lung fibrosis. Tissue Cell.

[58] Aziz, M. A. *et al*. Discovery of Potent VEGFR-2 Inhibitors based on Furopyrimidine and Thienopyrimidne Scaffolds as Cancer Targeting Agents. *Sci. Rep*. **6** (2016). 10.1038/srep24460 PMC4832243 27080011

