# Supplementary material for: 4,5-Disubstituted 1,2,3-triazoles: Effective Inhibition of Indoleamine 2,3-Dioxygenase 1 Enzyme Regulates T cell Activity and Mitigates Tumor Growth
Source: Sci Rep. 2019 Dec 5;9:18455. doi: 10.1038/s41598-019-54963-9 (PMC6895048; doi:10.1038/s41598-019-54963-9)
Supplement: Supplementary file 1 — Supplementary information [file 41598_2019_54963_MOESM1_ESM.pdf]

4,5-Disubstituted 1,2,3-triazoles: Effective Inhibition of Indoleamine 2,3-Dioxygenase 1 Enzyme Regulates T cell Activity and Mitigates Tumor Growth

Subhankar Panda,[‡,a] Nirmalya Pradhan,[‡,a] Soumya Chatterjee,[b] Sudhir Morla,[c] Abhishek Saha,[a] Ashalata Roy,[a] Sachin Kumar,[c] Arindam Bhattacharyya*[,b] and Debasis Manna*[,a]

[a]Department of Chemistry, Indian Institute of Technology Guwahati, Guwahati 781039, Assam, India

[b]Department of Zoology, University of Calcutta, Kolkata 700019, West Bengal, India

[c]Department of Bioscience and Bioengineering, Indian Institute of Technology Guwahati, Guwahati 781039, Assam, India

E-mail: dmanna@iitg.ernet.in

Supporting Information

TABLE OF CONTENTS

Sl. No	Content	Page
1.	Characterization of the synthesized compounds	S3-19
2.	Purification of the compounds by HPLC analysis	S19
3.	HPLC based IDO1 inhibition assays of the selected compounds	S20
4.	Characteristic peaks from the UV-Vis measurements of IDO1 enzyme and potent compounds	S20
5.	SPR Analysis	S21
6.	IDO1 binding affinity of various compounds determined from equilibrium SPR analysis	S22
7.	Docking parameters for the interaction of IDO1 enzyme with 1,2,3-triazoles	S22
8.	Circular dichroism analysis of IDO1 enzyme in the presence of selected 1,2,3-triazole compounds	S23
9.	Mode of IDO1 enzyme inhibition	S24
10.	Enzyme kinetics parameters of the IDO1 enzyme in the presence of selected 1,2,3-triazoles	S24
11.	Inhibitory activity of the 1,2,3-triazoles against purified human TDO enzyme	S25
12.	HPLC based TDO inhibition assays of the selected compounds	S26
13.	Analysis of the serum binding ability of the potent compounds	S27
14.	Measurement of cytotoxicity of the potent compounds	S28-29
15.	EC_{50} values of the selected compounds in MDA-MB-231 cells	S29
16.	T cell activity studies	S30-32
17.	Flowcytometry analysis for % population of CD8 T cell in *in vivo* solid tumor	S33
18.	^{1}H and ^{13}C NMR spectra of synthesized compounds	S34-81
19.	References	S82

Characterization of the synthesized compounds:

4,5-Diphenyl-2*H*-1,2,3-triazole (1a)[1] — To a stirring solution of (E)-*N'*-benzylidene-4-methylbenzenesulfonohydrazide (482 mg, 1.8 mmol) in DMF (5 mL) was added Cs_2CO_3 (878 mg, 2.7 mmol) and allowed to reflux at 100 °C. The progress of the reaction was monitored by TLC technique. Then the reaction mixture was cooled down to room temperature and diluted with cold water and ethyl acetate. The organic layer was extracted and washed with brine and dried over anhydrous Na_2SO_4. The organic solvent was removed under reduced pressure. Purification by column chromatography using EtOAc/Hexane (10-40%) solvent gradient produced the target compound 127 mg (65% yield, time = 8 h) of **1a** as white solid. Mp: 130-132 °C (in lit[2]: 130-131 °C); ^{1}H NMR (600 MHz, $CDCl_3$) δ_{ppm} 11.98 (s, 1H), 7.55 – 7.53 (m, 4H), 7.37 – 7.33 (m, 6H).; ^{13}C NMR (100 MHz, $CDCl_3$) δ_{ppm} 143.1, 130.5, 128.9, 128.8, 128.5.; FT-IR (KBr) 3412, 3045, 1530, 1426, 1120, 1022 cm^{-1}; HRMS (ESI) calcd. for $C_{14}H_{11}N_3$ $[M + H]^+$: 222.0953, found: 222.0950.

4,5-Bis(4-Methylphenyl)-2*H*-1,2,3-triazole (1b) — Synthesized as compound **1a** using (E)-4-methyl-*N'*-(4-methylbenzylidene) benzenesulfonohydrazide (360 mg, 1.26 mmol) and Cs_2CO_3 (612 mg, 1.89 mmol). The reaction provided 101 mg (65% yield, time = 10 h) of **1b** as white solid; Mp: 156-158 °C ((in lit.[2]: 156-157 °C); ^{1}H NMR (600 MHz, $CDCl_3$) δ_{ppm} 7.42 (d, J = 8.0 Hz, 4H), 7.15 (d, J = 7.7 Hz, 4H), 2.35 (s, 6H).; ^{13}C NMR (151 MHz, $CDCl_3$) δ_{ppm} 142.8, 138.6, 129.6, 128.3, 127.6, 21.5.; FT-IR (KBr) 3408, 3021, 2941, 1513, 1421, 1119, 1021 cm^{-1}; HRMS (ESI) calcd. for $C_{16}H_{15}N_3$ $[M + H]^+$: 250.1266, found: 250.1668.

4,5-Bis(3-fluorophenyl)-2*H*-1,2,3-triazole (1c) — Synthesized as compound **1a** using (E)-N'-(3-fluorobenzylidene)-4-methylbenzenesulfonohydrazide (452 mg, 1.55 mmol) and Cs_2CO_3 (755 mg, 2.32 mmol). The reaction provided 165 mg (83% yield, time = 4 h) of **1c** as white gummy solid; ^{1}H NMR (600 MHz, $CDCl_3$) δ_{ppm} 7.30 – 7.24 (m, 4H), 7.21 – 7.19 (m, 2H), 7.03 – 7.00 (m, 2H).; ^{13}C NMR (100 MHz, $CDCl_3$) δ_{ppm} 164.3, 161.8, 142.5, 132.1 (d, J = 8.4 Hz), 130.7 (d, J = 8.4 Hz), 124.2 (d, J = 3.0 Hz), 116.2, 116.0, 115.6, 115.4.; FT-IR (KBr) 3411, 3029, 2940, 1517, 1413, 1109, 1023 cm^{-1}; HRMS (ESI) calcd. for $C_{14}H_9F_2N_3$ $[M + H]^+$: 258.0798, found: 258.0798.

4,5-Bis(3-chlorophenyl)-2*H*-1,2,3-triazole (1d) — Synthesized as compound **1a** using ((E)-*N'*-(3-chlorobenzylidene)-4-methylbenzenesulfonohydrazide (290 mg, 0.95 mmol) and Cs_2CO_3 (463 mg, 1.43 mmol). The reaction provided 118 mg (87% yield, time = 4 h) of **1d** as white solid; Mp: 162-163 °C; ^{1}H NMR (400 MHz, $CDCl_3$) δ_{ppm} 7.57 (s, 2H), 7.37 – 7.33 (m, 4H), 7.30 – 7.26 (m, 2H); ^{13}C NMR (151 MHz, $CDCl_3$) δ_{ppm} 142.5, 135.0, 131.9, 130.2, 129.2, 128.5, 126.6; FT-IR (KBr) 3422, 3045, 1531, 1426, 1124, 1021 cm^{-1}; HRMS (ESI) calcd. for $C_{14}H_9Cl_2N_3$ $[M + H]^+$: 290.0174, found: 290.0172.

4,5-Bis(3-bromophenyl)-2*H*-1,2,3-triazole (1e) — Synthesized as compound **1a** using (E)-N'-(3-bromobenzylidene)-4-methylbenzenesulfonohydrazide (342 mg, 0.96 mmol) and Cs_2CO_3 (472 mg, 1.45 mmol). The reaction provided 154 mg (84% yield, time = 4 h) of **1e** as pale yellow gummy solid; ^{1}H NMR (600 MHz, $CDCl_3$) δ_{ppm} 7.72 (s, 2H), 7.49 – 7.47 (m, 2H), 7.38 (d, J = 7.8 Hz, 2H), 7.19 (t, J = 7.9 Hz, 2H).; ^{13}C NMR (100 MHz, $CDCl_3$) δ_{ppm} 141.8, 132.1, 131.8, 131.3, 130.5, 127.0, 123.1; FT-IR (KBr) 3429, 3051, 1511, 1420, 1121, 1022 cm^{-1}; HRMS (ESI) calcd. for $C_{14}H_9Br_2N_3$ $[M + H]^+$: 379.9163, found: 379.9163.

4,5-Bis(4-fluorophenyl)-2*H*-1,2,3-triazole (1f) — Synthesized as compound **1a** using ((E)-*N'*-(4-fluorobenzylidene)-4-methylbenzenesulfonohydrazide (248 mg, 0.84 mmol) and Cs_2CO_3 (410 mg, 1.26 mmol). The reaction provided 88 mg (83% yield, time = 4 h) of **1f** as yellow solid; Mp: 144-146 °C; ^{1}H NMR (600 MHz, $CDCl_3$) δ_{ppm} 7.51-7.48 (m, 4H), 7.08-7.06 (m, 4H).; ^{13}C NMR (151 MHz, $CDCl_3$) δ_{ppm} 164.0, 162.4, 142.4, 130.3 (d, J = 8.1 Hz), 126.3, 116.2, 116.0; FT-IR (KBr) 3417, 3049, 1531, 1421, 1115, 1028 cm^{-1}; HRMS (ESI) calcd. for $C_{14}H_9F_2N_3$ $[M + H]^+$: 258.0765, found: 258.0765.

4,5-Bis(4-chlorophenyl)-2*H*-1,2,3-triazole (1g) — Synthesized as compound **1a** using ((E)-*N'*-(4-chlorobenzylidene)-4-methylbenzenesulfonohydrazide (260 mg, 0.84 mmol) and Cs_2CO_3 (410 mg, 1.26 mmol). The reaction provided 108 mg (90% yield, time = 4 h) of **1g** as white solid; Mp: 187-189 °C (in lit.[3]: 187-188 °C); ^{1}H NMR (600 MHz, $CDCl_3$) δ_{ppm} 7.44 (d, J = 8.2 Hz, 4H), 7.33 (d, J = 8.3 Hz, 4H).; ^{13}C NMR (151 MHz, $CDCl_3$) δ_{ppm} 142.5, 135.1, 129.7, 129.3, 128.7; FT-IR (KBr) 3421, 3060, 1530,1378, 1259, 1218, 1120, 1022 cm^{-1}; HRMS (ESI) calcd. for $C_{14}H_9Cl_2N_3$ $[M + H]^+$: 290.0174, found: 290.0174.

4,5-Bis(4-bromophenyl)-2*H*-1,2,3-triazole (1h) — Synthesized as compound **1a** using ((E)-*N'*-(4-bromobenzylidene)-4-methylbenzenesulfonohydrazide (280 mg, 0.80 mmol) and Cs_2CO_3 (390 mg, 1.2 mmol). The reaction provided 118 mg (78% yield, time = 4 h) of **1h** as yellow solid; Mp: 184-186 °C; ^{1}H NMR (600 MHz, $CDCl_3$) δ_{ppm} 11.72 (s, 1H), 7.51 (d, *J* = 8.4 Hz, 4H), 7.40 (d, *J* = 8.4 Hz, 4H).; ^{13}C NMR (100 MHz, $CDCl_3$) δ_{ppm} 132.3, 130.1, 129.4, 123.4; FT-IR (KBr) 3431, 3043, 1533, 1435, 1117, 1016 cm^{-1}; HRMS (ESI) calcd. for $C_{14}H_9Br_2N_3$ $[M + H]^+$: 379.9163, found: 379.9163.

4,5-Bis(3,4-difluorophenyl)-2*H*-1,2,3-triazole (1i) — Synthesized as compound **1a** using ((E)-*N'*-(3,4-difluorobenzylidene)-4-methylbenzenesulfonohydrazide (256 mg, 0.82 mmol) and Cs_2CO_3 (402 mg, 1.24 mmol). The reaction provided 90 mg (75% yield, time = 6 h) of **1i** as yellow solid; Mp: 144-146 °C; ^{1}H NMR (600 MHz, $CDCl_3$) δ_{ppm} 7.39-7.36 (m, 2H), 7.24-7.23 (m, 2H), 7.20-7.16 (m, 2H); ^{13}C NMR (126 MHz, $CDCl_3$) δ_{ppm} 151.9 (dd, *J* = 32.6, 13.5 Hz), 150.0 (d, *J* = 9.4 Hz), 149.7 (d, *J* = 17.5 Hz), 142.3, 127.2 (d, *J* = 4.0 Hz), 124.8 (dd, *J* = 6.3, 3.7 Hz), 118.1 (d, *J* = 17.5 Hz), 117.7 (d, *J* = 18.5 Hz).; FT-IR (KBr) 3416, 3049, 1513, 1426, 1111, 1012 cm^{-1}; HRMS (ESI) calcd. for $C_{14}H_7F_4N_3$ $[M + H]^+$: 294.0576, found: 294.0577.

4,5-Bis(3,4-dichlorophenyl)-2*H*-1,2,3-triazole (1j) — Synthesized as compound **1a** using ((E)-*N'*-(3,4-dichlorobenzylidene)-4-methylbenzenesulfonohydrazide (290 mg, 0.84 mmol) and Cs_2CO_3 (408 mg, 1.26 mmol). The reaction provided 116 mg (78% yield, time = 5 h) of **1j** as white solid; Mp: 170-172 °C; ^{1}H NMR (600 MHz, $CDCl_3$) δ_{ppm} 7.69 (s, 2H), 7.45-7.44 (m, 2H), 7.31-7.29 (m, 2H); ^{13}C NMR (75 MHz, $CDCl_3$) δ_{ppm} 142.2, 133.6 (d, *J* = 3.9 Hz), 131.1, 130.2 (d, *J* = 15.2 Hz), 127.6; FT-IR (KBr) 3402, 3055, 1540, 1436, 1110, 1032 cm^{-1}; HRMS (ESI) calcd. for $C_{14}H_7Cl_4N_3$ $[M + H]^+$: 359.9394, found: 359.9395.

4,5-Bis(3-chloro, 4-fluorophenyl)-2*H*-1,2,3-triazole (1k) — Synthesized as compound **1a** using ((E)-*N'*-(3-chloro, 4-fluorobenzylidene)-4-methylbenzenesulfonohydrazide (375 mg, 1.14 mmol) and Cs_2CO_3 (555 mg1.71 mmol). The reaction provided 138 mg (75% yield, time = 6 h) of **1k** as yellow solid; Mp: 156-158 °C; ^{1}H NMR (600 MHz, $CDCl_3$) δ_{ppm} 7.64 – 7.63 (m, 2H), 7.36 – 7.33 (m, 2H), 7.17 – 7.14 (m, 2H); ^{13}C NMR (151 MHz, $CDCl_3$) δ_{ppm} 159.5, 157.8, 142.0, 130.7,

128.3 (d, J = 7.5 Hz), 127.3, 122.0 (d, J = 18.1 Hz), 117.4, 117.2; FT-IR (KBr) 3414, 3043, 1539, 1421, 1127, 1025 cm^{-1}; HRMS (ESI) calcd. for $C_{14}H_7Cl_2F_2N_3$ $[M + H]^+$: 326.0057, found: 326.0050.

4,5-Bis(3-Bromo, 4-fluorophenyl)-2*H*-1,2,3-triazole (1l) — Synthesized as compound **1a**, using ((E)-*N'*-(3-bromo, 4-fluorobenzylidene)-4-methylbenzenesulfonohydrazide (290 mg, 0.78 mmol) and Cs_2CO_3 (382 mg, 1.18 mmol). The reaction provided 112 mg (70% yield, time = 6 h) of **1l** as yellow solid; Mp: 177-179 °C; ^1H NMR (600 MHz, $CDCl_3$) δ_{ppm} 7.81 – 7.79 (m, 2H), 7.39 – 7.37 (m, 2H), 7.13 – 7.10 (m, 2H).; ^{13}C NMR (100 MHz, $CDCl_3$) δ_{ppm} 160.9, 158.4, 142.2, 133.6, 129.1 (d, J = 7.5 Hz), 127.8 (d, J = 3.9 Hz), 117.2, 117.0, 110.1, 109.9. FT-IR (KBr) 3412, 3045, 1530, 1426, 1120, 1022 cm^{-1}; HRMS (ESI) calcd. for $C_{14}H_7Br_2F_2N_3$ $[M + H]^+$: 415.8975, found: 415.8977.

Ethyl 5-phenyl-2*H*-1,2,3-triazole-4-carboxylate (2a)[4,5] — To a stirring solution of benzaldehyde (180 μL, 1.7 mmol) and ethyl 2-cyanoacetate (176 μL, 1.87 mmol) in DMF (1.5 mL) was added NH_4Cl (315 mg, 5.95 mmol) and stirred for 10 minute. Then, NaN_3 (332 mg, 5.1 mmol) was added to the resultant reaction mixture and allowed to stir under reflux at 70 °C for 6 h. The progress of the reaction was monitored by TLC technique. After completion of the reaction, the reaction mixture was cooled down to room temperature and neutralized by 10% dilute HCl solution. Then, the reaction mixture was diluted with cold water and ethyl acetate. The organic layer was extracted and washed with brine and dried over anhydrous Na_2SO_4. The organic solvent was removed under reduced pressure. Purification by column chromatography using EtOAc/Hexane (15-40%) solvent gradient produced the target compound 223 mg (63% yield, time = 6 h) of **2a** as white solid, Mp: 107 -109 °C (in lit[5]: 109-111 °C) ; ^1H NMR (600 MHz, $CDCl_3$) δ_{ppm} 7.78 – 7.76 (m, 2H), 7.40 – 7.39 (m, 3H), 4.36-4.32 (m, 2H), 1.28-1.26 (m, 3H); ^{13}C NMR (100 MHz, $CDCl_3$) δ_{ppm} 161.3, 146.4, 134.2, 129.9, 129.4, 128.5, 127.9, 61.9, 14.2; FT-IR (KBr) 3072, 2980, 1736, 1520, 1434, 1117 cm^{-1}; HRMS (ESI) calcd. for $C_{11}H_{11}N_3O_2$ $[M + H]^+$: 218.0885, found: 218.0885.

Ethyl 5-(p-tolyl)-2*H*-1,2,3-triazole-4-carboxylate (2b) — Synthesized as compound **2a** using 4-methylbenzaldehyde (170 μL, 1.44 mmol), ethyl 2-cyanoacetate (160 μL, 1.58 mmol), NH_4Cl

(267 mg, 5.04 mmol) and NaN_3 (281 mg, 4.32 mmol). The reaction provided 185 mg (60% yield, time = 6 h) of **2b** as white solid, Mp: 139-141 °C; ^{1}H NMR (600 MHz, $CDCl_3$) δ_{ppm} 7.67 (d, J = 12.0 Hz, 2H), 7.20 (d, J = 6 Hz, 2H), 4.34 (q, J = 7.1 Hz, 2H), 2.35 (s, 3H), 1.28 (t, J = 7.1 Hz, 3H); ^{13}C NMR (100 MHz, $CDCl_3$) δ_{ppm} 161.3, 140.1, 129.3 (d, J = 2.7 Hz), 124.8, 61.8, 21.6, 14.3; FT-IR (KBr) 3045, 2983, 1740, 1521, 1424, 1107 cm^{-1}; HRMS (ESI) calcd. for $C_{12}H_{13}N_3O_2$ $[M + H]^+$: 232.1041, found: 232.1041.

Ethyl 5-(3-fluorophenyl)-2*H*-1,2,3-triazole-4-carboxylate (2c) — Synthesized as compound **2a** using 3-fluorobenzaldehyde (165 µL, 1.56 mmol), ethyl 2-cyanoacetate (182 µL, 1.71 mmol), NH_4Cl (289 mg, 5.46 mmol) and NaN_3 (304 mg, 4.68 mmol). The reaction provided 263 mg (72% yield, time = 5 h) of **2c** as white solid Mp: 132-134 °C; ^{1}H NMR (600 MHz, $CDCl_3$) δ_{ppm} 7.64 (d, J = 7.7 Hz, 1H), 7.60 (d, J = 9.8 Hz, 1H), 7.39 (dd, J = 14.0, 8.0 Hz, 1H), 7.14 – 7.11 (m, 1H), 4.42 (q, J = 7.1 Hz, 2H), 1.35 (t, J = 7.1 Hz, 3H).; ^{13}C NMR (100 MHz, $CDCl_3$) δ_{ppm} 163.9, 161.5, 161.0, 130.1 (d, J = 8.3 Hz), 125.2 (d, J = 3.0 Hz), 116.7 (dd, J = 22.3, 7.1 Hz), 62.2, 14.3; FT-IR (KBr) 3082, 2975, 1733, 1521, 1435, 1127 cm^{-1}; HRMS (ESI) calcd. for $C_{11}H_{10}FN_3O_2$ $[M + H]^+$: 236.0791, found: 236.0791.

Ethyl 5-(3-chlorophenyl)-2*H*-1,2,3-triazole-4-carboxylate (2d) — Synthesized as compound **2a** using 3-chlorobenzaldehyde (130 µL, 1.15 mmol), ethyl 2-cyanoacetate (135 µL, 1.58 mmol), NH_4Cl (213 mg, 4.03 mmol) and NaN_3 (224 mg, 3.45 mmol). The reaction provided 219 mg (76% yield, time = 4 h) of **2d** as white solid; Mp: 130-132 °C; ^{1}H NMR (600 MHz, $CDCl_3$) δ_{ppm} 7.87 (s, 1H), 7.75 (d, J = 7.4 Hz, 1H), 7.41 – 7.36 (m, 2H), 4.42 (q, J = 7.1 Hz, 2H), 1.37 (t, J = 7.1 Hz, 3H).; ^{13}C NMR (126 MHz, $CDCl_3$) δ_{ppm} 161.1, 146.3, 134.4 (d, J = 6.8 Hz), 130.3, 129.8, 129.7, 129.6, 127.7, 62.2, 14.2; FT-IR (KBr) 3062, 2978, 1738, 1523, 1424, 1119 cm^{-1}; HRMS (ESI) calcd. for $C_{11}H_{10}ClN_3O_2$ $[M + H]^+$: 253.0432, found: 253.0432.

Ethyl 5-(3-bromophenyl)-2*H*-1,2,3-triazole-4-carboxylate (2e) — Synthesized as compound **2a** using 3-bromobenzaldehyde (100 µL, 0.85 mmol), ethyl 2-cyanoacetate (100 µL, 0.94 mmol), NH_4Cl (158 mg, 2.98 mmol) and NaN_3 (166 mg, 2.55 mmol). The reaction provided 176 mg (70% yield, time = 5 h) of **2e** as pale yellow gummy solid; ^{1}H NMR (600 MHz, $CDCl_3$) δ_{ppm} 8.02 (s, 1H), 7.80 (d, J = 7.7 Hz, 1H), 7.55 (d, J = 7.9 Hz, 1H), 7.31 (t, J = 7.9 Hz, 1H), 4.41 (q, J =

7.1 Hz, 2H), 1.37 (t, *J* = 7.1 Hz, 3H).; ^{13}C NMR (100 MHz, $CDCl_3$) δ_{ppm} 161.1, 132.7, 132.4, 130.7, 130.0, 128.1, 122.4, 62.2, 14.3; FT-IR (KBr) 3065, 2981, 1735, 1521, 1437, 1115 cm^{-1}; HRMS (ESI) calcd. for $C_{11}H_{10}BrN_3O_2$ [M + H]$^+$: 296.0029, found: 296.0029.

Ethyl 5-(4-fluorophenyl)-2*H*-1,2,3-triazole-4-carboxylate (2f) — Synthesized as compound **2a** using 4-fluorobenzaldehyde (130 µL, 1.21 mmol), ethyl 2-cyanoacetate (142 µL, 1.33 mmol), NH_4Cl (224 mg, 4.24 mmol) and NaN_3 (236 mg, 3.63 mmol). The reaction provided 201 mg (71% yield, time = 4 h) of **2f** as red solid; Mp: 130 - 132 °C; ^{1}H NMR (600 MHz, $CDCl_3$) δ_{ppm} 7.85 – 7.81 (m, 2H), 7.12 – 7.06 (m, 2H), 4.41-4.37 (m, 2H), 1.34-1.31 (m, 3H); ^{13}C NMR (100 MHz, $CDCl_3$) δ_{ppm} 165.0, 162.5, 161.1, 141.6, 131.5 (d, *J* = 8.5 Hz), 131.0, 124.9, 119.1, 115.7, 115.5, 62.1, 14.3; FT-IR (KBr) 3071, 2986, 1743, 1523, 1431, 1121 cm^{-1}; HRMS (ESI) calcd. for $C_{11}H_{10}FN_3O_2$ [M + H]$^+$: 236.0791, found: 236.0791.

Ethyl 5-(4-chlorophenyl)-2*H*-1,2,3-triazole-4-carboxylate (2g) — Synthesized as compound **2a** using 4-chlorobenzaldehyde (125 mg, 0.89 mmol), ethyl 2-cyanoacetate (103 µL, 0.97 mmol), NH_4Cl (165 mg, 3.12 mmol) and NaN_3 (174 mg, 2.67 mmol). The reaction provided 179 mg (80% yield, time = 4 h) of **2g** as white solid; Mp: 165 - 167 °C (in lit[6]. 164-167 °C); ^{1}H NMR (600 MHz, $CDCl_3$) δ_{ppm} 7.82 (d, *J* = 8.5 Hz, 2H), 7.42 (d, *J* = 8.5 Hz, 2H), 4.41 (q, *J* = 7.1 Hz, 2H), 1.37 (t, *J* = 7.1 Hz, 3H).; ^{13}C NMR (100 MHz, $CDCl_3$) δ_{ppm} 161.2, 146.3, 136.0, 134.3, 130.8, 130.7, 128.8, 126.7, 62.2, 14.3; FT-IR (KBr) 3074, 2987, 1744, 1512, 1429, 1120 cm^{-1}; HRMS (ESI) calcd. for $C_{11}H_{10}ClN_3O_2$ [M + H]$^+$: 253.0432, found: 253.0432.

Ethyl 5-(4-bromophenyl)-2*H*-1,2,3-triazole-4-carboxylate (2h) — Synthesized as compound **2a** using 4-bromobenzaldehyde (120 mg, 0.65 mmol), ethyl 2-cyanoacetate (76 µL, 0.72 mmol), NH_4Cl (121 mg, 2.28 mmol) and NaN_3 (127 mg, 1.95 mmol). The reaction provided 130 mg (68% yield, time = 6 h) of **2h** as pale yellow solid; Mp: 180-182 °C; ^{1}H NMR (600 MHz, $CDCl_3$) δ_{ppm} 7.75 (d, *J* = 8.2 Hz, 2H), 7.58 (d, *J* = 8.5 Hz, 2H), 4.41 (q, *J* = 7.1 Hz, 2H), 1.37 (t, *J* = 7.1 Hz, 3H)·; ^{13}C NMR (100 MHz, DMSO-d_6) δ_{ppm} 160.9, 131.2 (d, *J* = 10.1 Hz), 122.8, 60.9, 14.0; FT-IR (KBr) 3071, 2977, 1747, 1511, 1437, 1123 cm^{-1}; HRMS (ESI) calcd. for $C_{11}H_{10}BrN_3O_2$ [M + H]$^+$: 296.0029, found: 296.0030.

Ethyl 5-(3,4-difluorophenyl)-2*H*-1,2,3-triazole-4-carboxylate (2i) — Synthesized as compound **2a** using 3,4-difluorobenzaldehyde (140 μL, 1.26 mmol), ethyl 2-cyanoacetate (148 μL, 1.39 mmol), NH_4Cl (233 mg, 4.41 mmol) and NaN_3 (246 mg, 3.78 mmol). The reaction provided 230 mg (71% yield, time = 5 h) of **2i** as pale yellow solid; Mp: 128-130 °C; ^1H NMR (600 MHz, $CDCl_3$) δ_{ppm} 7.77-7.73 (m, 2H), 7.14-7.11 (m, 1H), 4.43 (q, J = 7.1 Hz, 2H), 1.41-1.38 (m, 3H).; ^{13}C NMR (100 MHz, $CDCl_3$) δ_{ppm} 161.0, 155.7, 153.2, 145.9, 134.1, 129.3 (d, J = 11.1 Hz), 126.0 (d, J = 3.4 Hz), 121.0, 118.8 (d, J = 19.2 Hz), 118.0, 117.8, 117.4 (d, J = 17.9 Hz), 62.3, 14.2; FT-IR (KBr) 3073, 2975, 1739, 1517, 1431, 1119 cm^{-1}; HRMS (ESI) calcd. for $C_{11}H_9F_2N_3O_2$ $[M + H]^+$: 254.0696, found: 254.0691.

Ethyl 5-(3,4-dichlorophenyl)-2*H*-1,2,3-triazole-4-carboxylate (2j) — Synthesized as compound **2a** using 3,4-dichlorobenzaldehyde (115 mg, 0.66 mmol), ethyl 2-cyanoacetate (77 μL, 0.72 mmol), NH_4Cl (122 mg, 2.31 mmol) and NaN_3 (129 mg, 1.98 mmol). The reaction provided 154 mg (82% yield, time = 4 h) of **2j** as white solid; Mp: 140-142 °C; ^1H NMR (600 MHz, $CDCl_3$) δ_{ppm} 8.04 (s, 1H), 7.76 (d, J = 8.3 Hz, 1H), 7.51 (d, J = 8.4 Hz, 1H), 4.44 (q, J = 7.1 Hz, 2H), 1.39 (t, J = 7.1 Hz, 3H).; ^{13}C NMR (100 MHz, $CDCl_3$) δ_{ppm} 160.9, 146.4, 134.0, 132.7, 131.4, 130.5, 128.8, 62.5, 14.3; FT-IR (KBr) 3071, 2976, 1745, 1518, 1433, 1109 cm^{-1}; HRMS (ESI) calcd. for $C_{11}H_9Cl_2N_3O_2$ $[M + H]^+$: 286.0072, found: 286.0070.

Ethyl 5-(3-chloro-4-fluorophenyl)-2*H*-1,2,3-triazole-4-carboxylate (2k) — Synthesized as compound **2a** using 3-chloro-4-fluorobenzaldehyde (120 μL, 0.74 mmol), ethyl 2-cyanoacetate (86 μL, 0.81 mmol), NH_4Cl (137 mg, 2.59 mmol) and NaN_3 (144 mg, 2.22 mmol). The reaction provided 143 mg (72% yield, time = 5 h) of **2k** as pale off-white solid; Mp: 110-112 °C; ^1H NMR (600 MHz, $CDCl_3$) δ_{ppm} 7.99 (d, J = 1.7 Hz, 1H), 7.89 – 7.88 (m, 1H), 7.25 (s, 1H), 4.43 (q, J = 7.1 Hz, 2H), 1.39 (t, J = 7.1 Hz, 3H).; ^{13}C NMR (100 MHz, $CDCl_3$) δ_{ppm} 160.6, 146.7, 138.5, 131.7, 129.0, 126.7, 125.0, 119.6, 114.9, 62.3, 14.4; FT-IR (KBr) 3073, 2974, 1741, 1519, 1417, 1112 cm^{-1}; HRMS (ESI) calcd. for $C_{11}H_9ClFN_3O_2$ $[M + H]^+$: 270.0367, found: 270.0367.

Ethyl 5-(3-bromo-4-fluorophenyl)-2*H*-1,2,3-triazole-4-carboxylate (2l) — Synthesized as compound **2a** using 3-bromo-4-fluorobenzaldehyde (120 μL, 0.58 mmol), ethyl 2-cyanoacetate (68 μL, 0.64 mmol), NH_4Cl (108 mg, 2.03 mmol) and NaN_3 (113 mg, 1.74 mmol). The reaction

provided 124 mg (68% yield, time = 6 h) of **2l** as brown solid; Mp: 142-144 °C; ^{1}H NMR (600 MHz, $CDCl_3$) δ_{ppm} 8.16 (s, 1H), 7.95 – 7.93 (m, 1H), 7.26 – 7.25 (m, 1H), 4.47-4.44 (m, 2H), 1.42 – 1.39 (m, 3H).; ^{13}C NMR (151 MHz, $CDCl_3$) δ_{ppm} 160.8, 146.4, 140.0, 134.7, 129.7, 126.9, 119.2, 113.7, 62.3, 14.4; FT-IR (KBr) 3087, 2991, 1737, 1523, 1429, 1121 cm^{-1}; HRMS (ESI) calcd. for $C_{11}H_9BrFN_3O_2$ $[M + H]^+$: 313.9935, found: 313.9935.

5-phenyl-2*H*-1,2,3-triazole-4-carboxamide (3a)[7] **—** To a stirring solution of benzaldehyde (140 µL, 1.37 mmol) and 2-cyanoacetamide (126 mg, 1.51 mmol) in DMF (1.5 mL) was added NH_4Cl (254 mg, 4.80 mmol) and stirred for 10 minute. Then NaN_3 (267 mg, 4.11 mmol) was added to the resultant reaction mixture and allowed to stir under reflux at 70 °C for 5 h. The progress of the reaction was monitored by TLC technique. After completion the reaction, it was cooled down to room temperature and neutralized by 10% dilute HCl. The obtained solid precipitate was filtered, and the residue was washed with cold water. The solid residue was dried under vacuum to get the target compound 180 mg (70% yield, time = 5 h) of **3a** as pale yellow solid; Mp: 278 - 290 °C (in lit[7]: 277 – 278 °C); ^{1}H NMR (500 MHz, DMSO-d_6) δ_{ppm} 7.93 – 7.90 (m, 2H), 7.54 – 7.43 (m, 3H).; ^{13}C NMR (126 MHz, DMSO-d_6) δ_{ppm} 162.9, 137.7, 128.7, 128.2.; FT-IR (KBr) 3387, 3251, 2991, 1667, 1523, 1429, 1121 cm^{-1}; HRMS (ESI) calcd. for $C_9H_8N_4O$ $[M + H]^+$: 189.0732, found: 189.0732.

5-(p-tolyl)-2*H*-1,2,3-triazole-4-carboxamide (3b) — Synthesized as compound **3a** using 4-methylbenzaldehyde (130 µL, 1.11 mmol), 2-cyanoacetamide (102 mg, 1.22 mmol), NH_4Cl (206 mg, 3.89 mmol) and NaN_3 (216 mg, 3.33 mmol). The reaction provided 151 mg (68% yield, time = 6 h) of **3b** as brown solid; Mp: 261-263 °C; ^{1}H NMR (600 MHz, DMSO-d_6) δ_{ppm} 7.70 (d, J = 7.8 Hz, 2H), 7.24 (d, J = 7.8 Hz, 2H), 2.31 (s, 3H);. ^{13}C NMR (151 MHz, DMSO-d_6) δ_{ppm} 163.6, 139.6, 129.6, 129.2, 21.4.; FT-IR (KBr) 3377, 3257, 2983, 1661, 1533, 1409, 1083 cm^{-1}; HRMS (ESI) calcd. for $C_{10}H_{10}N_4O$ $[M + H]^+$: 203.0888, found: 203.0888.

5-(3-fluorophenyl)-2*H*-1,2,3-triazole-4-carboxamide (3c) — Synthesized as compound **3a** using 3-fluorobenzaldehyde (130 µL, 1.23 mmol), 2-cyanoacetamide (113 mg, 1.35 mmol), NH_4Cl (228 mg, 4.31 mmol) and NaN_3 (240 mg, 3.69 mmol). The reaction provided 190 mg (75% yield, time = 4 h) of **3c** as off-white solid; Mp: 277-279 °C; ^{1}H NMR (400 MHz, DMSO-

d_6) δ_{ppm} 7.94 (s, 1H), 7.87 (d, *J* = 10.6 Hz, 1H), 7.79 (d, *J* = 7.7 Hz, 1H), 7.60 (br s, 1H), 7.53 (dd, *J* = 14.5, 7.8 Hz, 1H), 7.27 (t, *J* = 7.7 Hz, 1H).; ^{13}C NMR (100 MHz, DMSO-d_6) δ_{ppm} 163.0, 162.7, 160.6, 130.3 (d, *J* = 8.5 Hz), 124.6, 115.6; FT-IR (KBr) 3379, 3267, 2974, 1657, 1529, 1411, 1089 cm^{-1}; HRMS (ESI) calcd. for $C_9H_7FN_4O$ [M + H]$^+$: 207.0637, found: 207.0640.

5-(3-chlorophenyl)-2*H*-1,2,3-triazole-4-carboxamide (3d) — Synthesized as compound **3a** using 3-chlorobenzaldehyde (100 µL, 0.89 mmol), 2-cyanoacetamide (81 mg, 0.97 mmol), NH_4Cl (165 mg, 3.12 mmol) and NaN_3 (174 mg, 2.67 mmol). The reaction provided 161 mg (82% yield, time = 4 h) of **3d** as white solid; Mp: 268-270 °C; ^{1}H NMR (400 MHz, DMSO-d_6) δ_{ppm} 8.07 (s, 1H), 7.95 (br s, 1H), 7.91-7.90 (m, 1H), 7.60 (br s, 1H), 7.49-7.48 (m, 2H); ^{13}C NMR (100 MHz, DMSO-d_6) δ_{ppm} 162.7, 142.0, 137.8, 132.9, 130.1, 128.7, 128.4, 127.2; FT-IR (KBr) 3394, 3271, 2981, 1653, 1527, 1421, 1092 cm^{-1}; HRMS (ESI) calcd. for $C_9H_7ClN_4O$ [M + H]$^+$: 223.0308, found: 223.0308.

5-(3-bromophenyl)-2*H*-1,2,3-triazole-4-carboxamide (3e) — Synthesized as compound **3a** using 3-bromobenzaldehyde (105 µL, 0.90 mmol), 2-cyanoacetamide (83 mg, 0.99 mmol), NH_4Cl (167 mg, 3.15 mmol) and NaN_3 (176 mg, 2.7 mmol). The reaction provided 192 mg (80% yield, time = 4 h) of **3e** as white solid; Mp: 279-281 °C; ^{1}H NMR (600 MHz, DMSOd$_6$) δ_{ppm} 8.18 (s, 1H), 7.96-7.93 (m, 2H), 7.62-7.59 (m, 2H), 7.44-7.42 (m, 1H).; ^{13}C NMR (100 MHz, DMSO-d_6) δ_{ppm} 162.7, 131.4 (d, *J* = 39.1 Hz), 130.5, 127.6, 121.5; FT-IR (KBr) 3397, 3279, 2973, 1655, 1521, 1429, 1107 cm^{-1}; HRMS (ESI) calcd. for $C_9H_7BrN_4O$ [M + H]$^+$: 266.9876, found: 266.9870.

5-(4-fluorophenyl)-2*H*-1,2,3-triazole-4-carboxamide (3f) — Synthesized as compound **3a** using 4-fluorobenzaldehyde (110 µL, 1.06 mmol), 2-cyanoacetamide (98 mg, 1.17 mmol), NH_4Cl (197 mg, 3.71 mmol) and NaN_3 (207 mg, 3.18 mmol). The reaction provided 184 mg (84% yield, time = 4 h) of **3f** as off-white solid; Mp: 276-278 °C; ^{1}H NMR (600 MHz, DMSO-d_6) δ_{ppm} 7.99 - 7.89 (m, 3H), 7.54 - 7.53 (m, 1H), 7.30 – 7.20 (m, 2H); ^{13}C NMR (151 MHz, DMSO-d_6) δ_{ppm} 162.8, 131.0, 119.0, 115.2, 115.1; FT-IR (KBr) 3387, 3271, 2977, 1649, 1520, 1423, 1103 cm^{-1}; HRMS (ESI) calcd. for $C_9H_7FN_4O$ [M + H]$^+$: 207.0677, found: 207.0677.

5-(4-chlorophenyl)-2*H*-1,2,3-triazole-4-carboxamide (3g) — Synthesized as compound **3a** using 4-chlorobenzaldehyde (100 mg, 0.71 mmol), 2-cyanoacetamide (66 mg, 0.79 mmol), NH_4Cl (132 mg, 2.49 mmol) and NaN_3 (138 mg, 2.13 mmol). The reaction provided 140 mg (88% yield, time = 4 h) of **3g** as white solid; Mp: 280-282 °C (in lit[7]: 282 – 283 °C); ^{1}H NMR (600 MHz, DMSO-d_6) δ_{ppm} 7.92 – 7.90 (m, 2H), 7.51 – 7.49 (m, 2H); ^{13}C NMR (151 MHz, DMSO-d_6) δ_{ppm} 163.4, 142.6, 137.9, 134.2, 130.9, 128.8, 128.0; FT-IR (KBr) 3389, 3273, 2970, 1641, 1528, 1433, 1105 cm^{-1}; HRMS (ESI) calcd. for $C_9H_7ClN_4O$ $[M + H]^+$: 223.0308, found: 223.0308.

5-(4-bromophenyl)-2*H*-1,2,3-triazole-4-carboxamide (3h) — Synthesized as compound **3a** using 4-bromobenzaldehyde (105 mg, 0.57 mmol), 2-cyanoacetamide (52 mg, 0.62 mmol), NH_4Cl (106 mg, 2 mmol) and NaN_3 (111 mg, 1.71 mmol). The reaction provided 126 mg (83% yield, time = 4 h) of **3h** as brown solid; Mp: 280-282 °C; ^{1}H NMR (400 MHz, DMSO-d_6) δ_{ppm} 7.91 – 7.89 (m, 3H), 7.67 – 7.65 (m, 2H), 7.57 (br s, 1H); ^{13}C NMR (100 MHz, DMSO-d_6) δ_{ppm} 162.7, 137.8, 131.2, 130.7, 122.3; FT-IR (KBr) 3391, 3269 2976, 1651, 1524, 1438, 1095 cm^{-1}; HRMS (ESI) calcd. for $C_9H_7BrN_4O$ $[M + H]^+$: 266.9876, found: 266.9876.

5-(3,4-difluorophenyl)-2*H*-1,2,3-triazole-4-carboxamide (3i) — Synthesized as compound **3a** using 3,4-difluorobenzaldehyde (160 µL, 1.45 mmol), 2-cyanoacetamide (134 mg, 1.60 mmol), NH_4Cl (269 mg, 5.08 mmol) and NaN_3 (283 mg, 4.35 mmol). The reaction provided 253 mg (78% yield, time = 4 h) of **3i** as pale yellow solid; Mp: 285-287 °C; ^{1}H NMR (600 MHz, DMSO-d_6) δ_{ppm} 8.14 – 8.10 (m, 1H), 7.95 (br s, 1H), 7.84-7.81 (m, 1H), 7.60 (br s, 1H), 7.55-7.50 (m, 1H).; ^{13}C NMR (151 MHz, DMSO-d_6) δ_{ppm} 162.8, 150.6, 149.9 (d, J = 12.5 Hz), 149.0 (d, J = 12.2 Hz), 148.3 (d, J = 12.5 Hz), 138.0, 125.8, 121.4, 118.1 (d, J = 18.8 Hz), 117.6 (d, J = 17.3 Hz).; FT-IR (KBr) 3393, 3269 2966, 1657, 1531, 1428, 1099 cm^{-1}; HRMS (ESI) calcd. for $C_9H_6F_2N_4O$ $[M + H]^+$: 225.0543, found: 225.0543.

5-(3,4-dichlorophenyl)-2*H*-1,2,3-triazole-4-carboxamide (3j) — Synthesized as compound **3a** using 3,4-dichlorobenzaldehyde (125 mg, 0.71 mmol), 2-cyanoacetamide (66 mg, 0.79 mmol), NH_4Cl (132 mg, 2.49 mmol) and NaN_3 (138 mg, 2.13 mmol). The reaction provided 158 mg (86% yield, time = 4 h) of **3j** as white solid; Mp: 286-288 °C; ^{1}H NMR (600 MHz, DMSO-d_6)

δ_{ppm} 8.24 (s, 1H), 7.89 – 7.87 (m, 1H), 7.69 (d, *J* = 8.4 Hz, 1H); ^{13}C NMR (151 MHz, DMSO-d$_6$) δ_{ppm} 162.9, 138.3, 132.0, 131.4, 130.9 (d, *J* = 13.9 Hz), 129.1; FT-IR (KBr) 3381, 3262 2976, 1654, 1533, 1418, 1097 cm^{-1}; HRMS (ESI) calcd. for $C_9H_6Cl_2N_4O$ $[M + H]^+$: 256.9991, found: 256.9991.

5-(3-chloro-4-fluorophenyl)-2*H*-1,2,3-triazole-4-carboxamide (3k) — Synthesized as compound **3a** using 3-chloro-4-fluorobenzaldehyde (130 µL, 0.80 mmol), 2-cyanoacetamide (73 mg, 0.88 mmol), NH_4Cl (148 mg, 2.8 mmol) and NaN_3 (156 mg, 2.4 mmol). The reaction provided 149 mg (78% yield, time = 5 h) of **3k** as pale yellow solid; Mp: 275-277 °C; ^{1}H NMR (600 MHz, DMSO-d$_6$) δ_{ppm} 8.26 (dd, *J* = 7.3, 2.1 Hz, 1H), 8.00-7.97 (m, 1H), 7.94 (br s, 1H), 7.60 (br s, 1H), 7.51 (t, *J* = 9.0 Hz, 1H).; ^{13}C NMR (151 MHz, DMSO-d$_6$) δ_{ppm} 162.6, 158.2, 156.5, 137.6, 130.8, 129.5 (d, *J* = 7.6 Hz), 127.0, 119.3 (d, *J* = 17.9 Hz), 116.8 (d, *J* = 21.1 Hz); FT-IR (KBr) 3384, 3265, 2971, 1646, 1543, 1424, 1114 cm^{-1}; HRMS (ESI) calcd. for $C_9H_6ClFN_4O$ $[M + H]^+$: 241.0214, found: 241.0212.

5-(3-bromo-4-fluorophenyl)-2*H*-1,2,3-triazole-4-carboxamide (3l) — Synthesized as compound **3a** using 3-bromo-4-fluorobenzaldehyde (110 µL, 0.53 mmol), 2-cyanoacetamide (49 mg, 0.58 mmol), NH_4Cl (98 mg, 1.86 mmol) and NaN_3 (103 mg, 1.59 mmol). The reaction provided 115 mg (76% yield, time = 6 h) of **3l** as brown solid; Mp: 261-263 °C; ^{1}H NMR (600 MHz, DMSO-d$_6$) δ_{ppm} 8.36 (dd, *J* = 6.7, 1.8 Hz, 1H), 8.03 – 8.00 (m, 1H), 7.95 (br s, 1H), 7.60 (br s, 1H), 7.48 (t, *J* = 8.7 Hz, 1H); ^{13}C NMR (151 MHz, DMSO-d$_6$) δ_{ppm} 162.6, 159.2, 157.6, 133.6, 130.2, 129.2, 120.2, 116.7, 116.5, 112.2, 107.8, 107.7; FT-IR (KBr) 3391, 3263, 2974, 1657, 1533, 1431, 1104 cm^{-1}; HRMS (ESI) calcd. for $C_9H_6BrFN_4O$ $[M + H]^+$: 284.9782, found: 284.9782.

5-(3,4-difluorophenyl)-2*H*-1,2,3-triazole-4-carboxylic acid (4a)[5] — To a stirring solution of ethyl 5-(3,4-difluorophenyl)-2H-1,2,3-triazole-4-carboxylate (**2i**, 2 g, 7.9 mmol) in MeOH:THF:H_2O (1:2:1, 15 mL) was added LiOH (1.9 g, 79 mmol) and allowed to stir at room temperature for 12 h. The progress of the reaction was monitored by TLC technique. After completion of the reaction, it was neutralized by 10% dilute HCl. The obtained solid precipitate was filtered and washed the residue with cold water. The solid residue was dried under vacuum

to provide the target compound 1.6 g (92% yield, time = 12 h) of **4a** as Dutch white solid**;** Mp: 162-164 °C; ^{1}H NMR (600 MHz, DMSO-d$_6$) δ_{ppm} 7.84 – 7.82 (m, 1H), 7.72 – 7.70 (m, 1H), 7.41 (s, 1H).; ^{13}C NMR (151 MHz, DMSO-d$_6$) δ_{ppm} 163.1, 154.4, 152.7, 128.4, 126.4, 121.7, 117.7; FT-IR (KBr) 3412, 3045, 1712, 1530, 1316, 1120, 1022 cm^{-1}; HRMS (ESI) calcd. for $C_9H_5F_2N_3O_2$ [M - H]$^-$: 224.0277, found: 224.0051.

5-(3,4-dichlorophenyl)-*N*-phenyl-2*H*-1,2,3-triazole-4-carboxamide (4b) — A mixture of 5-(3,4-dichlorophenyl)-2*H*-1,2,3-triazole-4-carboxylic acid (130 mg, 0.5 mmol), HOBt (149 mg, 1.1 mmol), EDC.HCl (211 mg, 1.1 mmol) and aniline (50 µL, 0.55 mmol) was vacuum under reduced pressure and filled N_2 gas thrice using N_2-ballon. The DMF (1 mL) was added to the resultant mixture and stirred for 5 minute at room temperature. Then DIPEA (260 µL, 1.5 mmol) was added drop-wise to the reaction mixture and allow stirring for 12 h at room temperature. The progress of the reaction was monitored by TLC technique. After completion the reaction, it was purified by column chromatography using MeOH/DCM (2-25%) solvent gradient to get the target compound 114 mg (68% yield, time = 14 h) of **4b** as brown solid; Mp: 155-157 °C; ^{1}H NMR (600 MHz, $CDCl_3$ + DMSO-d$_6$) δ_{ppm} 8.85 (s, 1H), 8.14 (s, 1H), 7.94 (d, *J* = 6.6 Hz, 1H), 7.64 (d, *J* = 7.8 Hz, 2H), 7.51 (d, *J* = 8.1 Hz, 1H), 7.35 (t, *J* = 7.7 Hz, 2H), 7.14 (t, *J* = 7.4 Hz, 1H).; ^{13}C NMR (151 MHz, $CDCl_3$ + DMSO-d$_6$) δ_{ppm} 158.8, 137.7, 132.3, 131.0 (d, *J* = 4.5 Hz), 130.3 (d, *J* = 4.6 Hz), 129.1 (d, *J* = 18, 5.6 Hz), 128.9, 124.5, 120.1; FT-IR (KBr) 3381, 2934, 1653, 1523, 1433, 1104 cm^{-1}; HRMS (ESI) calcd. for $C_{15}H_{10}Cl_2N_4O$ [M + H]$^+$: 334.0202, found: 334.0202.

***N*-(2-aminophenyl)-5-(3,4-dichlorophenyl)-2*H*-1,2,3-triazole-4-carboxamide (4c) —** Synthesized as compound **4b** using 5-(3,4-dichlorophenyl)-2*H*-1,2,3-triazole-4-carboxylic acid (140 mg, 0.54 mmol), HOBt (161 mg, 1.19 mmol), EDC.HCl (228 mg, 1.19 mmol), benzene-1,2-diamine (64 mg, 0.59 mmol) and DIPEA (280 µL, 1.62 mmol) provided 132 mg (70% yield, time = 14 h) of **4c** as white solid; Mp: 190-192 °C; ^{1}H NMR (600 MHz, DMSO-d$_6$) δ_{ppm} 9.54 (s, 1H), 8.50 (s, 1H), 8.15 – 8.13 (m, 1H), 7.61 (d, *J* = 8.5 Hz, 1H), 7.44 – 7.42 (m, 1H), 6.91 (td, *J* = 7.9, 1.5 Hz, 1H), 6.79 (dd, *J* = 7.9, 1.3 Hz, 1H), 6.63 – 6.60 (m, 1H).; ^{13}C NMR (151 MHz, DMSO-d$_6$) δ_{ppm} 161.3, 141.8, 141.7, 136.7, 133.5, 130.5, 130.0, 129.7, 129.1, 128.1, 125.3,

124.7, 124.5, 116.8, 116.5; FT-IR (KBr) 3415, 3381, 2930, 1647, 1521, 1413, 1311, 1104 cm^{-1}; HRMS (ESI) calcd. for $C_{15}H_{11}Cl_2N_5O$ $[M + H]^+$: 348.0413, found: 348.0396.

***N*-(2-aminophenyl)-5-(3,4-difluorophenyl)-2*H*-1,2,3-triazole-4-carboxamide (4d)** — Synthesized as compound **4b** using 5-(3,4-difluorophenyl)-2*H*-1,2,3-triazole-4-carboxylic acid (150 mg, 0.67 mmol), HOBt (198 mg, 1.47 mmol), EDC.HCl (282 mg, 1.47 mmol), benzene-1,2-diamine (80 mg, 0.74 mmol) and DIPEA (350 µL, 1.62 mmol) provided 140 mg (67% yield, time = 14 h) of **4d** as pale yellow solid; Mp: 170-172 °C; ^1H NMR (400 MHz, MeOD-d_4) δ_{ppm} 7.92 (d, *J* = 12.6 Hz, 1H), 7.83 (d, *J* = 8.4 Hz, 1H), 7.32 (d, *J* = 7.8 Hz, 1H), 7.22 (t, *J* = 8.5 Hz, 1H), 7.06 (t, *J* = 7.6 Hz, 1H), 6.90 (d, *J* = 8.0 Hz, 1H), 6.77 (t, *J* = 7.6 Hz, 1H); ^{13}C NMR (100 MHz, MeOD-d_4) δ_{ppm} 162.2, 157.0, 154.5, 143.5, 138.9, 130.0, 128.5, 127.4, 127.0, 124.9, 122.2, 119.8, 118.7 (d, *J* = 13.0 Hz), 118.4; FT-IR (KBr) 3408, 3373, 2937, 1641, 1533, 1417, 1318, 1101 cm^{-1}; HRMS (ESI) calcd. for $C_{15}H_{11}F_2N_5O$ $[M + H]^+$: 316.1004, found: 316.0992.

***N*-((1r,3r,5r,7r)-adamantan-2-yl)-5-(3,4-dichlorophenyl)-2*H*-1,2,3-triazole-4-carboxamide (4e)**[6] — Synthesized as compound **4b** using 5-(3,4-dichlorophenyl)-2*H*-1,2,3-triazole-4-carboxylic acid (100 mg, 0.39 mmol), HOBt (116 mg, 0.86 mmol), EDC.HCl (164 mg, 0.86 mmol), adamantylamine (65 mg, 0.43 mmol) and DIPEA (204 µL, 1.17 mmol) provided 83 mg (55% yield, time = 12 h) of **4e** as white solid; Mp: 244-246 °C; ^1H NMR (600 MHz, $CDCl_3$) δ_{ppm} 8.03 (s, 1H), 7.90 (d, *J* = 7.6 Hz, 1H), 7.49 (d, *J* = 8.4 Hz, 1H), 2.11 (s, 10H), 1.72 – 1.67 (m, 5H); ^{13}C NMR (151 MHz, $CDCl_3$ + DMSO-d_6) δ_{ppm} 159.2, 143.8, 131.4, 130.1, 129.4, 128.2, 51.3, 40.8, 35.6, 28.6; FT-IR (KBr) 3391, 2944, 1655, 1533, 1466, 1431, 1104 cm^{-1}; HRMS (ESI) calcd. for $C_{19}H_{20}Cl_2N_4O$ $[M + H]^+$: 392.0985, found: 392.0981.

tert-butyl(2-(5-(3,4-difluorophenyl)-2*H*-1,2,3-triazole-4-carboxamido)ethyl) carbamate (4f) — Synthesized as compound **4b** using 5-(3,4-difluorophenyl)-2*H*-1,2,3-triazole-4-carboxylic acid (500 mg, 2.22 mmol), HOBt (660 mg, 4.88 mmol), EDC.HCl (937 mg, 4.88 mmol), tert-butyl (2-aminoethyl)carbamate (390 mg, 2.44 mmol) and DIPEA (1.2 mL, 6.66 mmol) provided 604 mg (74% yield, time = 18 h) of **4f** as white solid; Mp: 155-157 °C; ^1H NMR (600 MHz, DMSO-d_6) δ_{ppm} 8.57 (t, *J* = 5.6 Hz, 1H), 8.09 – 8.06 (m, 1H), 7.87 – 7.85 (m, 1H), 7.39 (t, *J* = 8.6 Hz, 1H), 6.89 (t, *J* = 5.5 Hz, 1H), 3.32 – 3.29 (m, 2H), 3.12 – 3.09 (m, 2H), 1.35 (s, 9H); ^{13}C

NMR (151 MHz, DMSO-d_6) δ_{ppm} 160.8, 155.7, 154.0, 152.4, 137.7, 127.6, 125.5, 121.2, 117.0 (d, *J* = 21.9 Hz), 77.7, 45.5, 28.2, 8.5; FT-IR (KBr) 3441, 3393, 2921, 1647, 1533, 1415, 1364, 1099 cm^{-1}; MS (ESI) calcd. for $C_{16}H_{19}F_2N_5O_3$[M + Na]$^+$: 391.16, found: 391.16.

(Z)-5-(3,4-difluorophenyl)-*N*-(1-(hydroxyimino)-2-phenylethyl)-2*H*-1,2,3-triazole-4-carboxamide (4g) — Synthesized as compound **4b** using 5-(3,4-difluorophenyl)-2*H*-1,2,3-triazole-4-carboxylic acid (120 mg, 0.53 mmol), HOBt (158 mg, 1.17 mmol), EDC.HCl (225 mg, 1.17 mmol), N-hydroxy-2-phenylacetimidamide (87 mg, 0.58 mmol) and DIPEA (276 µL, 1.59 mmol) provided 118 mg (62% yield, time = 16 h) of **4g** as pale yellow solid; Mp: 152-154 °C; ^{1}H NMR (400 MHz, MeOD-d_4) δ_{ppm} 7.81 – 7.78 (m, 1H), 7.72 (d, *J* = 8.2 Hz, 1H), 7.38-7.36 (m, 2H), 7.30 (t, *J* = 7.4 Hz, 2H), 7.26 – 7.21 (m, 2H), 3.51 (s, 2H); ^{13}C NMR (100 MHz, MeOD-d_4) δ_{ppm} 161.1, 160.5, 156.9, 154.5, 137.5, 130.7 (d, *J* = 11.1 Hz), 129.9 (d, *J* = 15.7 Hz), 128.3, 127.2 (d, *J* = 3.6 Hz), 122.3, 118.8, 118.6, 38.0; FT-IR (KBr) 3423, 3379, 2942, 1649, 1513, 1425, 1324, 1107 cm^{-1}; HRMS (ESI) calcd. for $C_{17}H_{13}F_2N_5O_2$ [M + H]$^+$: 358.1071, found: 358.1069.

(Z)-5-(3,4-difluorophenyl)-*N*-(2-(3,4-difluorophenyl)-1-(hydroxyimino)ethyl)-2*H*-1,2,3-triazole-4-carboxamide (4h) — Synthesized as compound **4b** using 5-(3,4-difluorophenyl)-2*H*-1,2,3-triazole-4-carboxylic acid (125 mg, 0.56 mmol), HOBt (165 mg, 1.22 mmol), EDC.HCl (234 mg, 1.22 mmol), 2-(3,4-difluorophenyl)-N-hydroxyacetimidamide (115 mg, 0.62 mmol) and DIPEA (292 µL, 1.68 mmol) provided 142 mg (65% yield, time = 14 h) of **4h** as pale yellow solid; Mp: 135-137 °C; ^{1}H NMR (400 MHz, MeOD-d_4) δ_{ppm} 7.83 (dd, *J* = 12.4, 1.9 Hz, 1H), 7.76 – 7.74 (m, 1H), 7.35– 7.27 (m, 2H), 7.23 – 7.19 (m, 2H), 3.50 (s, 2H).; ^{13}C NMR (100 MHz, MeOD-d_4) δ_{ppm} 160.5, 157.0, 154.5, 152.7, 152.2, 149.7 (d, *J* = 12.6 Hz), 135.1, 130.7 (d, *J* = 11.4 Hz), 127.3 (d, *J* = 3.6 Hz), 126.5 (dd, *J* = 6.4, 3.6 Hz), 122.4 (d, *J* = 1.5 Hz), 119.0 – 118.3 (m), 37.1; FT-IR (KBr) 3419, 3377, 2943, 1654, 1527, 1419, 1329, 1109 cm^{-1}; HRMS (ESI) calcd. for $C_{17}H_{11}F_4N_5O_2$ [M + H]$^+$: 394.0882, found: 394.0882.

(Z)-5-(3,4-difluorophenyl)-N-(1-(hydroxyimino)-2-(4-hydroxyphenyl)ethyl)-2H-1,2,3-triazole-4-carboxamide (4i) — Synthesized as compound **4b** using 5-(3,4-difluorophenyl)-2*H*-1,2,3-triazole-4-carboxylic acid (135 mg, 0.60 mmol), HOBt (178 mg, 1.32 mmol), EDC.HCl

(253 mg, 1.32 mmol), N-hydroxy-2-(4-hydroxyphenyl) acetimidamide (120 mg, 0.66 mmol) and DIPEA (312 μL, 1.8 mmol) provided 123 mg (55% yield, time = 18 h) of **4i** as Dutch white solid; Mp: 162-164 °C; ^{1}H NMR (600 MHz, DMSO-d_6) δ_{ppm} 7.82 (dd, J = 12.5, 1.8 Hz, 1H), 7.71 – 7.70 (m, 2H), 7.41 (t, J = 8.6 Hz, 2H), 7.13 (d, J = 8.5 Hz, 1H), 6.72 – 6.71 (m, 1H), 4.27 (s, 1H), 4.03 (s, 1H), 3.83 (s, 2H).; ^{13}C NMR (151 MHz, DMSO-d_6) δ_{ppm} 170.5, 156.3, 154.0, 152.3, 129.9, 128.1 (d, J = 10.8 Hz), 126.0 (d, J = 3.2 Hz), 125.8 (d, J = 7.8 Hz), 125.6, 121.3, 119.1 (d, J = 18.2 Hz), 117.4 – 117.1 (m), 115.4, 52.0; FT-IR (KBr) 3421, 3381, 2945, 1654, 1527, 1423,1104 cm^{-1}; HRMS (ESI) calcd. for $C_{17}H_{13}F_2N_5O_3$ $[M]^+$: 373.0571, found: 373.0564.

(Z)-*N*-((4-acetamidophenyl)(hydroxyimino)methyl)-5-(3,4-difluorophenyl)-2*H*-1,2,3-triazole-4-carboxamide (4j) — Synthesized as compound **4b** using 5-(3,4-difluorophenyl)-2*H*-1,2,3-triazole-4-carboxylic acid (130 mg, 0.58 mmol), HOBt (172 mg, 1.27 mmol), EDC.HCl (244 mg, 1.27 mmol), N-(4-(N-hydroxycarbamimidoyl)phenyl)acetamide (123 mg, 0.64 mmol) and DIPEA (303 μL 1.74 mmol) provided 131 mg (57% yield, time = 18 h) of **4j** as yellow solid; Mp: 141-143 °C; ^{1}H NMR (600 MHz, DMSO-d_6) δ_{ppm} 9.74 (s, 1H), 8.39 (s, 1H), 7.70 – 7.67 (m, 1H), 7.54 – 7.53 (m, 1H), 7.40 (d, J = 8.9 Hz, 2H), 7.28 (d, J = 8.9 Hz, 2H), 5.75 (s, 1H), 1.99 (s, 3H).; ^{13}C NMR (151 MHz, DMSO-d_6) δ_{ppm} 167.7, 156.0, 135.9, 133.1, 120.1, 119.6, 119.2, 118.1, 23.8; FT-IR (KBr) 3420, 3391, 2931, 1644, 1532, 1422,1099 cm^{-1}; HRMS (ESI) calcd. for $C_{18}H_{14}F_2N_6O_3$ $[M + H]^+$: 401.1129, found: 401.1127.

***N*-(2-aminoethyl)-5-(3,4-difluorophenyl)-2*H*-1,2,3-triazole-4-carboxamide —** The tert-butyl (2-(5-(3,4-difluorophenyl) -2*H*-1,2,3-triazole-4-carboxamido) ethyl)carbamate (**4f,** 500 mg, 1.36 mmol) is stirred with 30% TFA/DCM (4 mL) at room temperature for 2h. The progress of the reaction was monitored by TLC technique. After completion of the reaction, the solvent was removed under reduced pressure. Then the reaction mixture was washed with diethyl ether, and the obtained solid was dried under vacuum to provide 360 mg (98% yield) of target compound as Dutch white solid; Mp: 178-180 °C; HRMS (ESI) calcd. for $C_{11}H_{11}F_2N_5O$ $[M + H]^+$: 268.1004, found: 268.1001.

tert-butyl(*N*-(2-(5-(3,4-difluorophenyl)-2*H*-1,2,3-triazole-4-carboxamido) ethyl) sulfamoyl) carbamate[7] —

(Step I). **Synthesis of tert-butyl (chlorosulfonyl) carbamate:** The chlorosulfonyl isocyanate (300 µL, 3.46 mmol) was stirred in 3 mL dry DCM at -5 °C for 5 minutes. Then *t*-BuOH (330 µL, 3.46 mmol) was added dropwise to the reaction mixture so that temperature does not exceed 0 °C and allow stirring for 10 minutes. Then the reaction mixture was warm to room temperature, and it was stirred for 2h.

(Step II) **Synthesis of tert-butyl(*N*-(2-(5-(3,4-difluorophenyl)-2*H*-1,2,3-triazole-4-carboxamido) ethyl) sulfamoyl) carbamate**: *N*-(2-aminoethyl)-5-(3,4-difluorophenyl)-2*H*-1,2,3-triazole-4-carboxamide (300 mg, 1.12 mmol) was dissolved in 5 mL dry DCM under N_2-atmosphere. Then the temperature of the reaction mixture was cool to -15 °C using the ice-salt bath. Then the above reaction mixture of step I was added drop-wise to the resultant reaction mixture so that the temperature does not exceed -10 °C and stir it for 10 minutes. Then dry triethylamine (470 µL, 3.37 mmol) was added drop-wise to the reaction mixture and stirring for 10 minutes at 0 °C . Then the reaction mixture was warm to room temperature and stirred for 20 h. The progress of the reaction was monitored by TLC technique. After completion of the reaction, the solvent was removed under reduced pressure. Then it was neutralized using 10% aq HCl, and the mixture was diluted with ethyl acetate. The organic layer was extracted and washed with brine and dried over anhydrous Na_2SO_4. Then the organic solvent was removed under reduced pressure. Then the obtained precipitate was washed with diethyl ether. Then the solid was dried under vacuum to afford 202 mg (52% yield) of target compound as brown hygroscopic solid; MS (ESI) calcd. for $C_{16}H_{20}F_2N_6O_5S$ $[M + H]^+$: 447.12, found: 447.12.

5-(3,4-difluorophenyl)-*N*-(2-(sulfamoylamino)ethyl)-2*H*-1,2,3-triazole-4-carboxamide (4k) — The tert-butyl(N-(2-(5-(3,4-difluorophenyl)-2H-1,2,3-triazole-4-carboxamido) ethyl) sulfamoyl) carbamate (200 mg, 0.45 mmol) is stirred with 30% TFA/DCM (2 mL) at room temperature for 4h. The progress of the reaction was monitored by TLC technique. After completion of the reaction, the solvent was removed under reduced pressure. Then the reaction mixture was washed with diethyl ether and the obtained solid was dried under vacuum to provided 130 mg (82% yield) of target compound as reddish brown hygroscopic solid; ^1H NMR (600 MHz, DMSO-d_6) δ_{ppm} 8.79 (br s, 1H), 8.11 (dd, *J* = 12.9, 1.8 Hz, 1H), 7.96 (br s, 1H), 7.87 (dd, *J* = 8.4, 1.2 Hz, 1H), 7.40 (t, *J* = 8.6 Hz, 1H), 3.56 – 3.53 (m, 2H), 3.02 – 3.00 (m, 2H); ^{13}C

NMR (151 MHz, DMSO-d_6) δ_{ppm} 161.3, 158.4, 154.0, 152.3, 137.3, 127.8, 125.7, 121.2, 118.2, 117.3, 117.2, 38.7, 36.6; FT-IR (KBr) 3461, 3395, 2927, 1643, 1536, 1414, 1367, 1115, 1063 cm^{-1}; MS (ESI) calcd. for $C_{11}H_{12}F_2N_6O_3S$ $[M + H]^+$: 347.06, found: 347.06.

5-(3,4-dichlorophenyl)-*N*-(2-(sulfamoylamino)ethyl)-2*H*-1,2,3-triazole-4-carboxamide (4l) — Synthesized as compound **4k** using tert-butyl (*N*-(2-(5-(3,4-dichlorophenyl)-2*H*-1,2,3-triazole-4-carboxamido)ethyl)sulfamoyl)carbamate (160 mg, 0.33 mmol) and 30% TFA/DCM (2 mL) provided 108 mg (85% yield, time = 4 h) of **3f** as brown hygroscopic solid; ^{1}H NMR (600 MHz, D_2O) δ_{ppm} 7.90 (s, 1H), 7.66 (d, J = 8.4 Hz, 1H), 7.61 (dd, J = 8.4, 2.0 Hz, 1H), 3.60 (t, J = 6.2 Hz, 2H), 3.26 – 3.23 (m, 2H); ^{13}C NMR (151 MHz, D_2O) δ_{ppm} 162.7, 136.9, 133.3, 132.0, 130.7, 130.5, 130.4, 127.1, 46.7, 39.0; FT-IR (KBr) 3441, 3384, 2931, 1641, 1539, 1429, 1351, 1105, 1045 cm^{-1}; MS (ESI) calcd. for $C_{11}H_{12}Cl_2N_6O_3S$ $[M + H]^+$: 379.01, found: 379.01.

Purification of the compounds by HPLC analysis

The purity of the synthesized compounds was analyzed by analytical HPLC analysis using Waters 600E HPLC system with an Ascentis® express C18, 2.7 µm HPLC column.[8-11] Methanol/water isocratic was used as the mobile phase at a flow rate of 0.5 mL/minute for 20 minutes run time. All the compounds (~ 2 mg) were dissolved in 1 mL of methanol/water, and HPLC analysis was performed by injecting 20 µL of the compound solution using a UV-detector at 260 nm. This purification process was repeated for more than ten times, and the fractions (with pure compounds only) were collected. The collected fractions for each compound were dried under reduced pressure and verified by HRMS analysis. These purified compounds were used to perform biological activities of the compounds.

Table S1. HPLC based IDO1 inhibition assays of the selected compounds

Compound	% Of inhibition for IDO1 enzyme[a]						IC_{50} (μM)
	32 nM	160 nM	800 nM	4 μM	20 μM	100 μM	
**2a**	26	31	35	61	76	81	2.789 ± 0.066
**2i**	21	29	59	60	78	80	0.396 ± 0.010
**2j**	24	31	52	65	77	77	0.257 ± 0.009
**3a**	11	31	36	63	79	80	1.192 ± 0.039
**3i**	27	48	69	76	84	83	0.119 ± 0.013
**3j**	39	60	70	88	89	89	0.099 ± 0.024
**4a**	11	13	29	30	57	65	8.944 ± 0.087
**4d**	20	48	79	84	86	88	0.172 ± 0.019
**4i**	13	34	52	68	79	84	0.114 ± 0.010
**4k**	14	45	69	77	86	84	0.059 ± 0.006
**4l**	33	57	65	88	93	94	0.098 ± 0.007
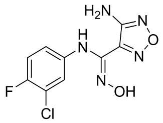	39	60	68	88	89	91	0.089 ± 0.002

[a]% of enzyme inhibition values are the mean of three independent assays.

Table S2. Characteristic peaks from the UV-Vis measurements of the IDO1 enzyme and potent compounds.

Sl. No.	Compound[a]	λ_{max} (nm) Fe^{3+} state binding	λ_{max} (nm) Fe^{2+} state binding
1	Only enzyme[b]	404, 526, 561, 630	421, 528, 551
2	**2i**	411, 535, 568, 645	427, 532, 561
3	**3i**	409, 531, 565, 643	427, 530, 561
4	**4i**	409, 537, 566, 645	428, 531, 564
5	**4k**	412, 537, 566, 647	427, 528, 562

[a]Compound conc. = 20 μM; [b]Enzyme conc. = 650 nM

SPR Analysis:

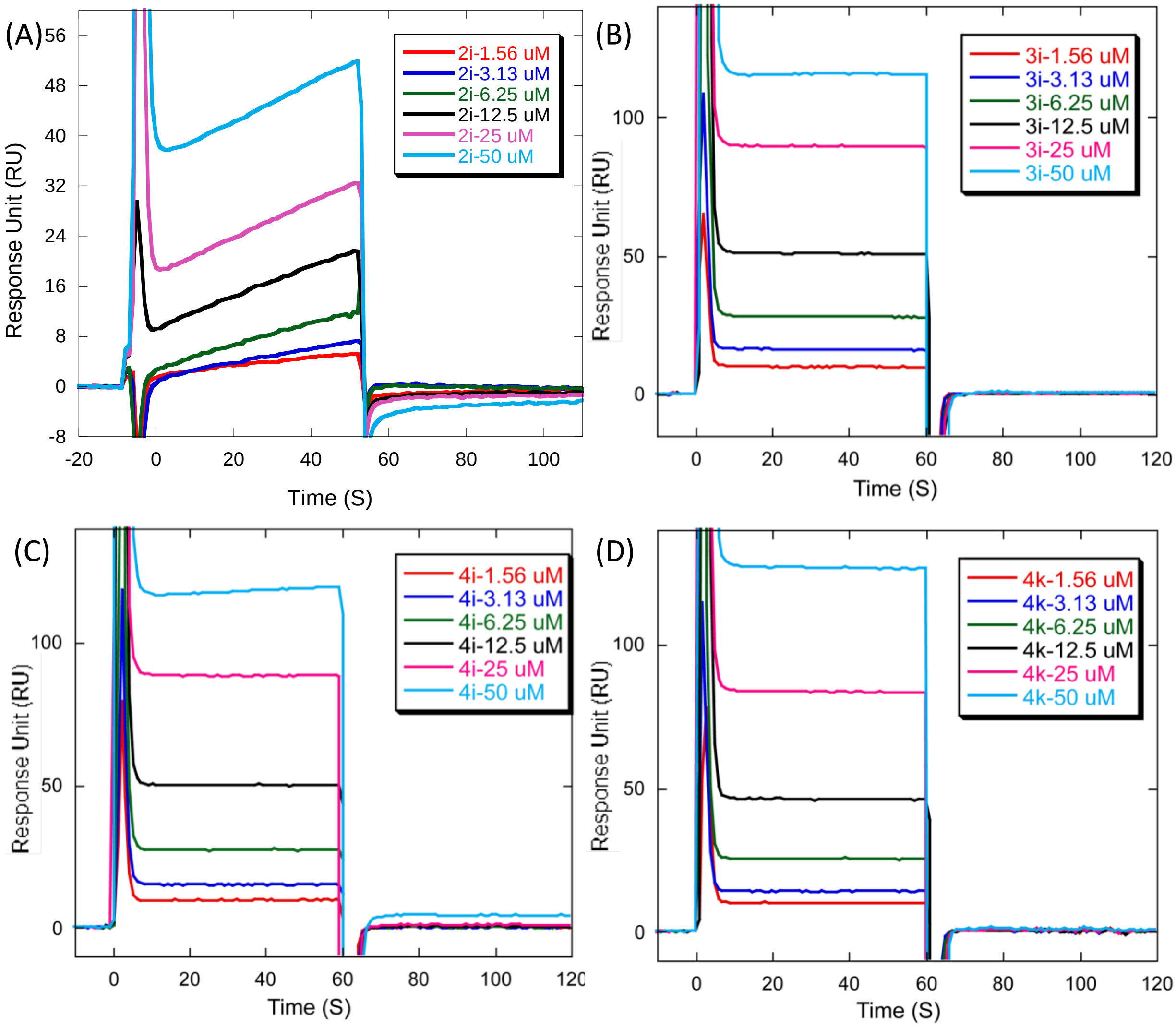

Figure S1. Measurements of compound binding affinity of the human IDO1 enzyme by equilibrium SPR analysis. The concentrations of the compounds **2i** (A), **3i** (B), **4i** (C), and **4k** (D), respectively injected over the biosensor chip surface-immobilized with IDO-1 protein are indicated. PBS buffer at pH 7.4 was used for all measurements. Binding isotherm was generated from the R_{eq} (average of triplicate measurements) *versus* the concentration of human IDO1 enzyme plot. K_d values determined by nonlinear least-squares analysis of the isotherm using the following equation: $R_{eq} = R_{max}/(1 + K_d/P)$.

Table S3. IDO1 binding affinity of various compounds determined from equilibrium SPR analysis

Compound	IDO1 enzyme (K_d (μM))
**2i**	61.652 ± 6.066
**3i**	34.539 ± 5.205
**4i**	40.225 ± 4.992
**4k**	49.116 ± 8.511

Values represent the mean from triplicate measurements. All measurements were performed in PBS buffer at pH 7.4.

Table S4: Docking parameters for the interaction of IDO1 enzyme with 1,2,3-triazoles

Compound	Rerank score (kJ/mol)[a]	Interaction (kJ/mol)[b]	Internal (kJ/mol)[c]	H-Bond (kJ/mol)[d]	LE1[e]	LE3[f]
**2i**	-100.526	-149.777	7.081	-6.786	-7.928	-5.585
**3i**	-90.342	-135.299	3.749	-5.459	-8.221	-5.646
**4i**	-90.196	-134.814	3.664	-5.037	-8.197	-5.637
**4k**	-123.728	-180.061	6.044	-4.976	-7.576	-5.379

[a]The rerank score is a linear combination of E-inter (steric, Van der Waals, hydrogen bonding, electrostatic) between the ligand and the protein, and E-intra. (torsion, sp2-sp2, hydrogen bonding, Van der Waals, electrostatic) of the ligand weighted by pre-defined coefficients (kJ/mol).

[b]The total interaction energy between the pose and the protein (kJ/mol).

[c]The internal energy of the pose (kJ/mol).

[d]Hydrogen bonding energy (kJ/mol).

[e]Ligand efficiency 1: MolDock scores divided by heavy atoms count.

[f]Ligand efficiency 3: Rerank scores divided by heavy atoms count.

Table S5. Circular dichroism analysis of IDO1 enzyme in the presence of selected 1,2,3-triazole compounds

Compounds	Concentration (µM)	% of Secondary structural elements			
		Helix	Beta	Turn	Random
**2i**	0	29.5	9.5	30.5	30.5
	0.25	28	0	34.1	37.9
	0.5	28.7	0	35.2	36.1
	1	30.2	0	36.3	33.5
	2	30.5	0	35.7	33.8
	4	30.5	0	33.8	35.7
**3i**	0	29.5	9.5	30.5	30.5
	0.25	30.1	0	34.1	35.8
	0.5	29.4	0	33.8	36.8
	1	29.8	0	34.5	35.8
	2	28.5	0	30.7	40.8
	4	30.7	0	8.9	60.4
**4i**	0	29.5	9.5	30.5	30.5
	0.25	27.8	0.7	33.9	37.6
	0.5	28	0	33.9	38.1
	1	27.6	0	31.8	40.6
	2	29.8	0	27.6	42.6
	4	31.8	0	29.4	38.8
**4k**	0	29.5	9.5	30.5	30.5
	0.25	26.8	3.6	35.3	34.3
	0.5	30.6	0	38.2	31.2
	1	28	0	38.1	33.9
	2	23.8	0	30.2	46.0
	4	29.1	0	34.5	36.4

Mode of IDO1 enzyme inhibition:

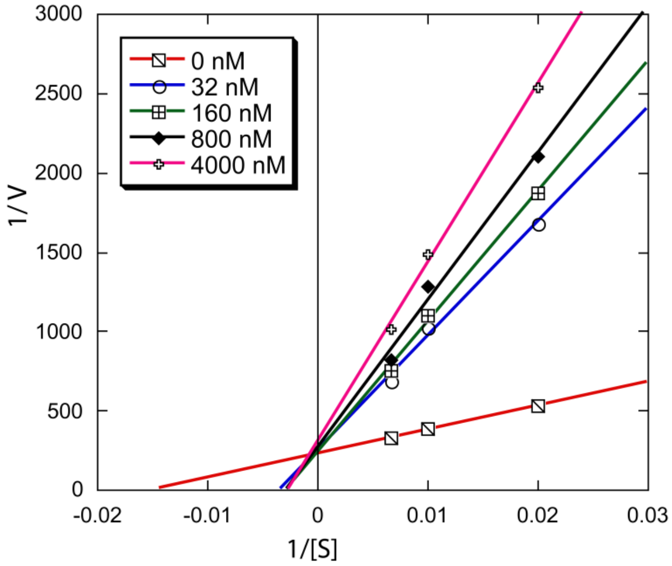

Figure S2. Determination of mode of IDO1 inhibition by the selected compound. The plot of 1/V against 1/[S] at different concentrations of the compound **2i**. Concentrations of L-Trp were 0, 50, 100 and 150 μM. The concentrations of compounds were varied from 0.032 to 4 μM. All the absorption measurements were performed in 100 mM phosphate buffer pH 6.5 at room temperature.

Table S6. Enzyme kinetics parameters of the IDO1 enzyme in the presence of selected triazoles

Compound	Mode of IDO1 inhibition	K_i (μM)[a]
**2i**	Competitive	0.241 ± 0.038
**3i**	Competitive	0.160 ± 0.015
**4i**	Competitive	0.258 ± 0.022
**4k**	Competitive	0.098 ± 0.011

[a]K_m = 59.76 μM and K_{cat} = 6.18 sec^{-1}

Table S7. Inhibitory activity of the 1,2,3-triazoles against purified human TDO enzyme

Compound	% of Enzyme inhibition[a]					
	32 nM	160 nM	800 nM	4 µM	20 µM	100 µM
**1a**	8	10	19	28	40	59
**2a**	8	17	19	23	27	34
**2g**	16	18	23	28	36	46
**2i**	2	9	12	15	23	28
**2j**	8	12	25	30	44	53
**3a**	5	7	19	23	29	42
**3g**	5	8	15	27	35	39
**3i**	5	11	14	26	33	49
**3j**	6	9	20	27	32	60
**4a**	4	8	16	18	22	30
**4d**	4	6	7	26	31	36
**4i**	4	7	14	24	42	55
**4k**	3	6	16	32	45	50
**4l**	3	10	11	19	25	26
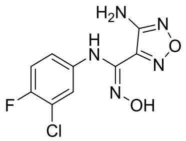	2	6	8	16	21	46

[a]% of enzyme inhibition values are the mean of three independent assays.

Table S8. HPLC based TDO inhibition assays of the selected compounds

Compound	% Of inhibition for TDO enzyme[a]					
	32 nM	160 nM	800 nM	4 μM	20 μM	100 μM
**2a**	9	39	49	68	68	73
**2i**	34	47	49	65	63	83
**2j**	1	35	36	49	41	43
**3a**	3	4	17	22	25	30
**3i**	10	18	21	18	37	52
**3j**	33	39	42	47	51	51
**4a**	6	18	26	38	41	54
**4d**	9	19	33	56	64	70
**4i**	15	25	26	50	66	73
**4k**	13	24	46	58	63	68
**4l**	14	15	22	29	38	55
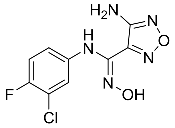	8	9	17	30	29	33

[a]IC_{50} values of these compounds could not be measured properly because of the linear relationship between kynurenine generation and compound concentrations.

Analysis of the serum binding ability of the potent compounds:

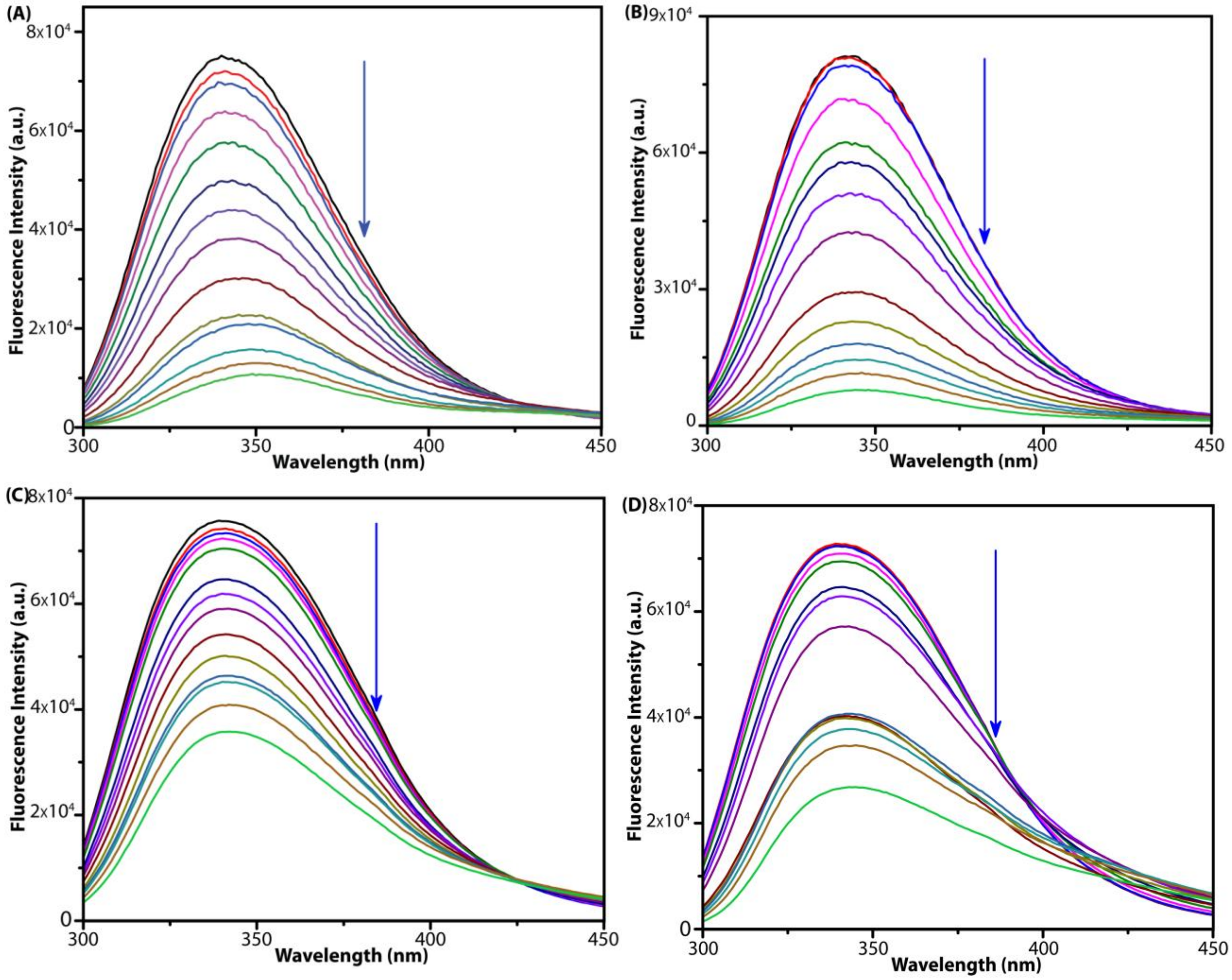

Figure S3: Trp-fluorescence emission measurements of human serum in the presence of the compounds. Plots represent the Trp fluorescence quenching spectra of serum in the presence of compounds **2i** (A), **3i** (B), **4i** (C), and **4k** (D), respectively. Compound concentrations were varied from 0 to 220 µM, and fixed amount of serum was used all measurements.

Measurement of Cytotoxicity of the Potent Compounds:

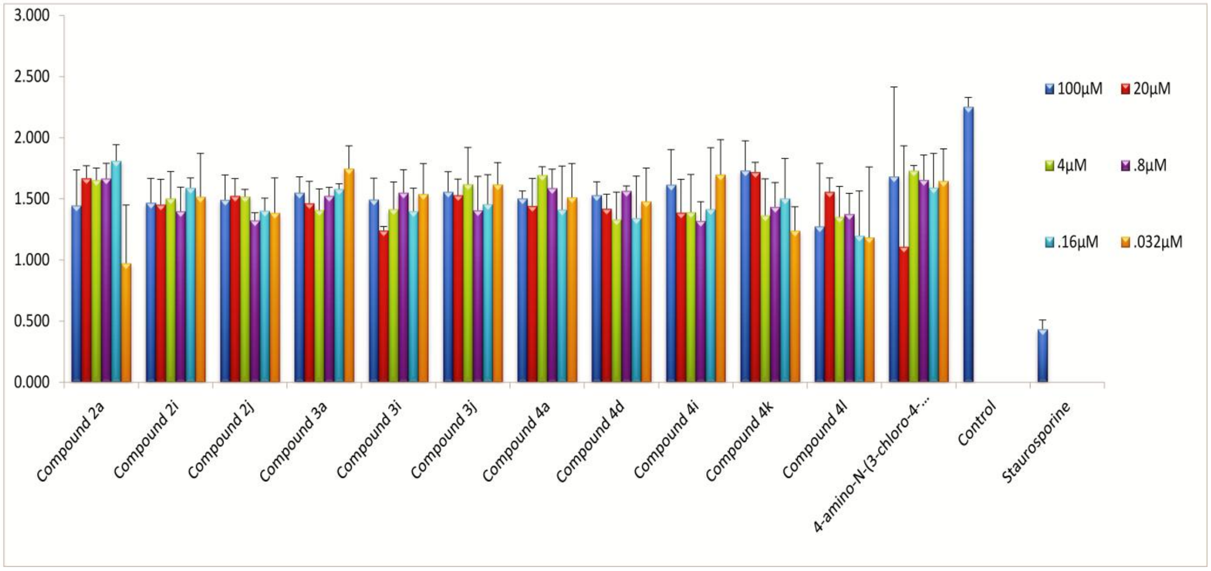

Figure S4. Effect of the selected 1,2,3-triazole derivatives on the viability of HEK-293 cells. The HEK-293 cells were treated with the indicated concentrations of the compounds for 48 h. Cell viability was determined by the MTT assay. The absorbance of different amounts of formazan was plotted against the mentioned concentrations of the compounds (0.032-100 μM). Absorbances are averages with standard deviation (error bars) from three independent experiments.

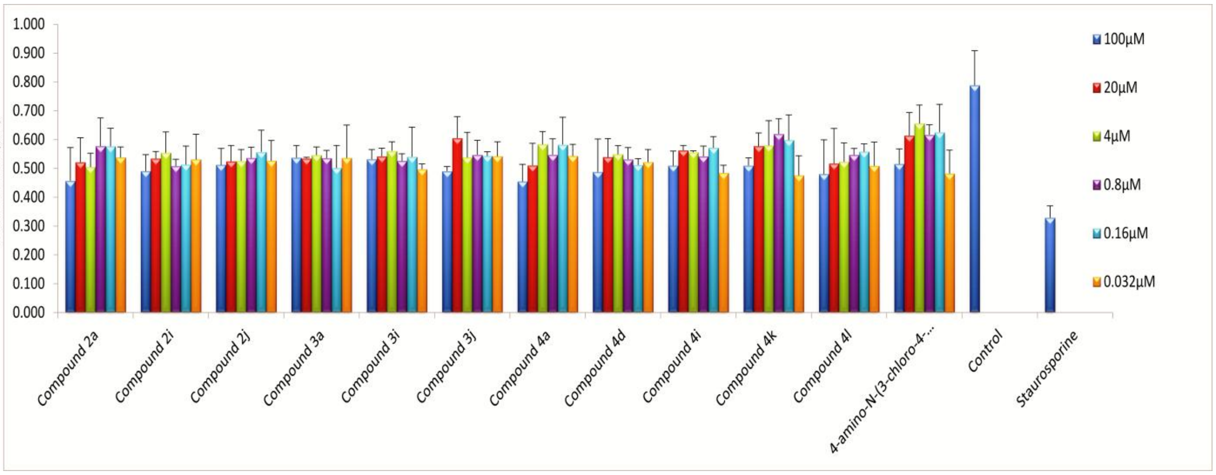

Figure S5. Effect of the selected 1,2,3-triazole derivatives on the viability of MDA-MB-231 cells. The MDA-MB-231 cells were treated with the indicated concentrations of the compounds for 48 h. Cell viability was determined by the MTT assay. The absorbance of different amounts of formazan was plotted against the mentioned concentrations of the compounds. Absorbances are averages with standard deviation (error bars) from three independent experiments.

Table 10. EC_{50} values of the selected compounds in MDA-MB-231 cells

Compound	MDA-MB-231 cells[a] EC_{50} (μM)[b]	Compound	MDA-MB-231 cells[a] EC_{50} (μM)[b]
**2a**	3.263 ± 0.045	**4a**	1.231 ± 0.021
**2i**	0.162 ± 0.015	**4d**	0.182 ± 0.013
**2j**	0.160 ± 0.007	**4i**	0.088 ± 0.010
**3a**	1.296 ± 0.068	**4k**	0.077 ± 0.005
**3i**	0.074 ± 0.017	**4l**	0.083 ± 0.005
**3j**	0.053 ± 0.004		

[a] IDO1 protein expression in MDA-MB-231 cells was induced by human IFN-γ (50 ng/mL).

[b] EC_{50} values are the mean of three independent assays.

T cell Activity Studies:

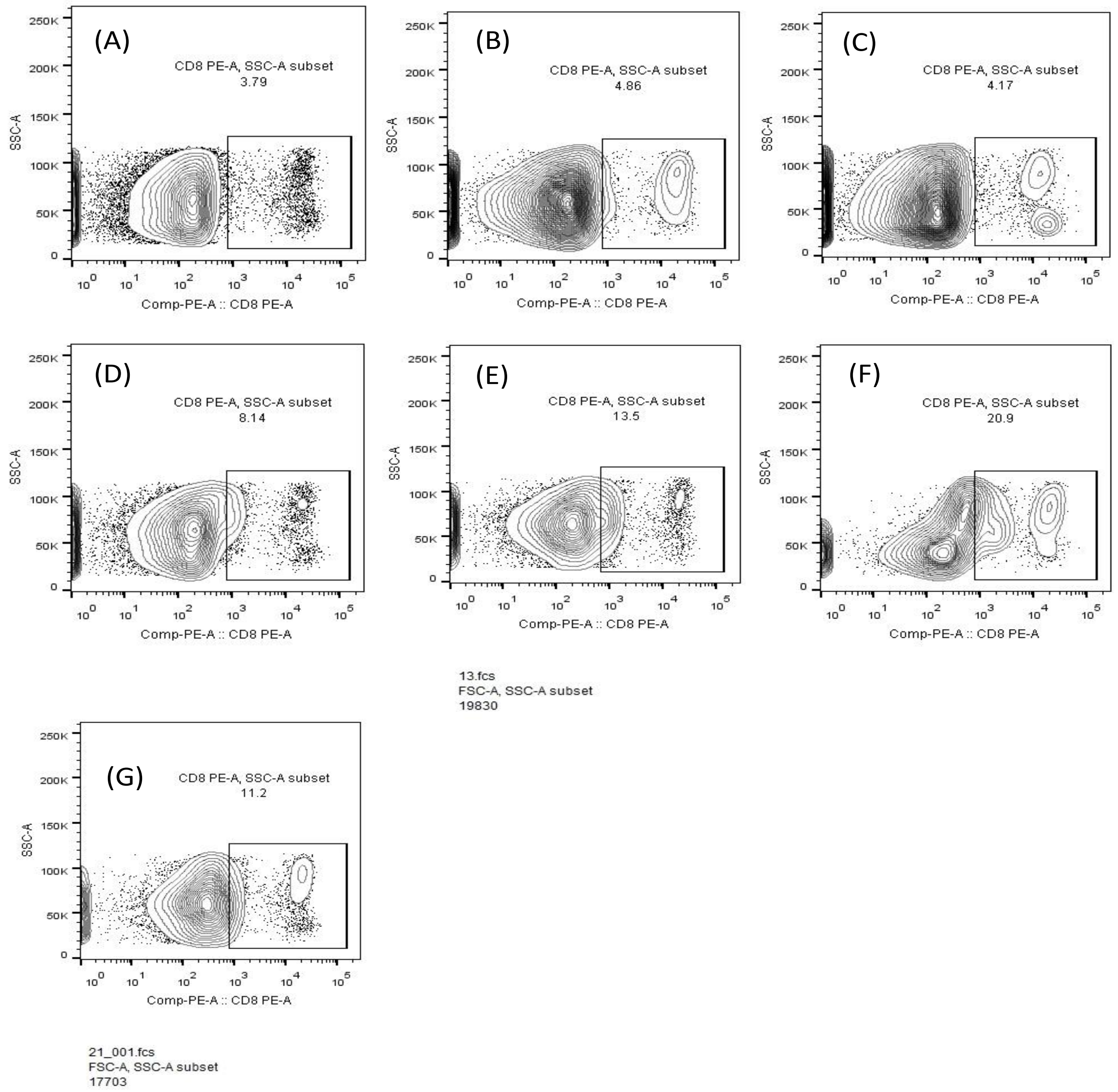

Figure S6. Up-regulation of CD8+T cells in tumor micro-environment post-treatment. Only cell (A), activated cell (B), control cell in tumor micro-environment (C), for **2i** (D), **3i** (E), **4i** (F), and **4K** (G). The concentration of the compounds was 50 μM for the T cell activity studies.

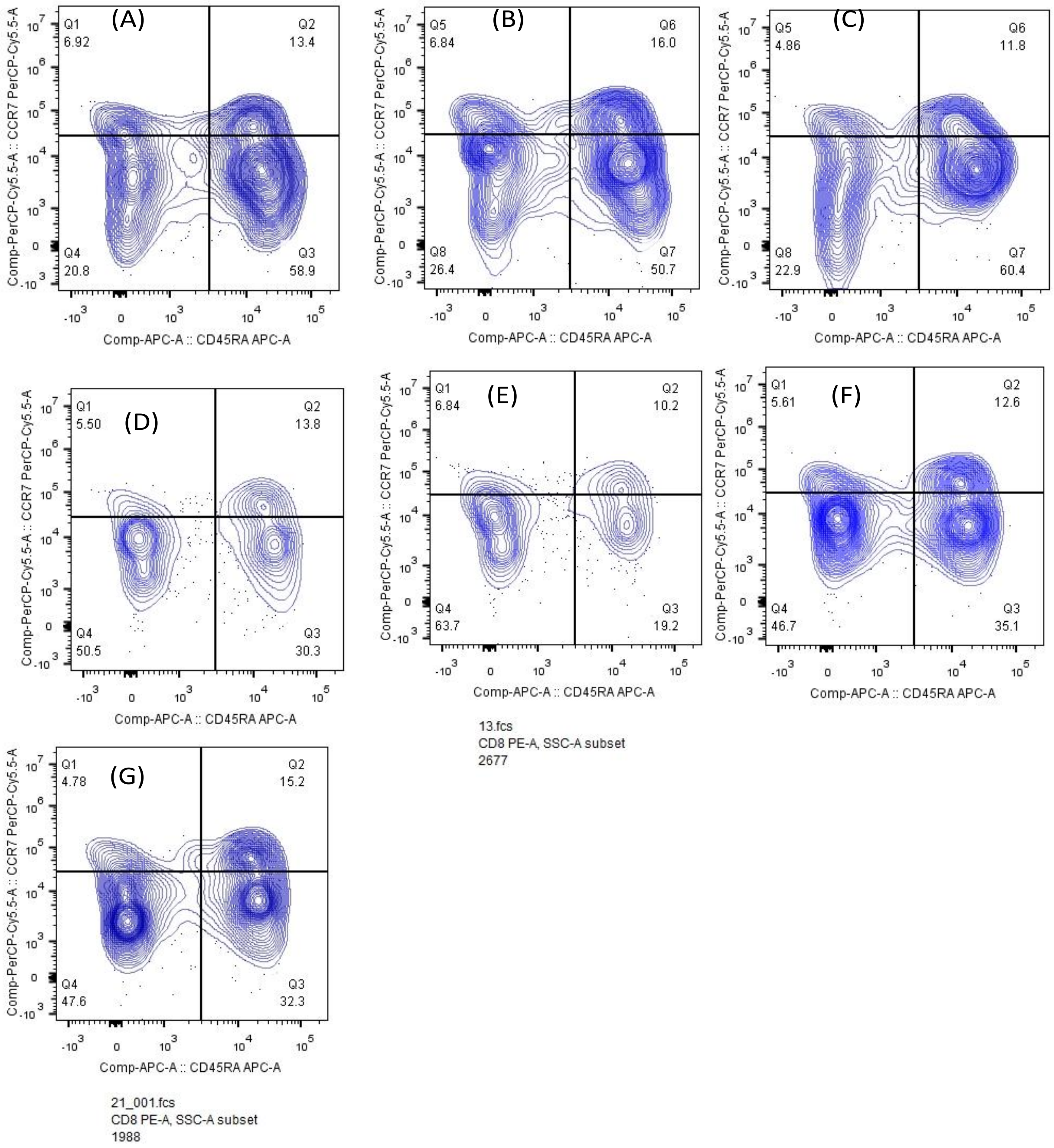

Figure S7. Up-regulation of effector memory CD8+T cells in tumor micro-environment post-treatment. Only cell (A), activated cell (B), control cell in tumor micro-environment (C), for **2i** (D), **3i** (E), **4i** (F), and **4K** (G). The concentration of the compounds was 50 μM for the T cell activity studies.

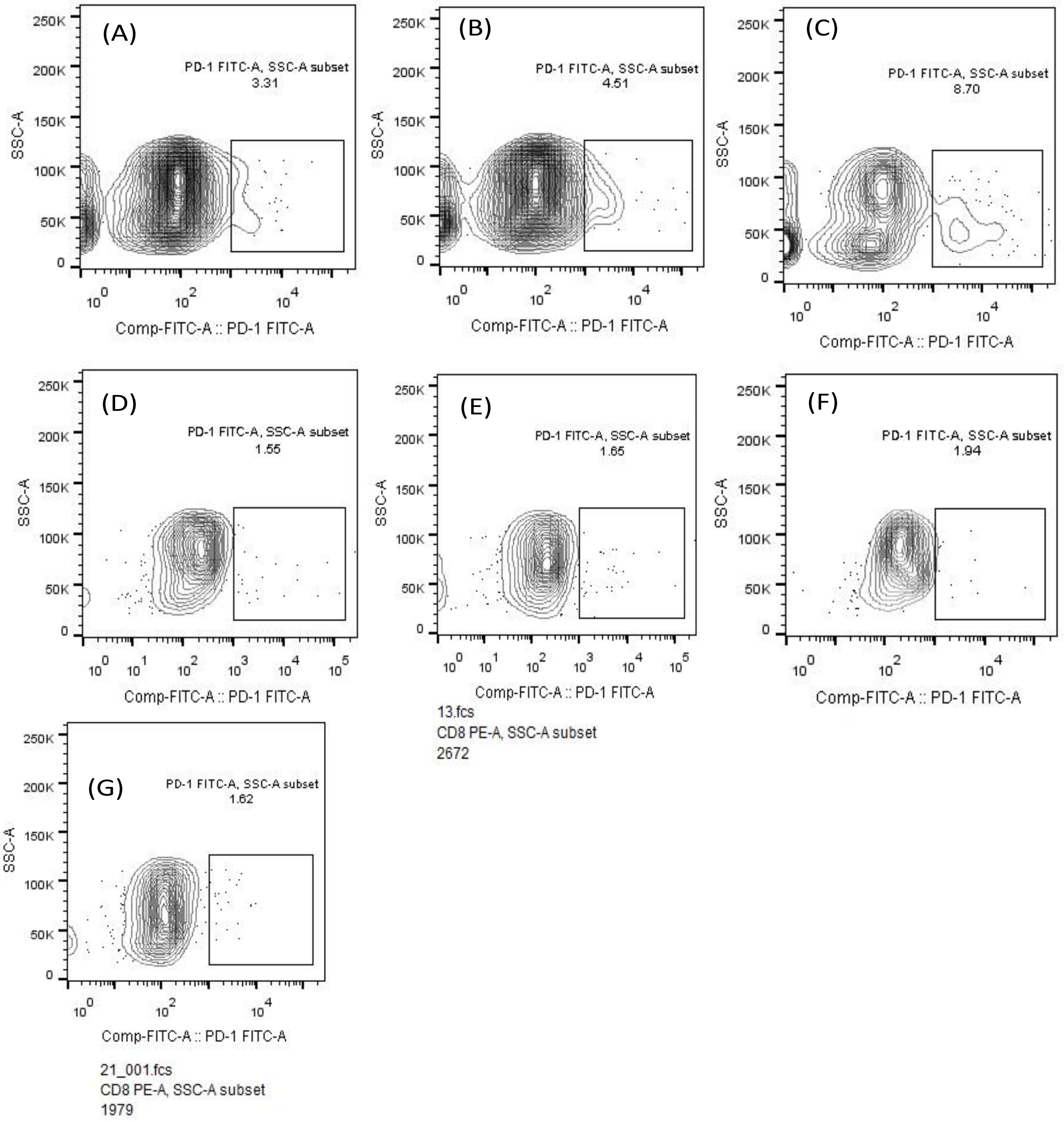

Figure S8. Down-regulation of PD-1 expression on CD8+T cells in tumor micro-environment post-treatment. Only cell (A), activated cell (B), control cell in tumor micro-environment (C), for **2i** (D), **3i** (E), **4i** (F), and **4K** (G). The concentration of the compounds was 50 μM for the T cell activity studies.

Flowcytometry analysis for % population of CD8 T cell in *in vivo* solid tumor:

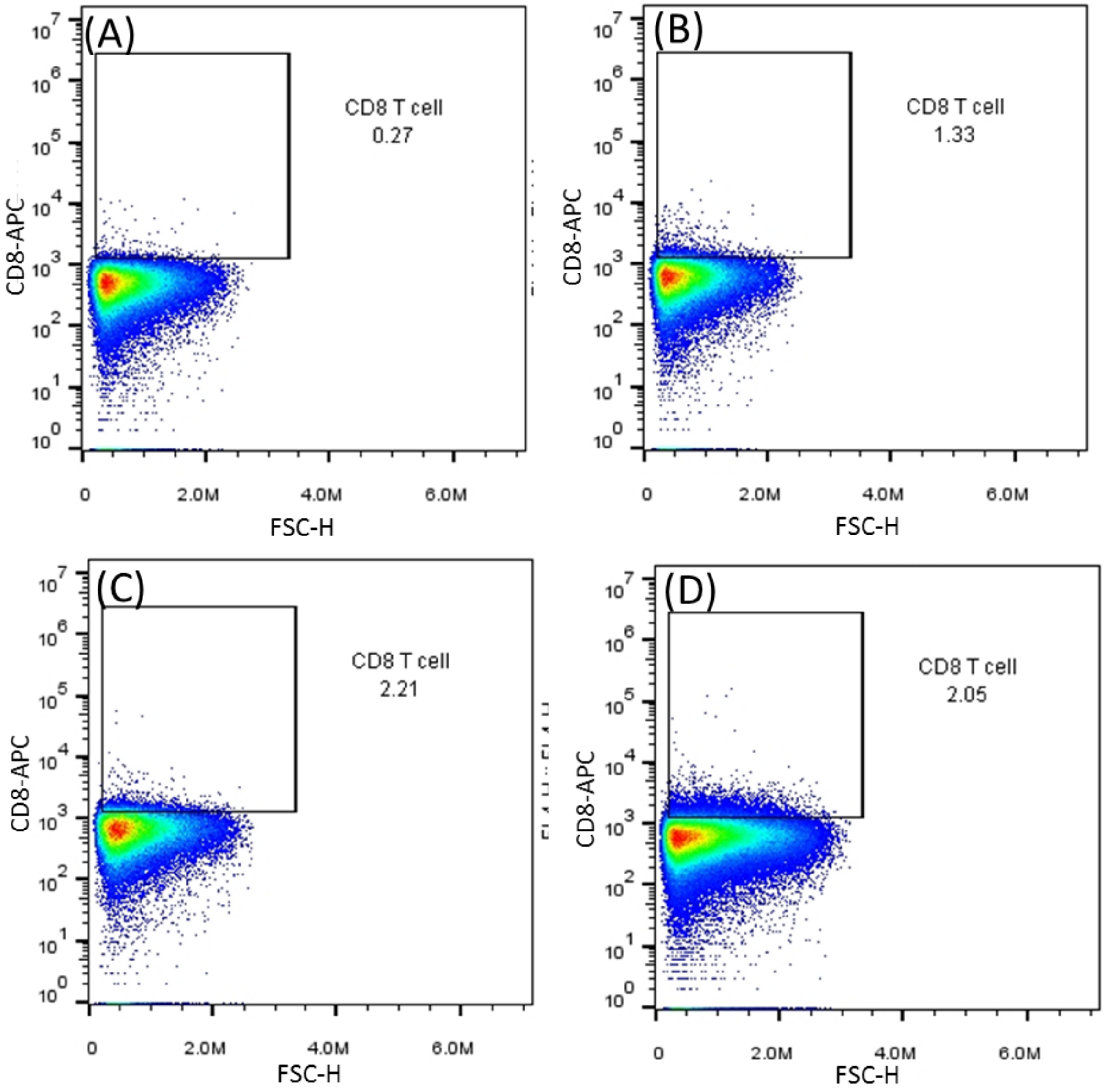

Figure S9. IDO1 inhibition induces CD8+ T cell population in *vivo* solid tumors. Control (A), **3i** (B), **4i** (C), and **4K** (D).

^{1}H and ^{13}C NMR spectra of synthesized compounds:

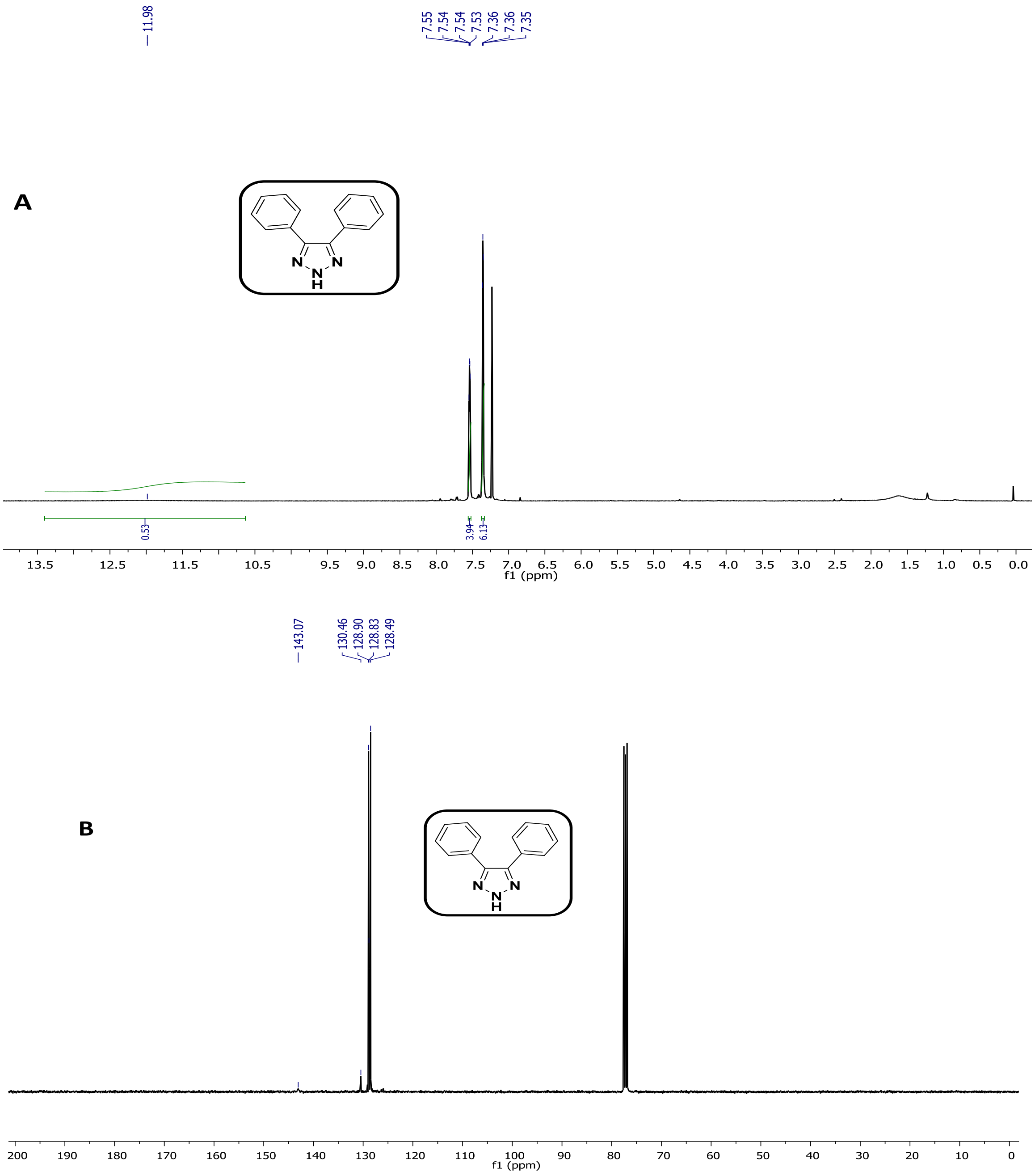

Figure S9. ^{1}H (A) and ^{13}C (B) NMR of compound **1a**.

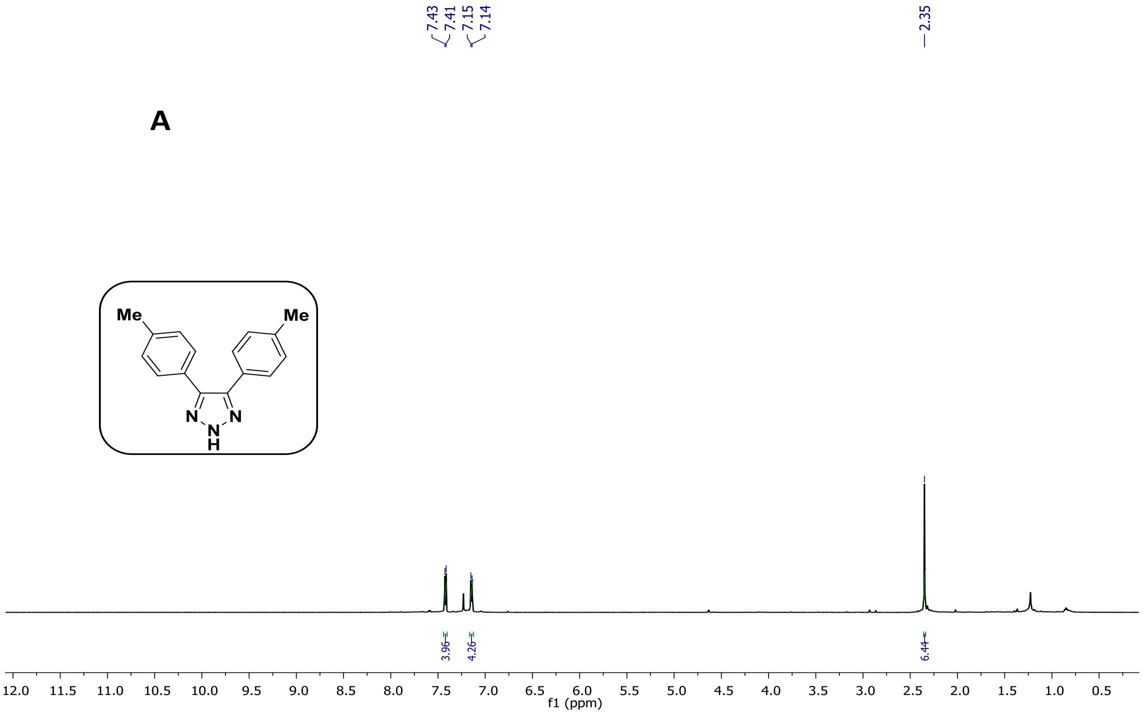

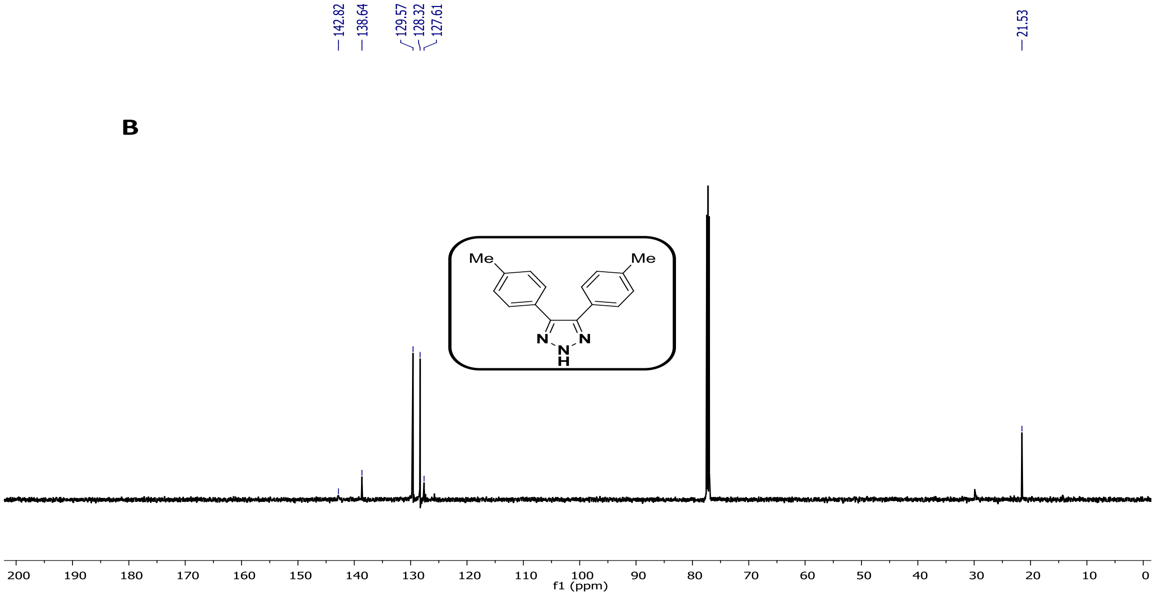

Figure S10. ^{1}H (A) and ^{13}C (B) NMR of compound **1b**.

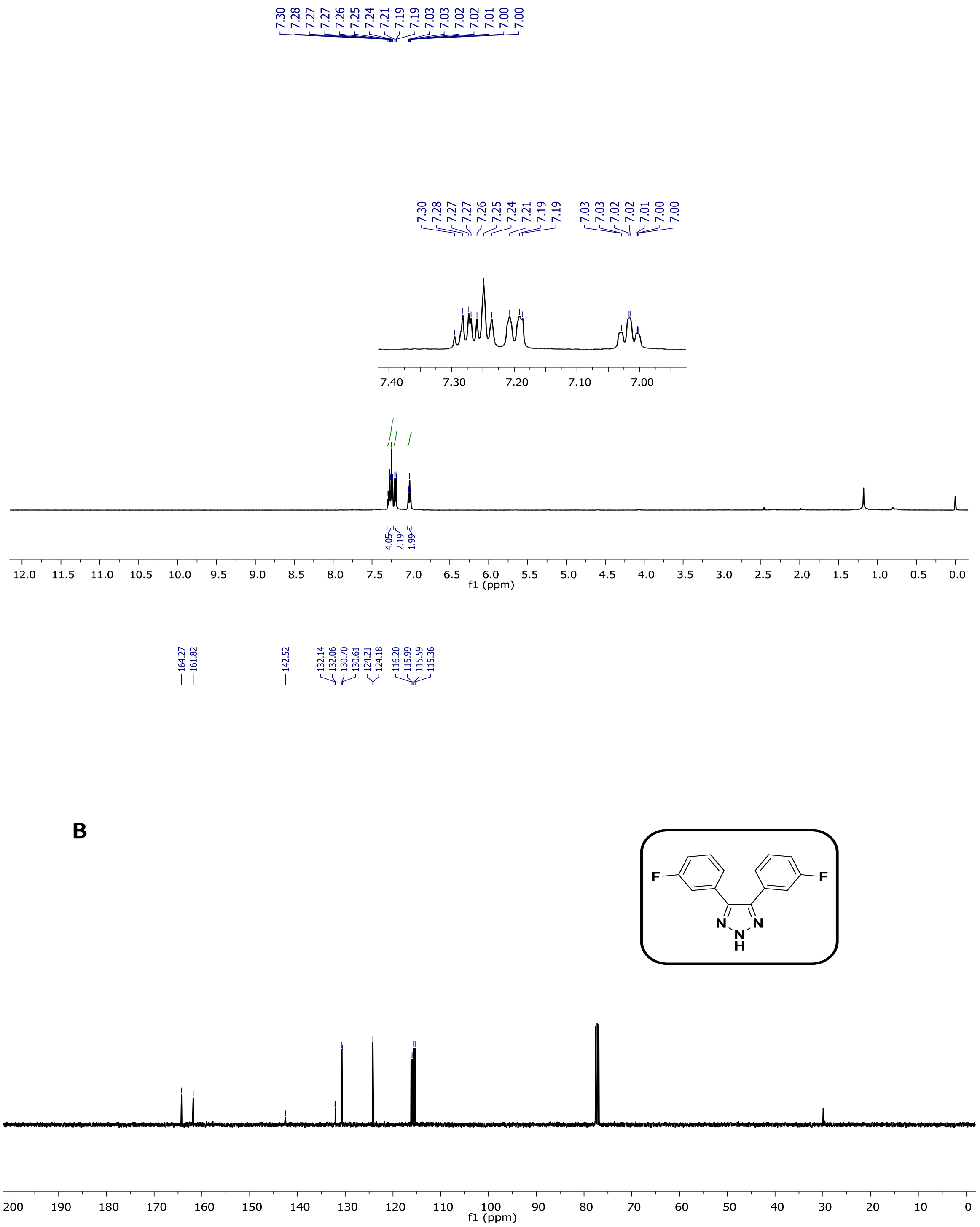

Figure S11. ^{1}H (A) and ^{13}C (B) NMR of compound **1c**.

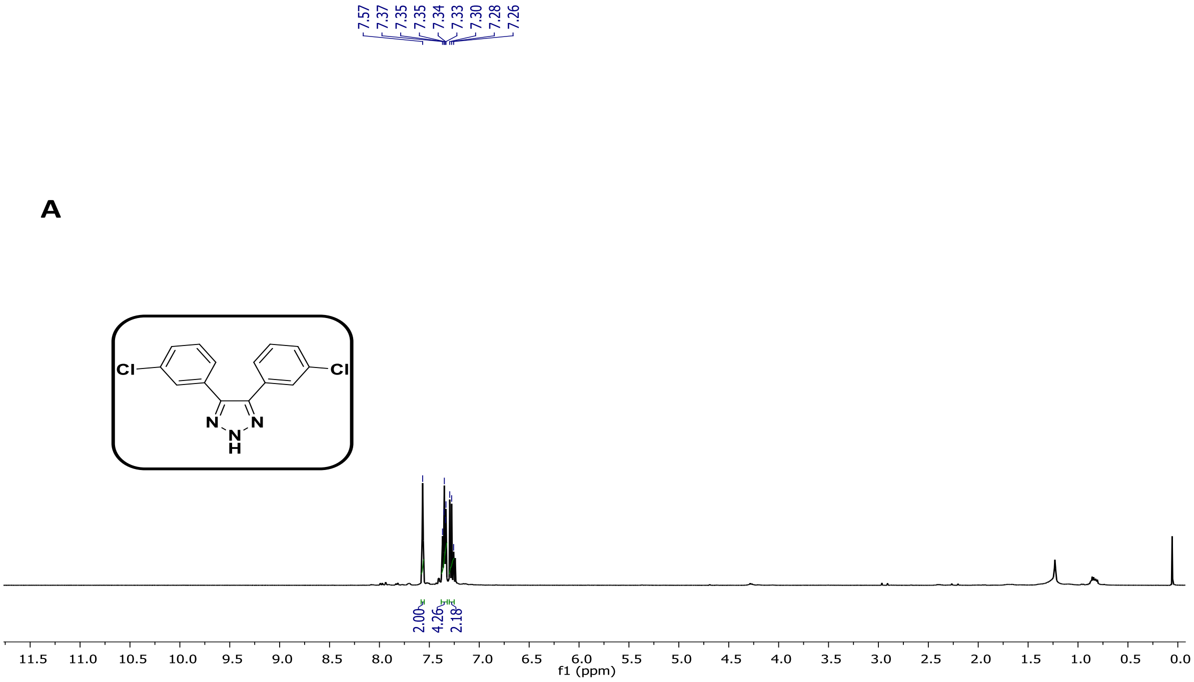

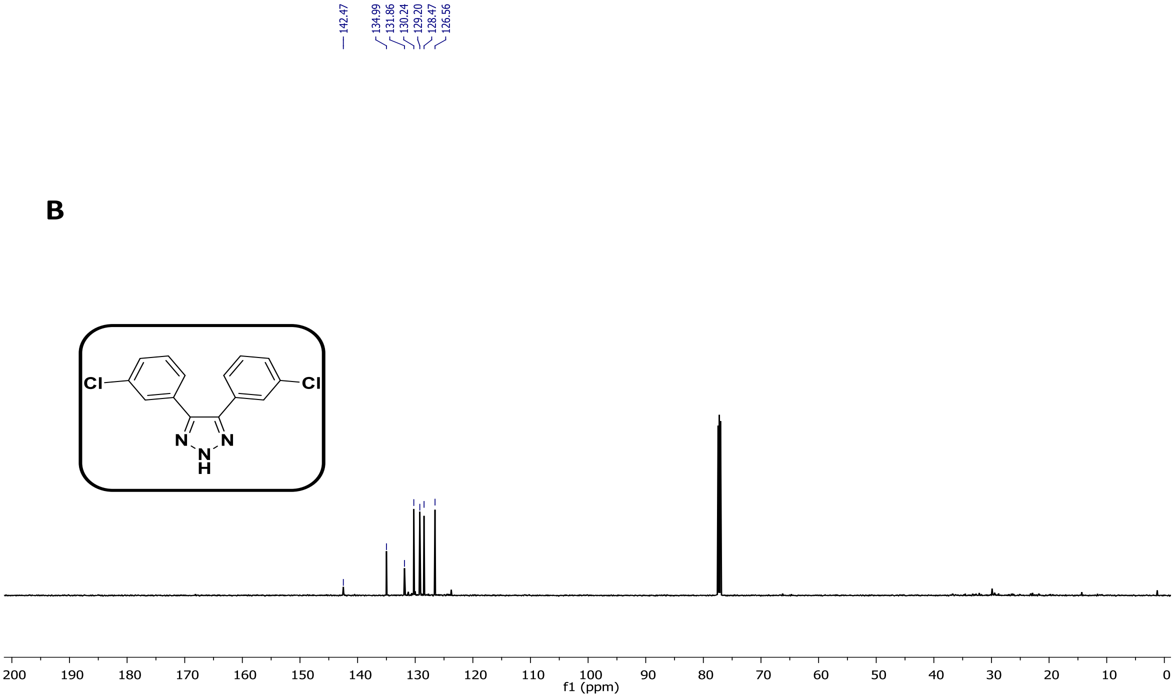

Figure S12. ^{1}H (A) and ^{13}C (B) NMR of compound **1d**.

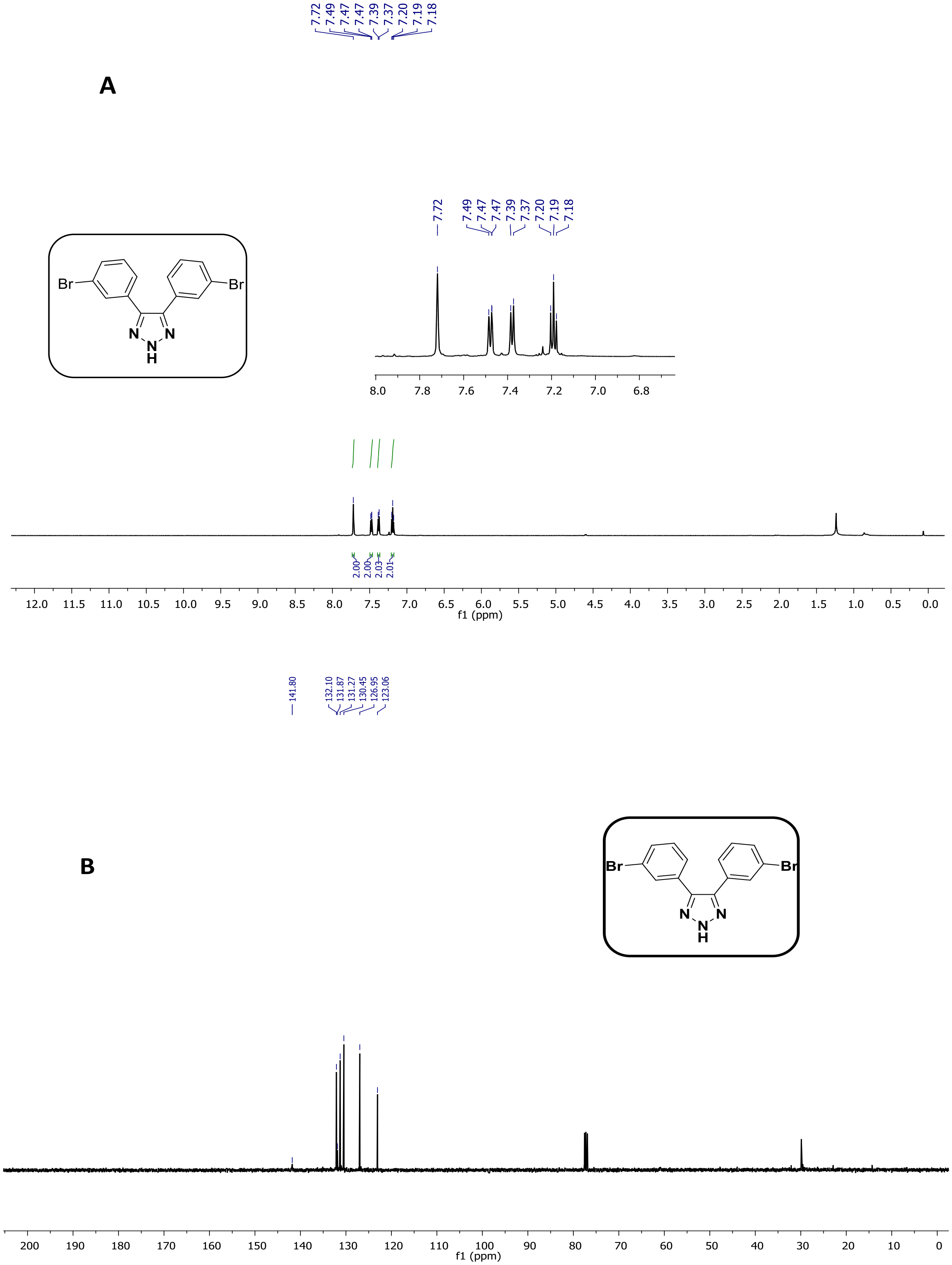

Figure S13. ^{1}H (A) and ^{13}C (B) NMR of compound **1e**.

A

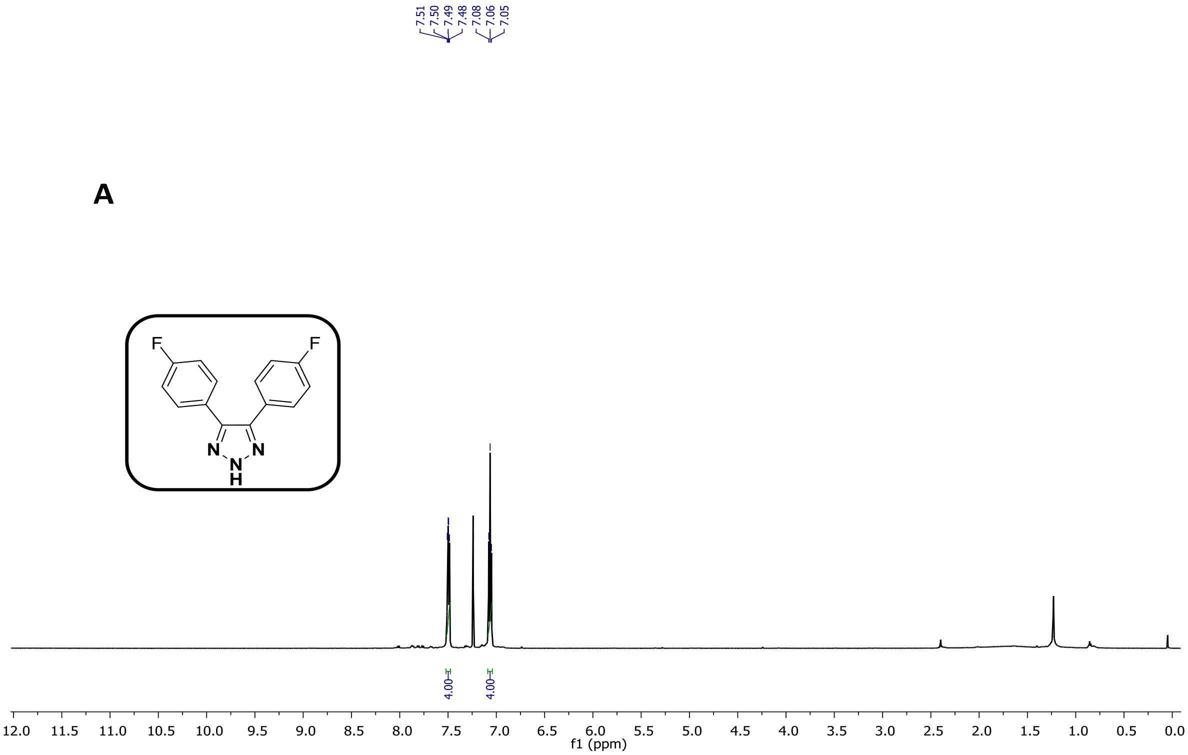

B

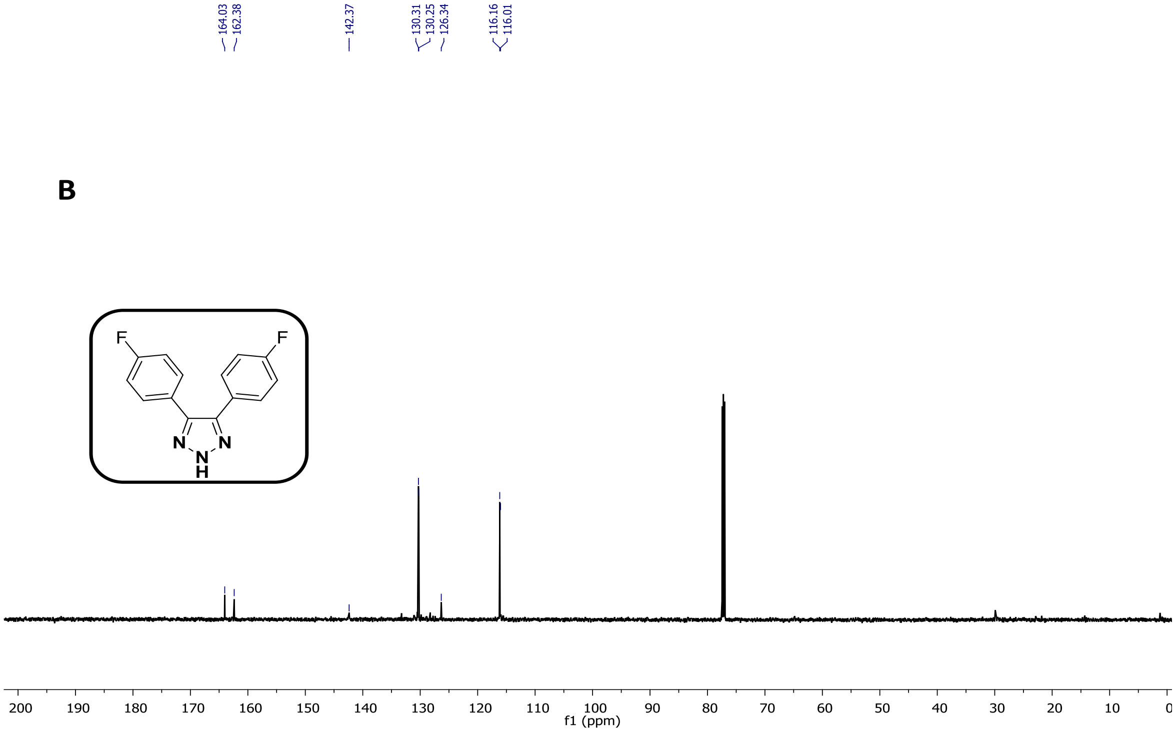

Figure S14. ^{1}H (A) and ^{13}C (B) NMR of compound **1f**.

A

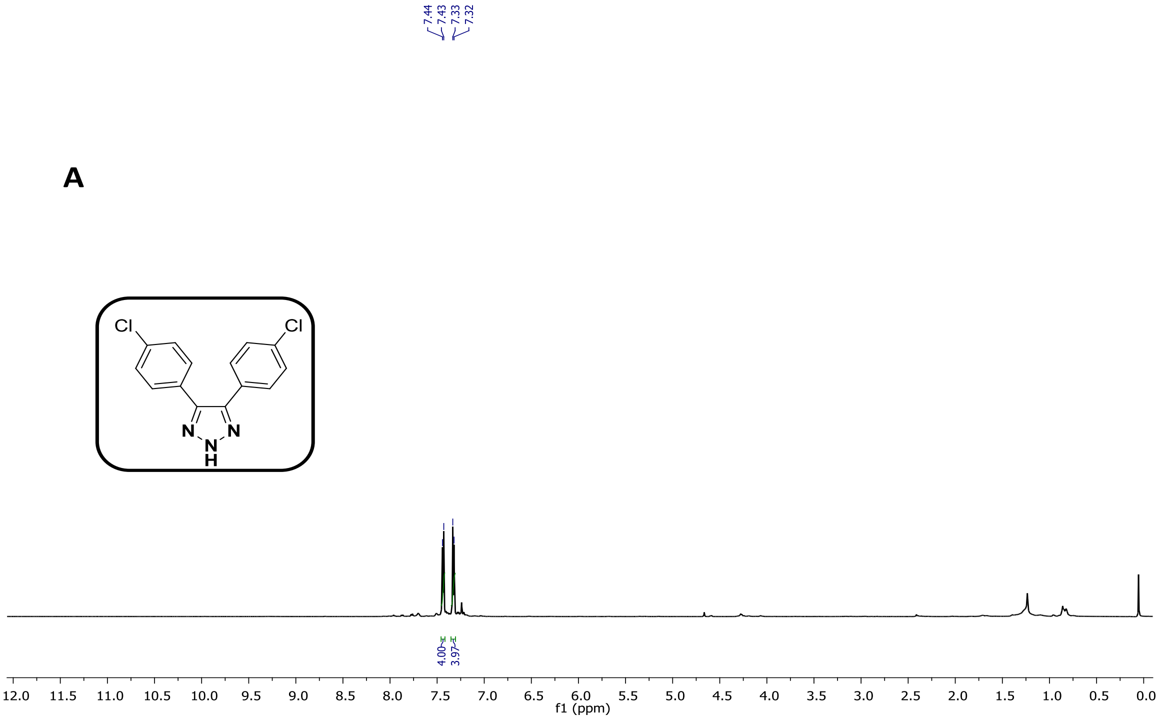

B

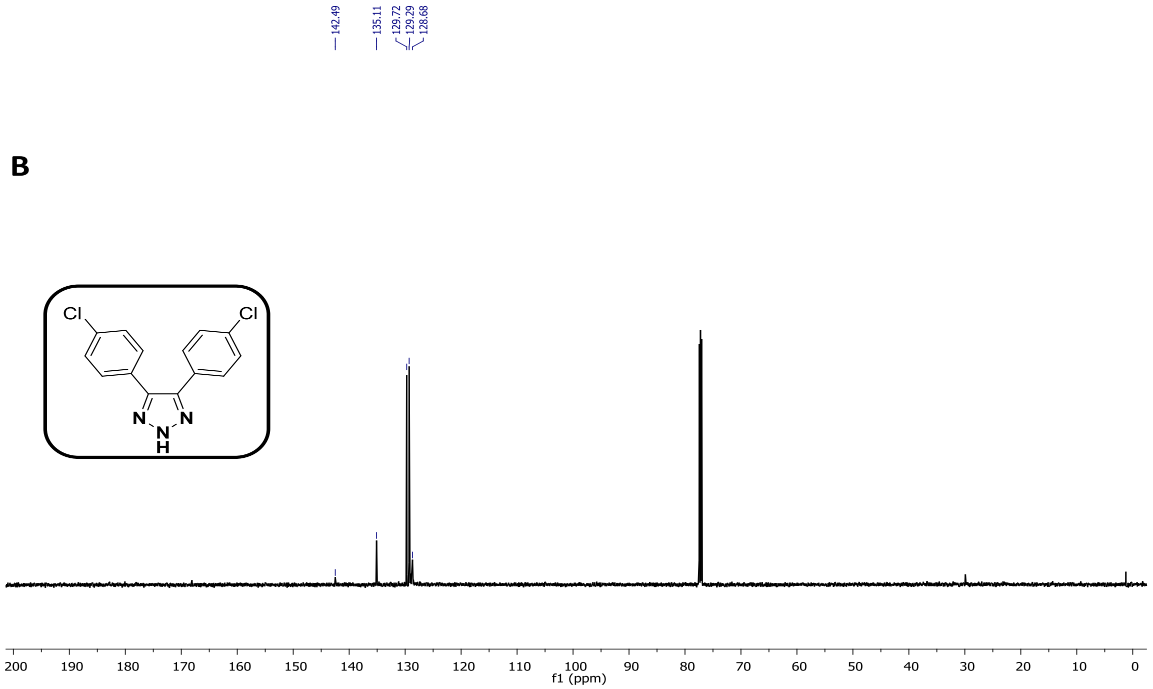

Figure S15. ^{1}H (A) and ^{13}C (B) NMR of compound **1g**.

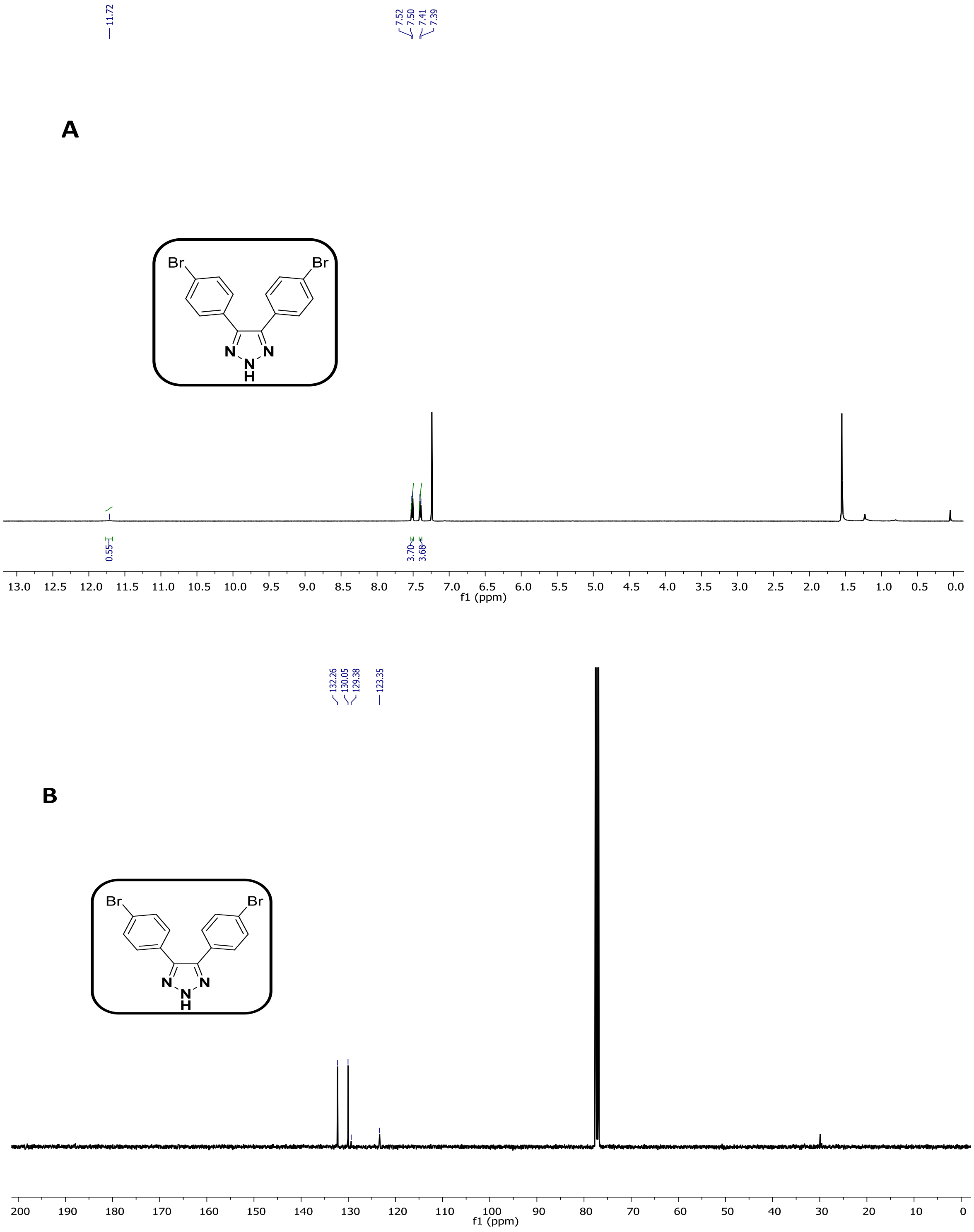

Figure S16. ^{1}H (A) and ^{13}C (B) NMR of compound **1h**.

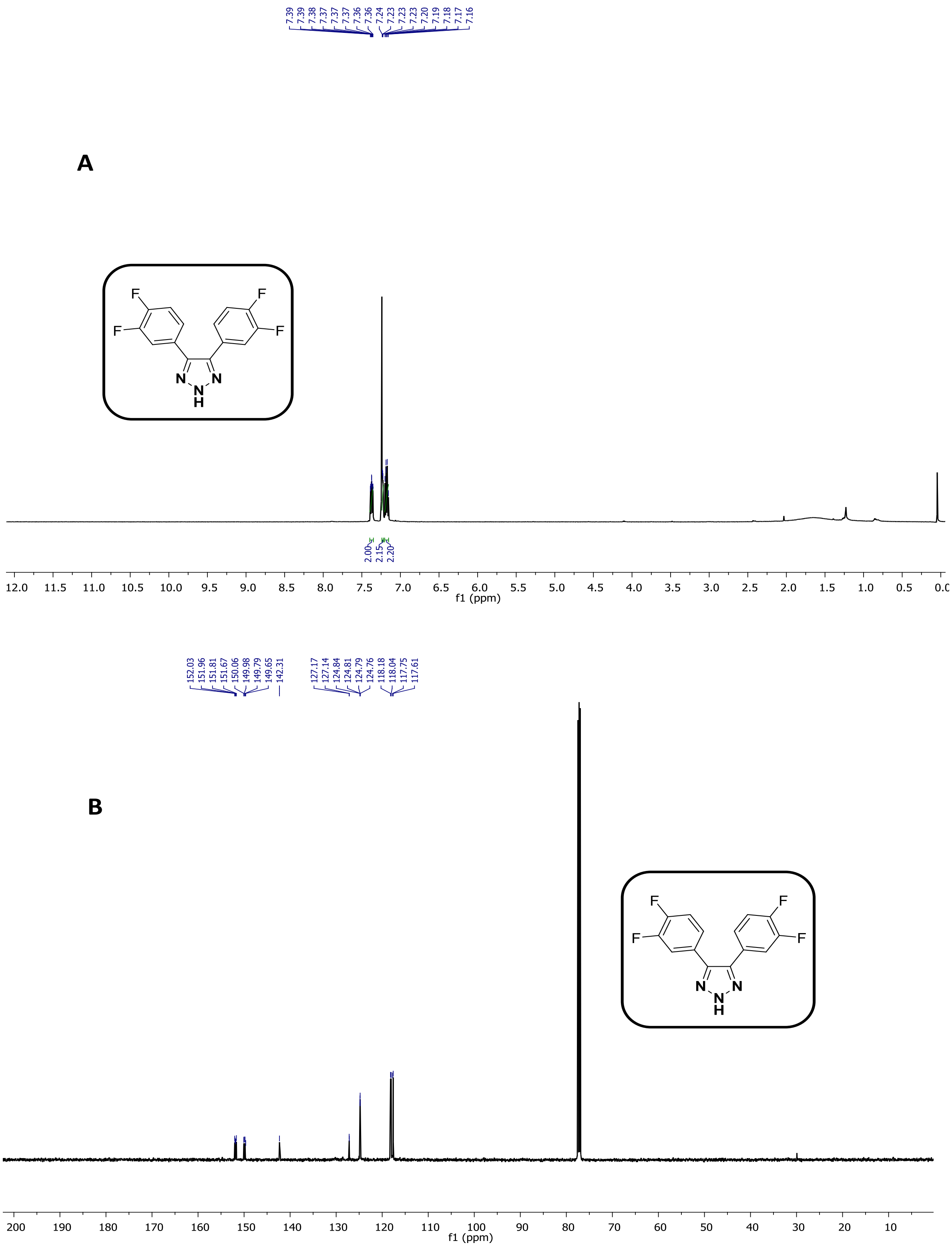

Figure S17. ^{1}H (A) and ^{13}C (B) NMR of compound **1i**.

A

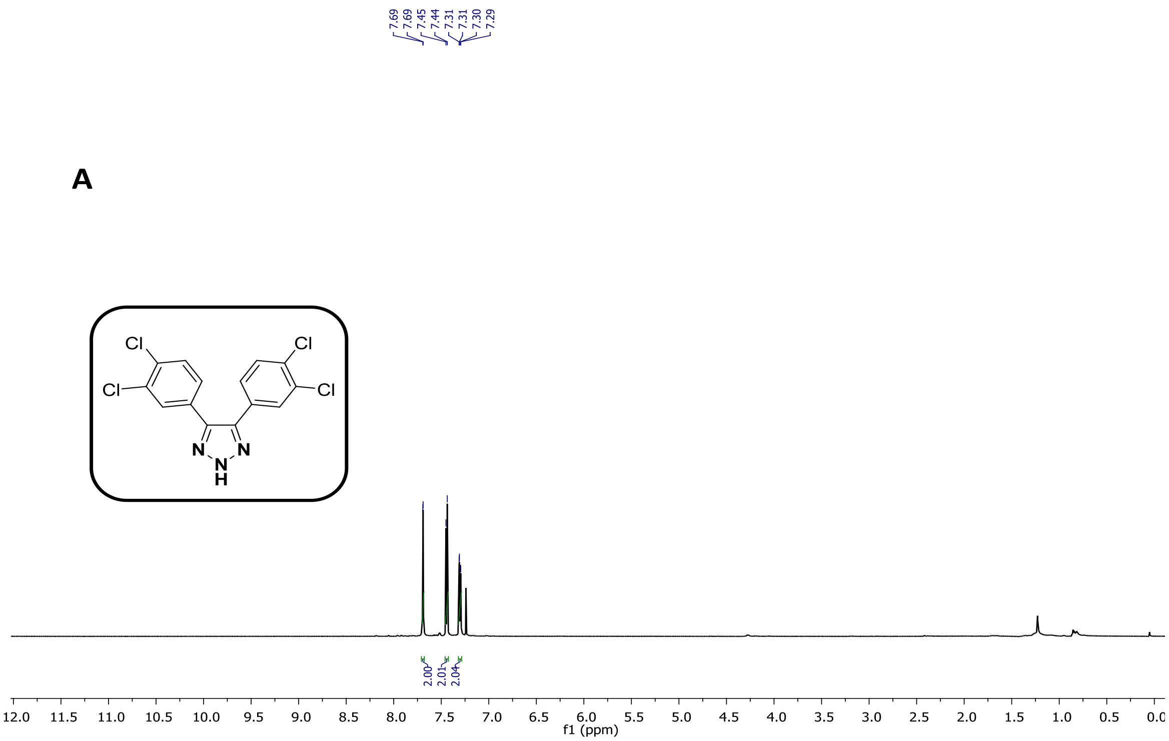

B

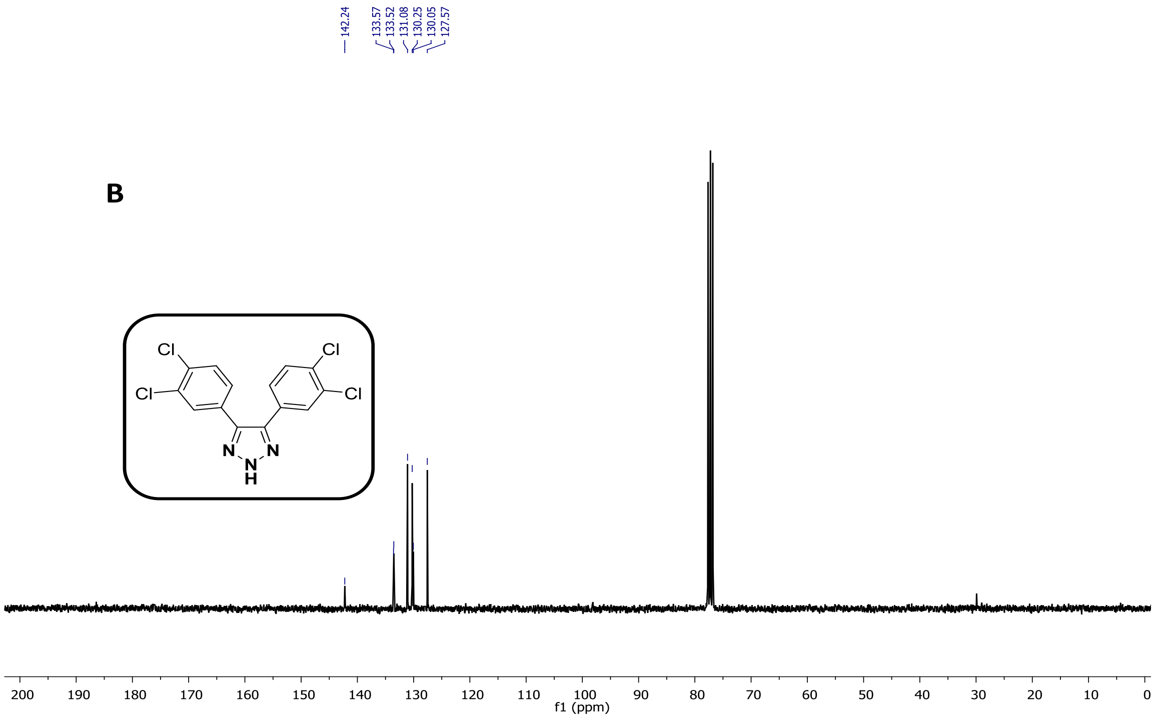

Figure S18. ^{1}H (A) and ^{13}C (B) NMR of compound **1j**.

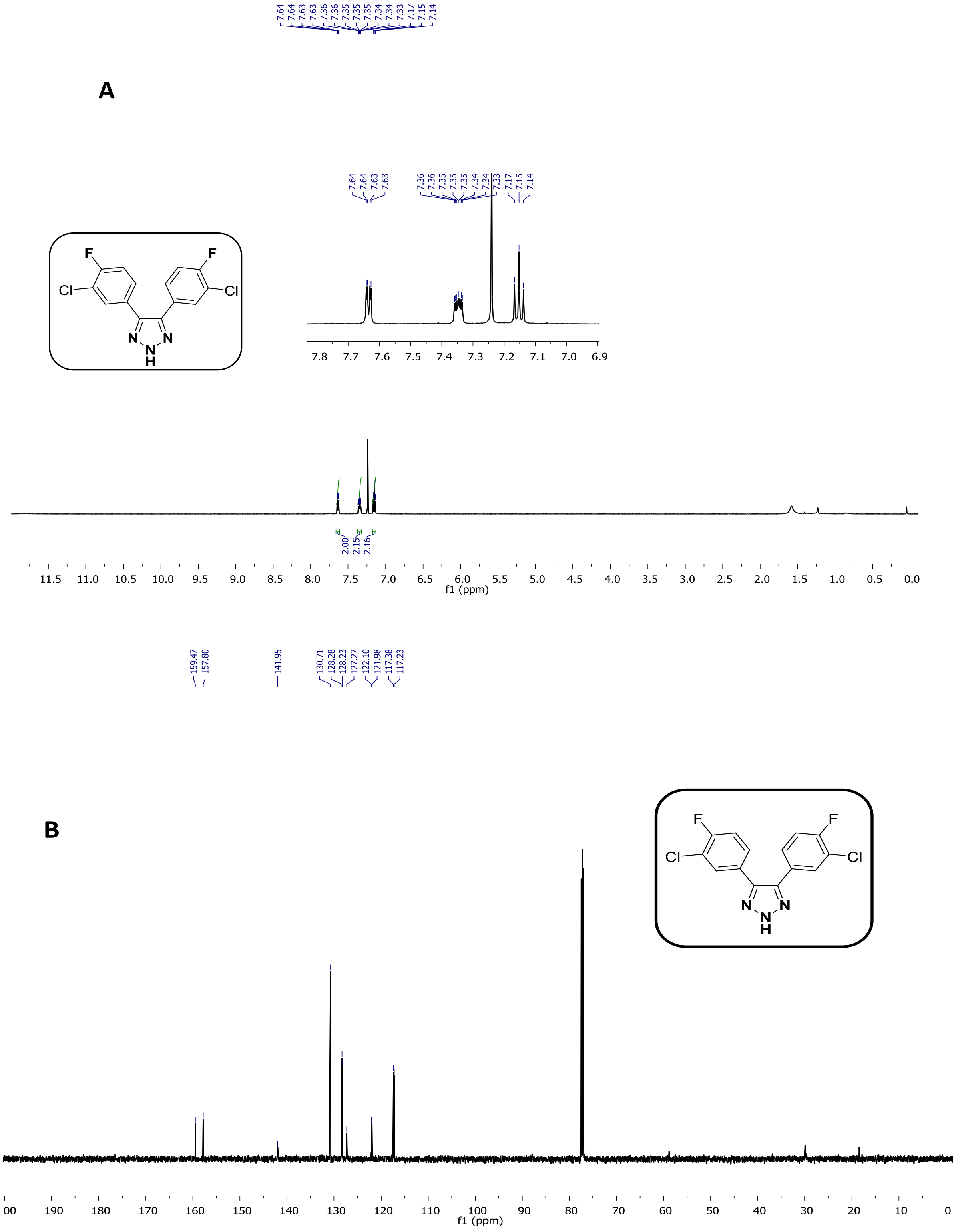

Figure S19. ^{1}H (A) and ^{13}C (B) NMR of compound **1k**.

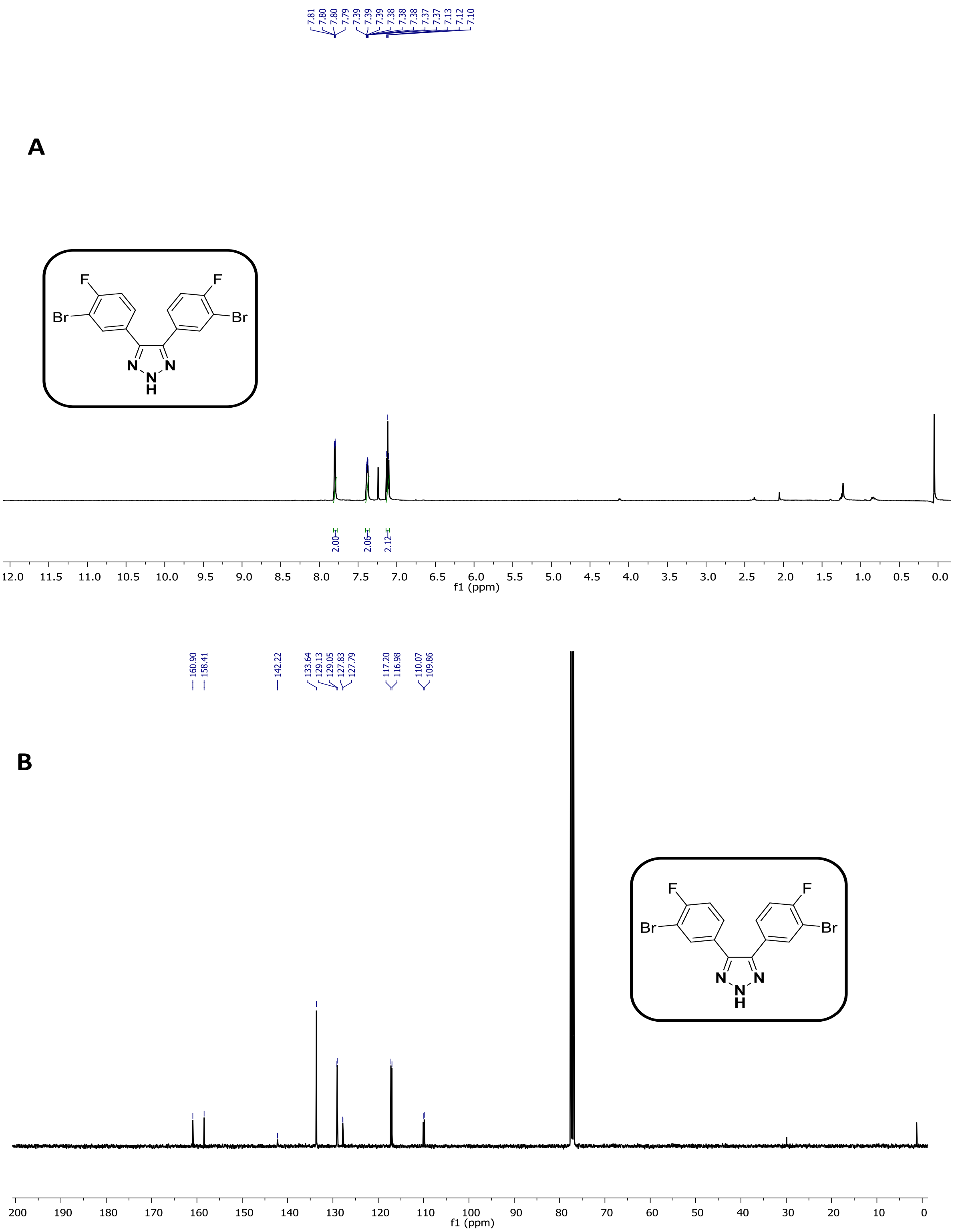

Figure S20. ^{1}H (A) and ^{13}C (B) NMR of compound **1l**.

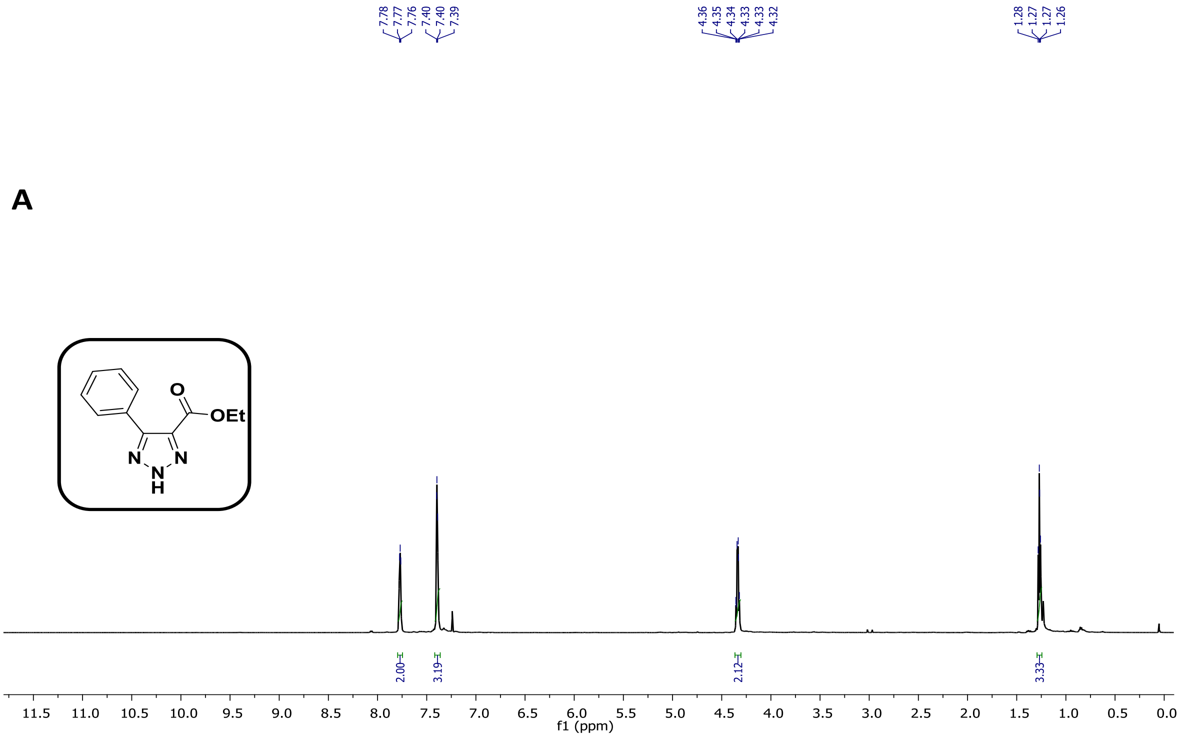

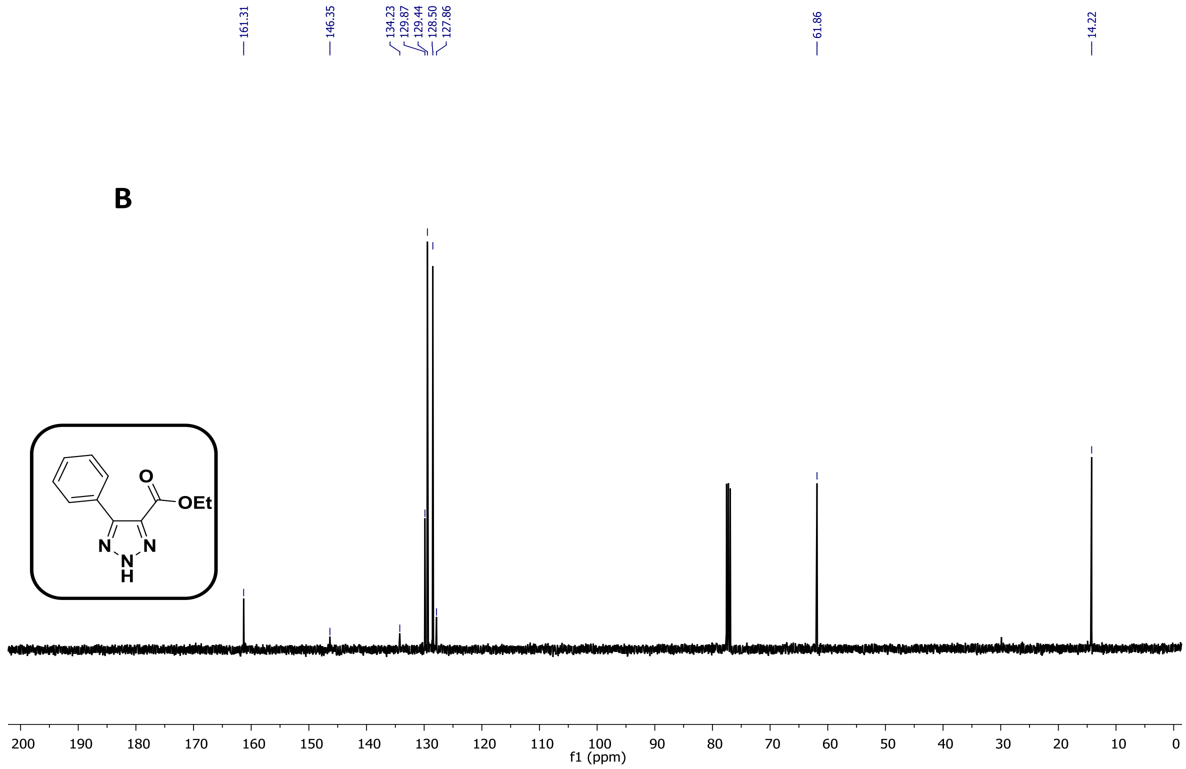

Figure S21. ^{1}H (A) and ^{13}C (B) NMR of compound **2a**.

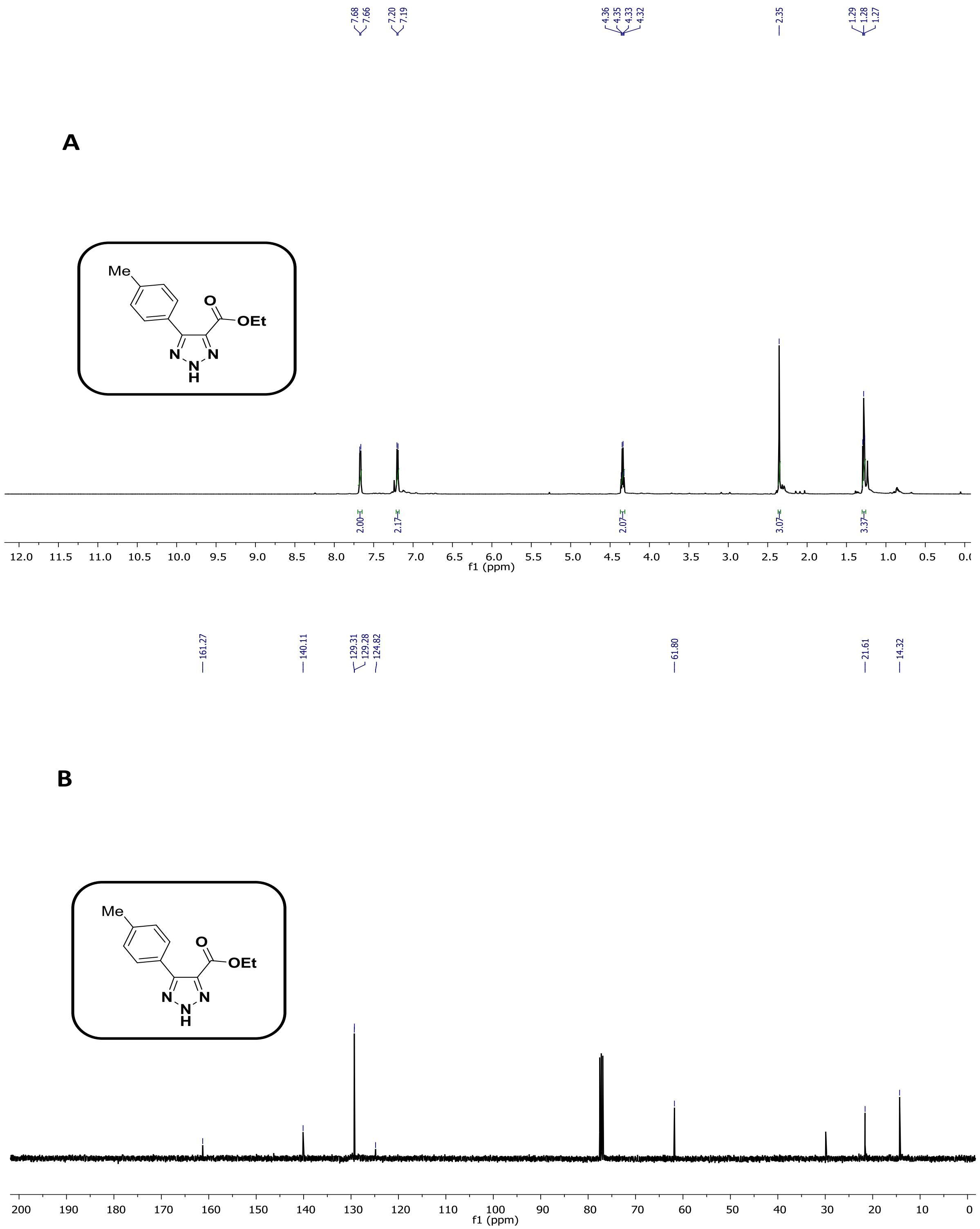

Figure S22. ^{1}H (A) and ^{13}C (B) NMR of compound **2b**.

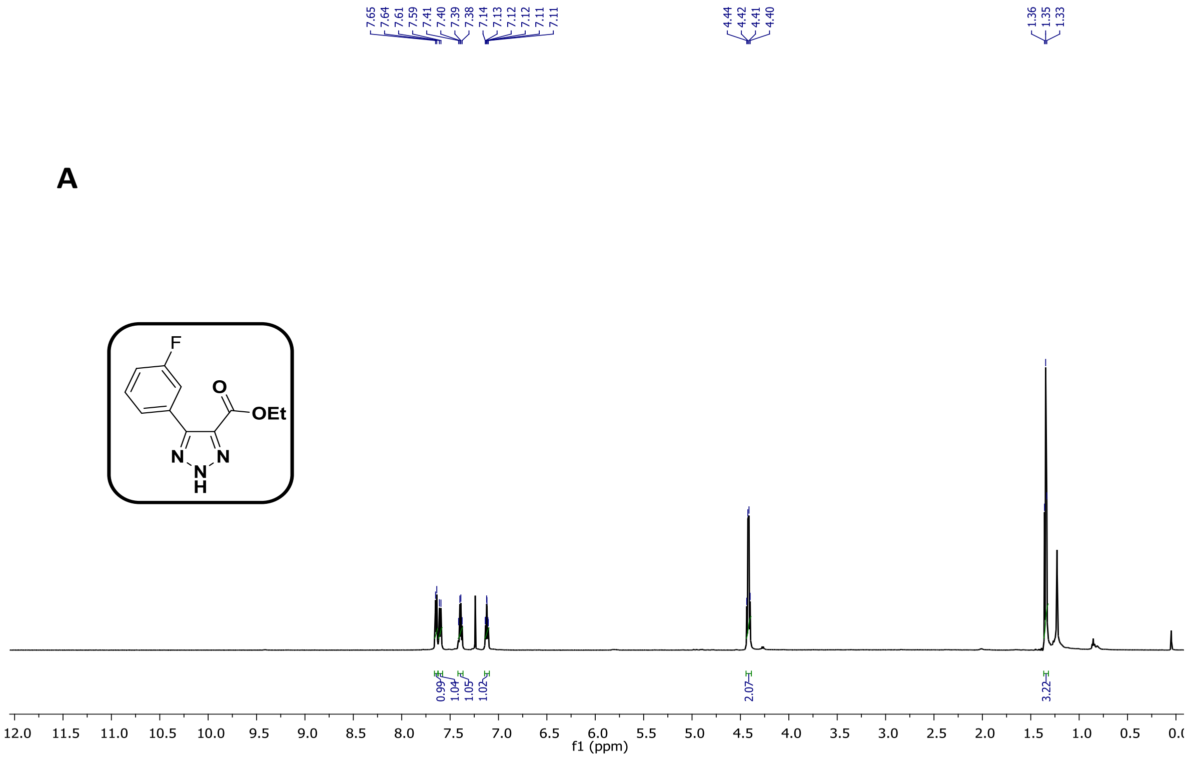

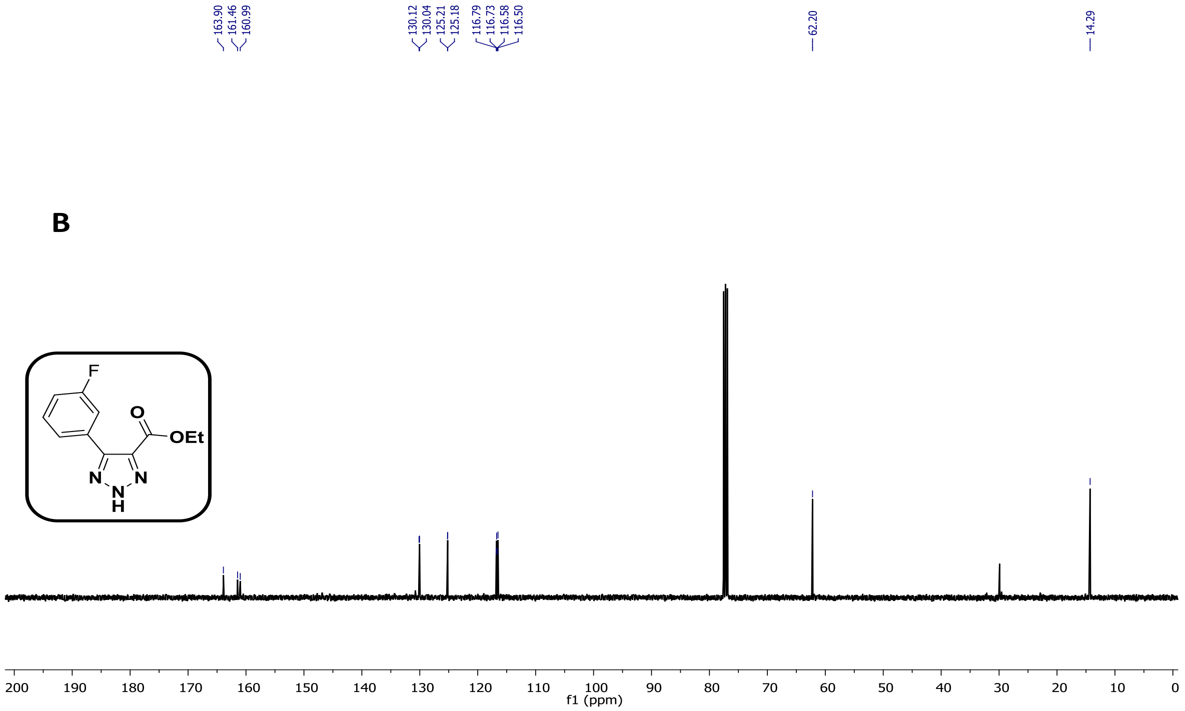

Figure S23. ^{1}H (A) and ^{13}C (B) NMR of compound **2c**.

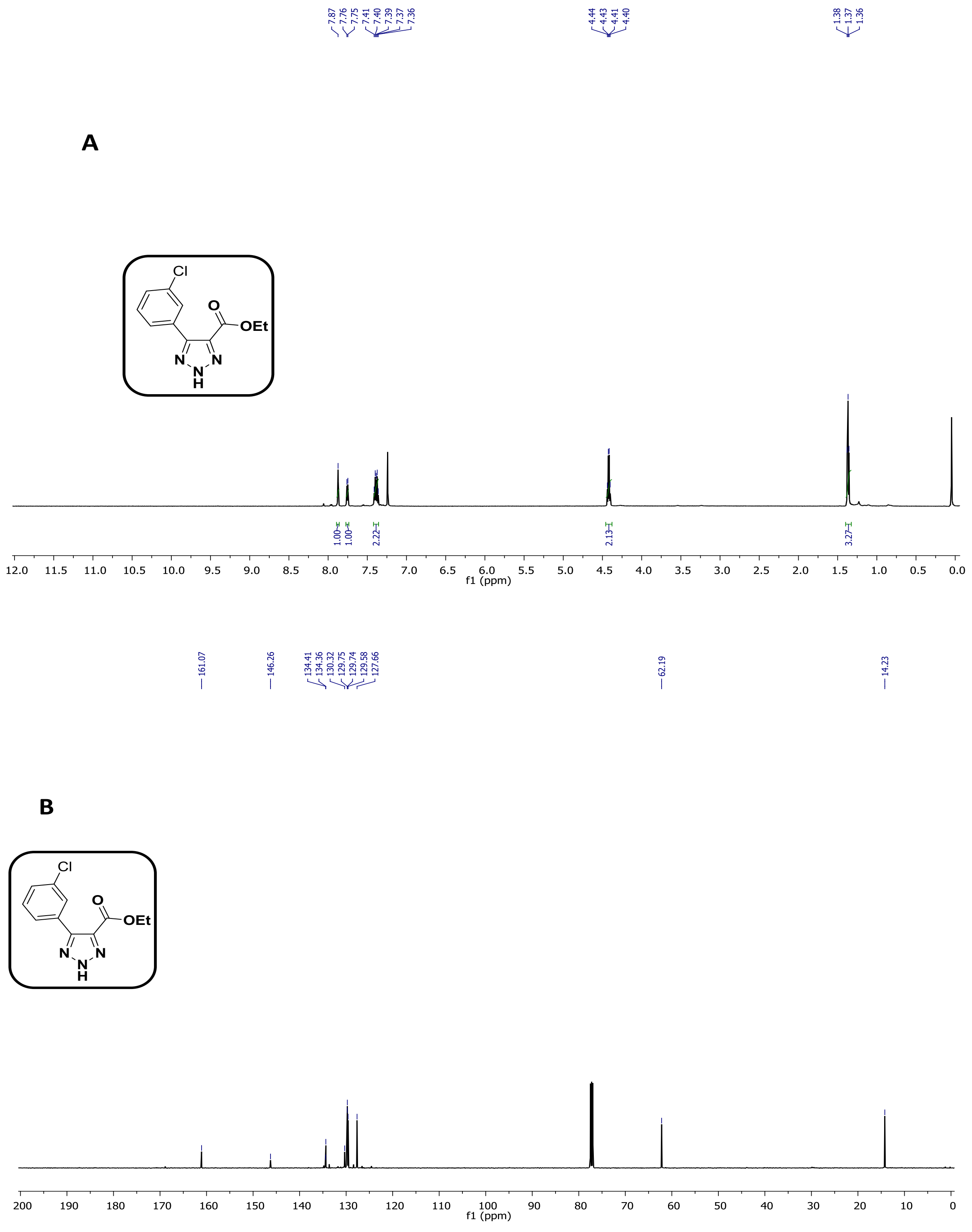

Figure S24. ^{1}H (A) and ^{13}C (B) NMR of compound **2d**.

A

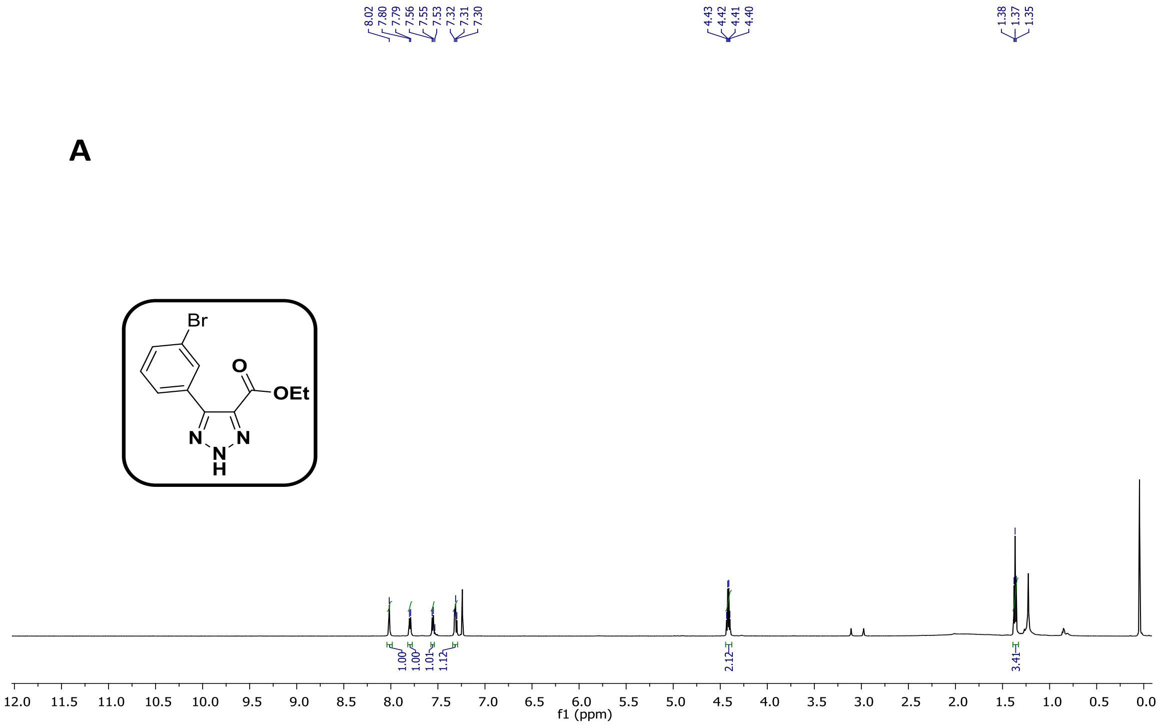

B

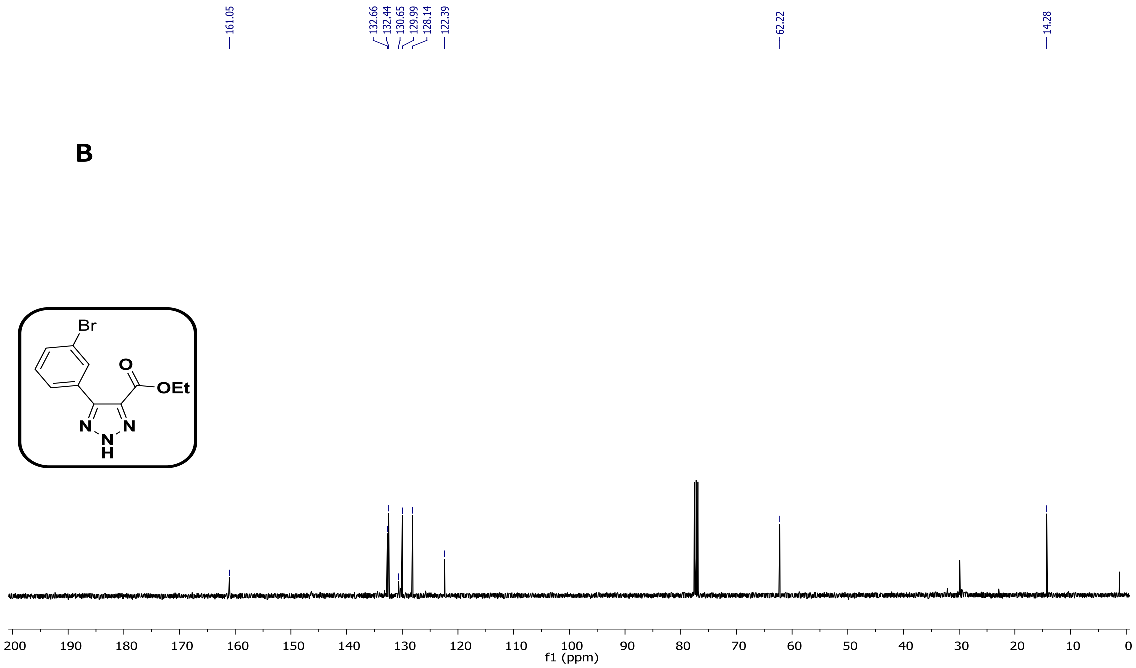

Figure S25. ^{1}H (A) and ^{13}C (B) NMR of compound **2e**.

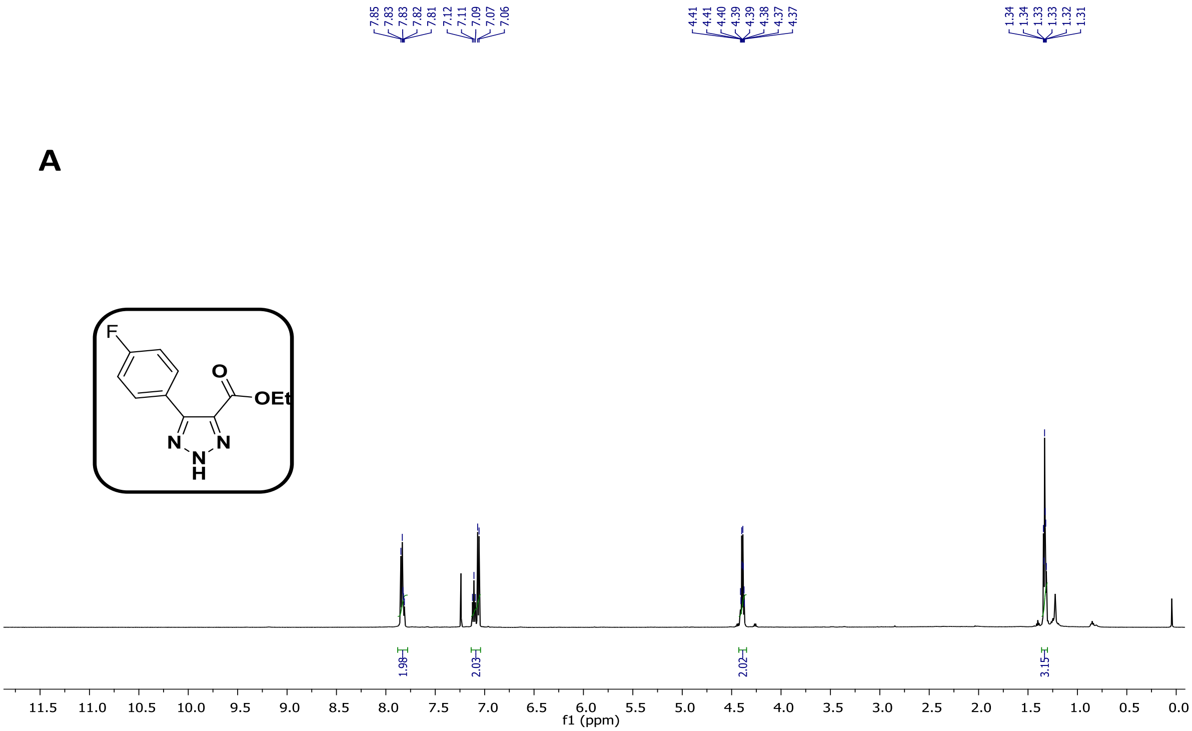

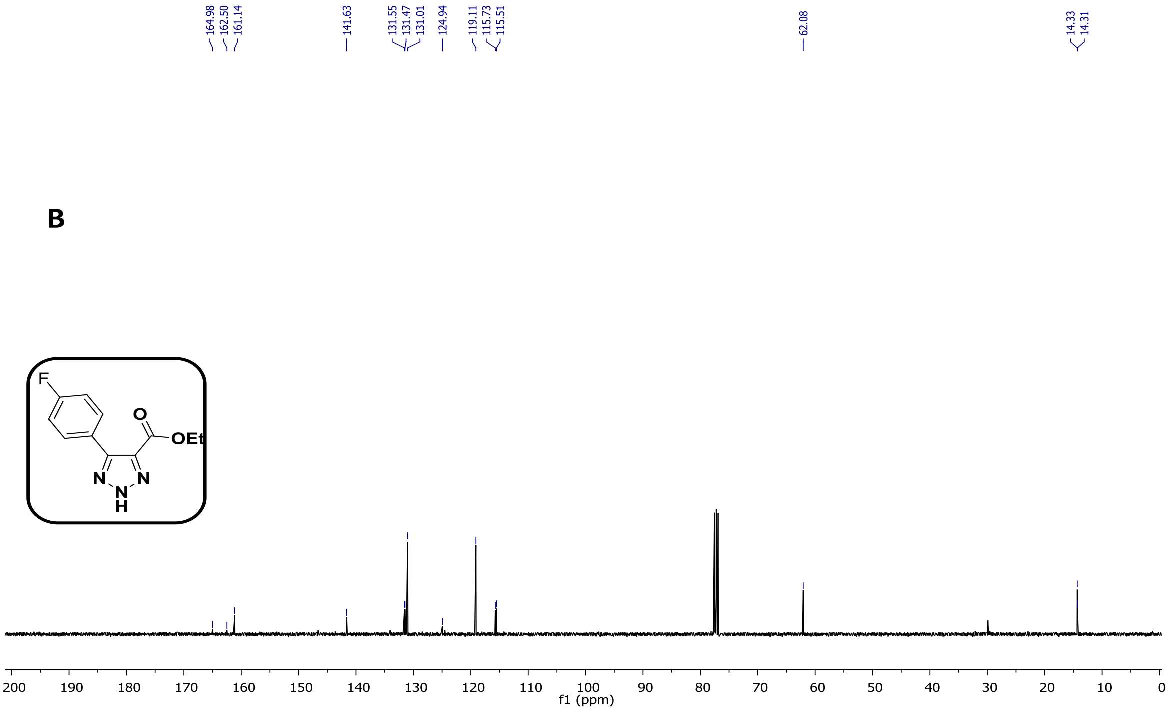

Figure S26. ^{1}H (A) and ^{13}C (B) NMR of compound **2f**.

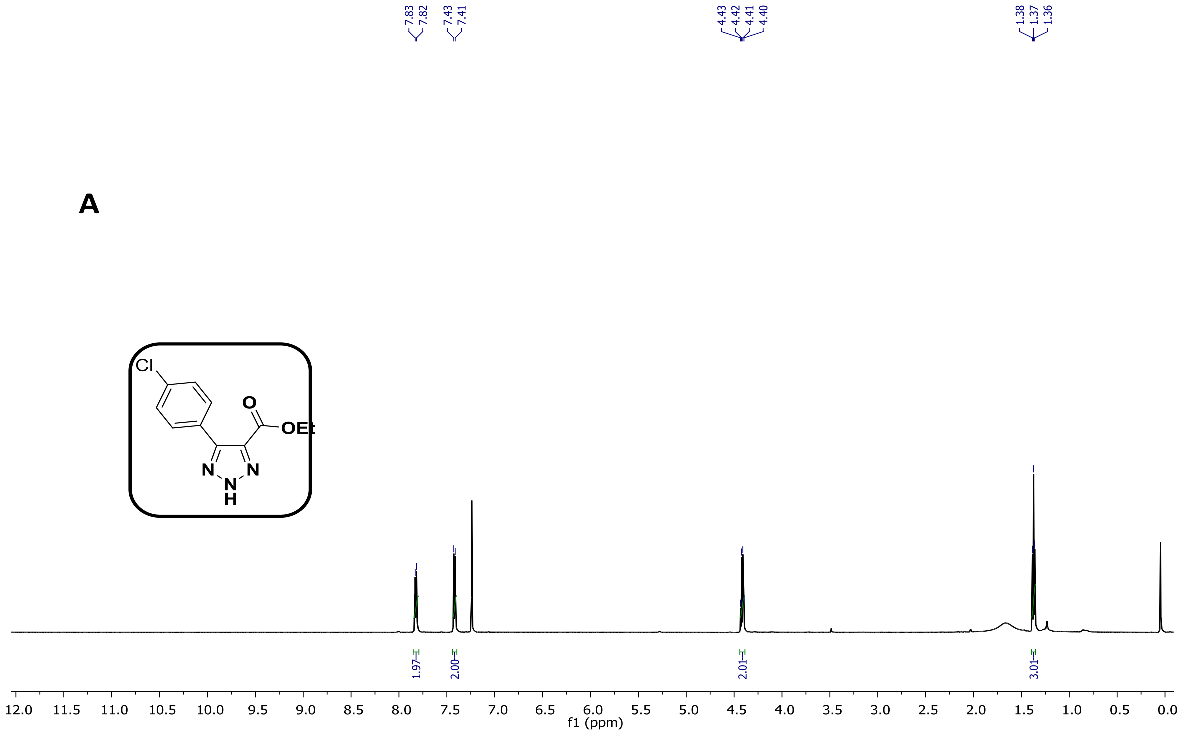

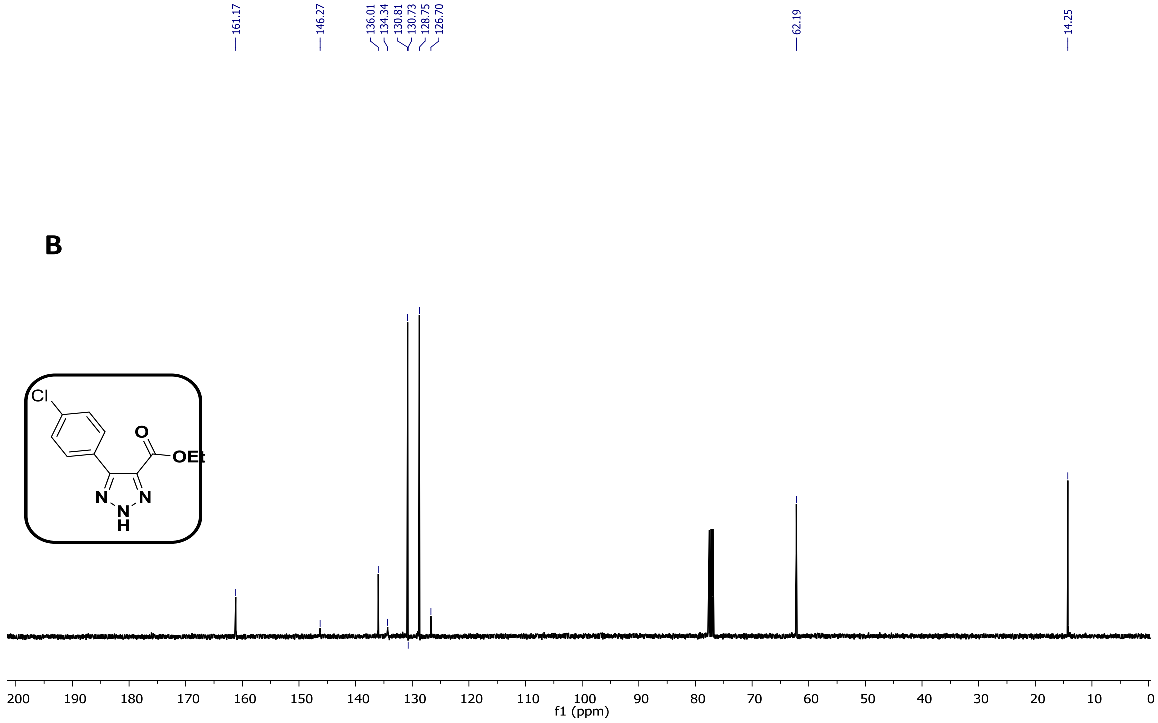

Figure S27. ^{1}H (A) and ^{13}C (B) NMR of compound **2g**.

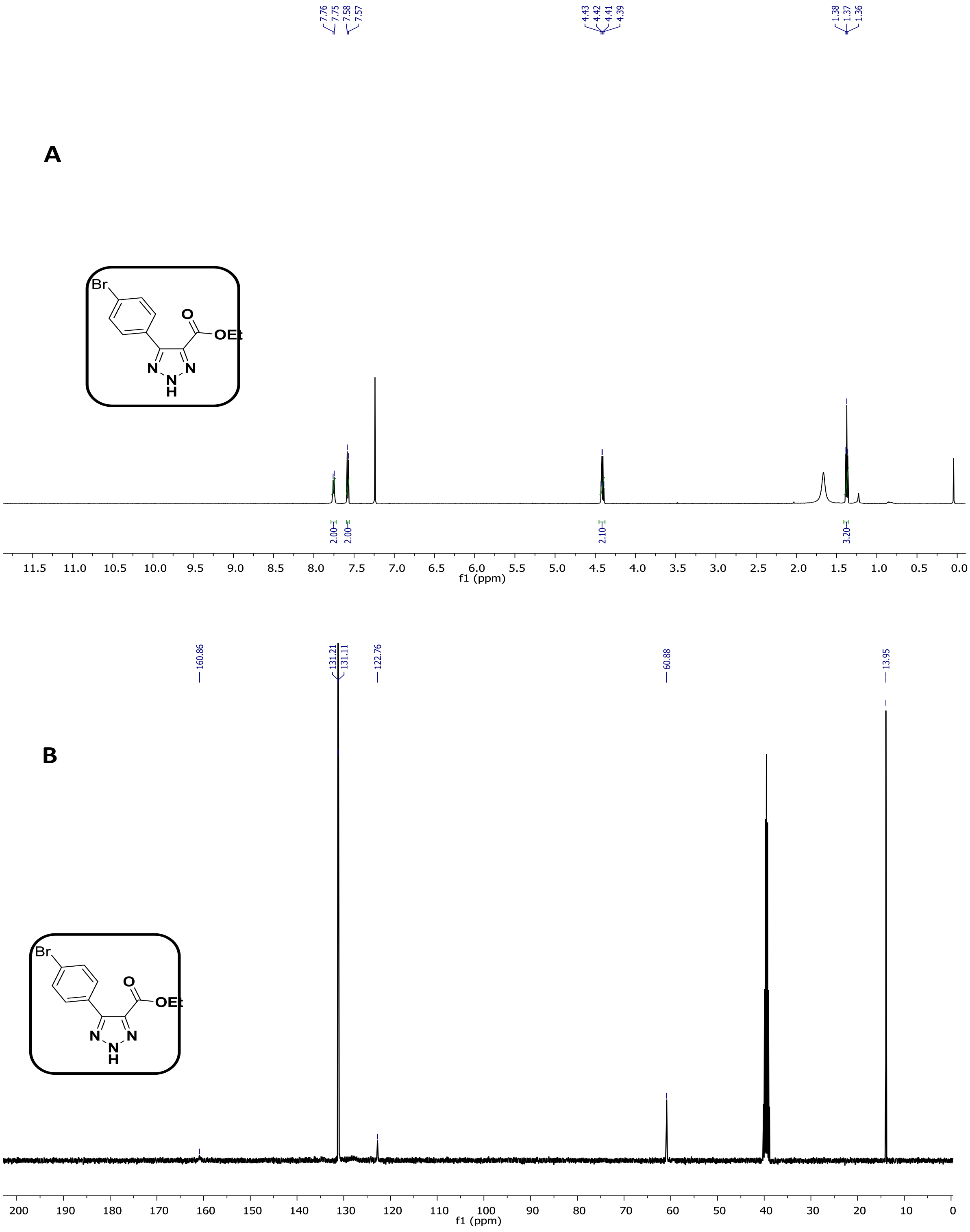

Figure S28. ^{1}H (A) and ^{13}C (B) NMR of compound **2h**.

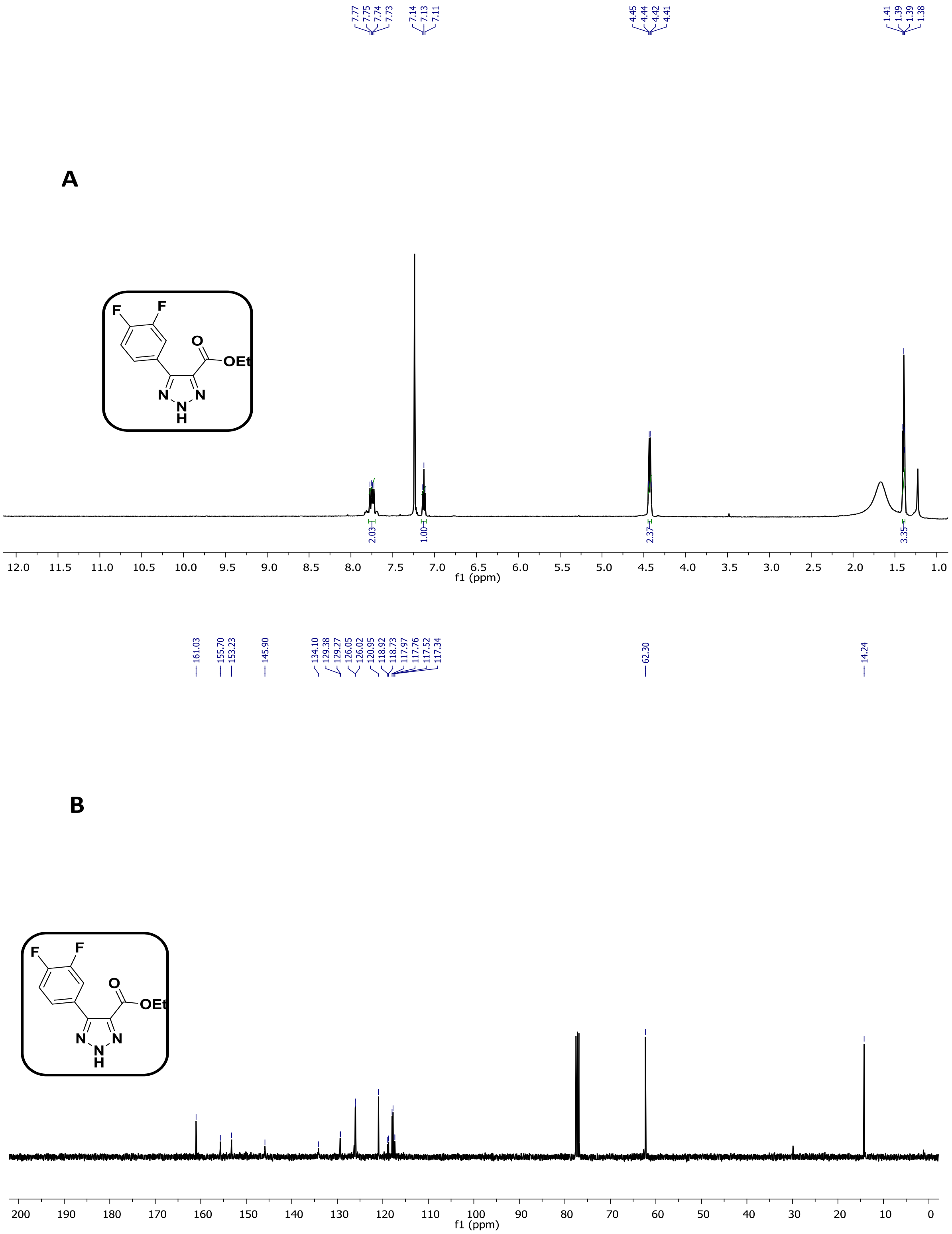

Figure S29. ^{1}H (A) and ^{13}C (B) NMR of compound **2i**.

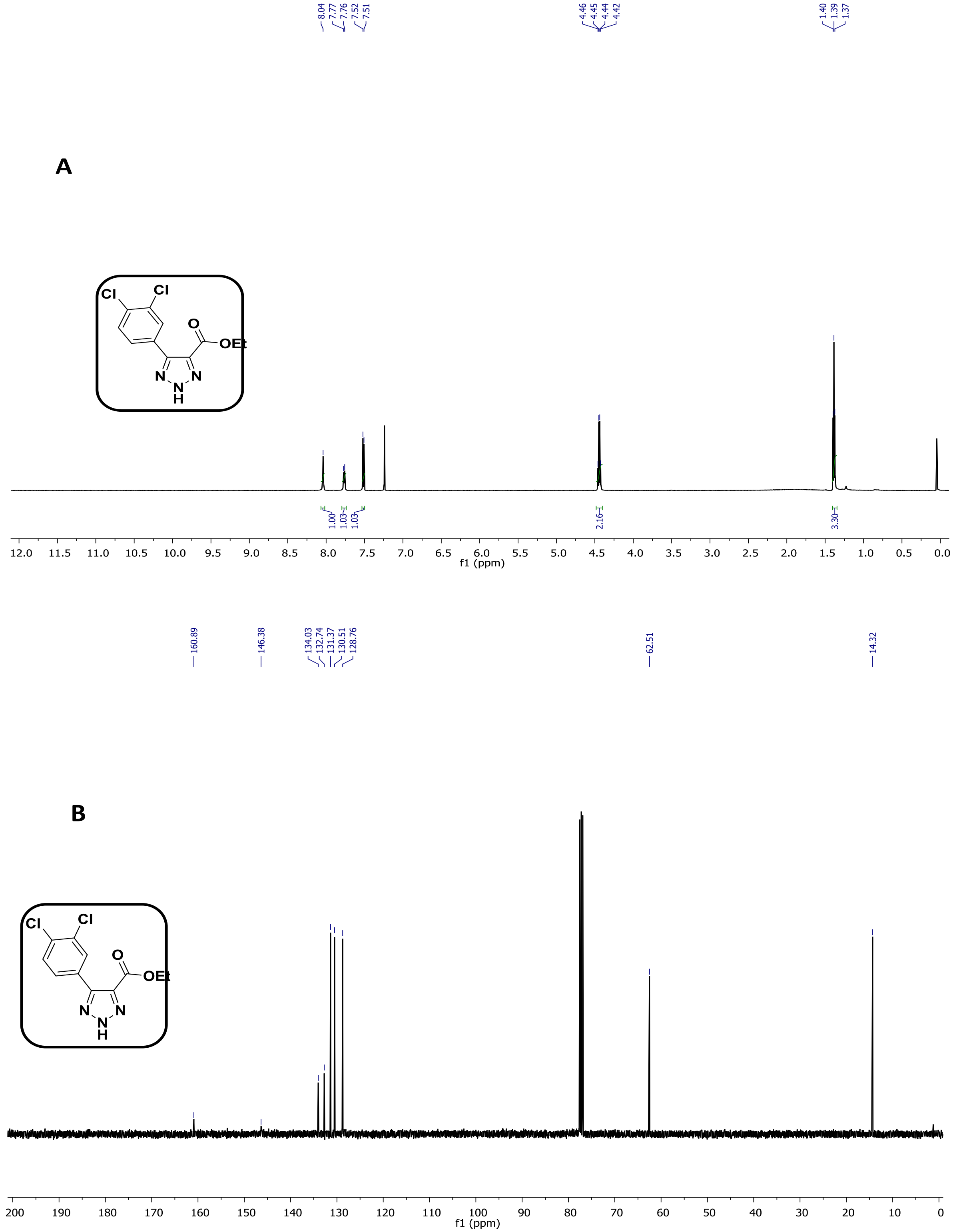

Figure S30. ^{1}H (A) and ^{13}C (B) NMR of compound **2j.**

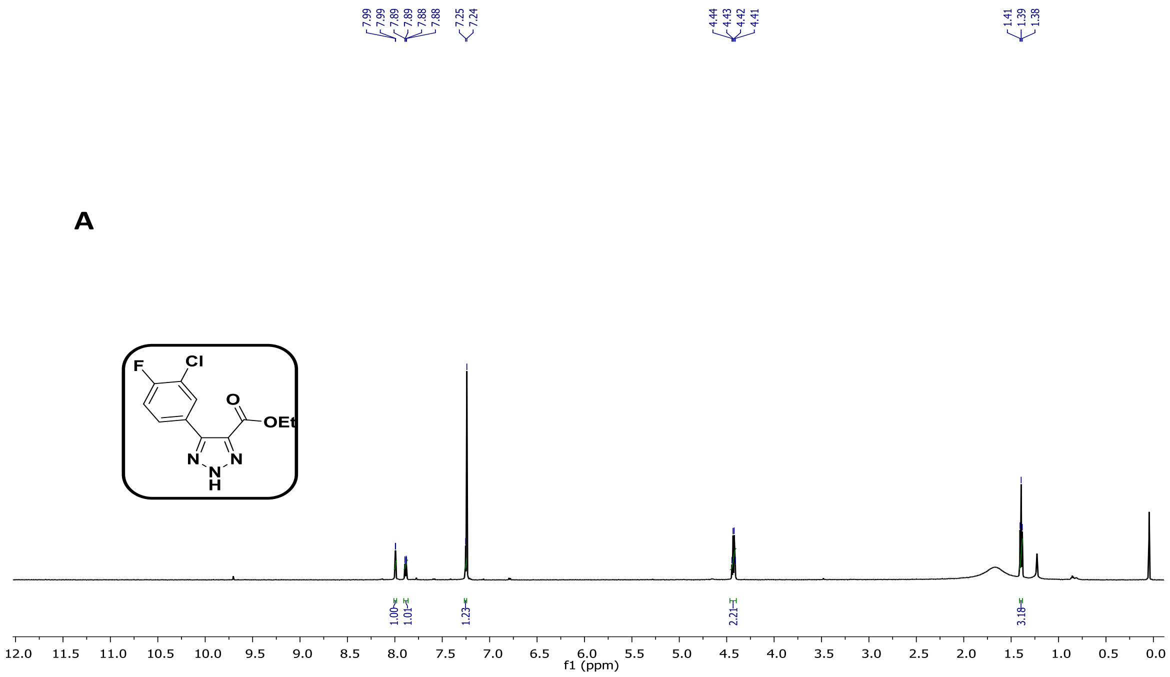

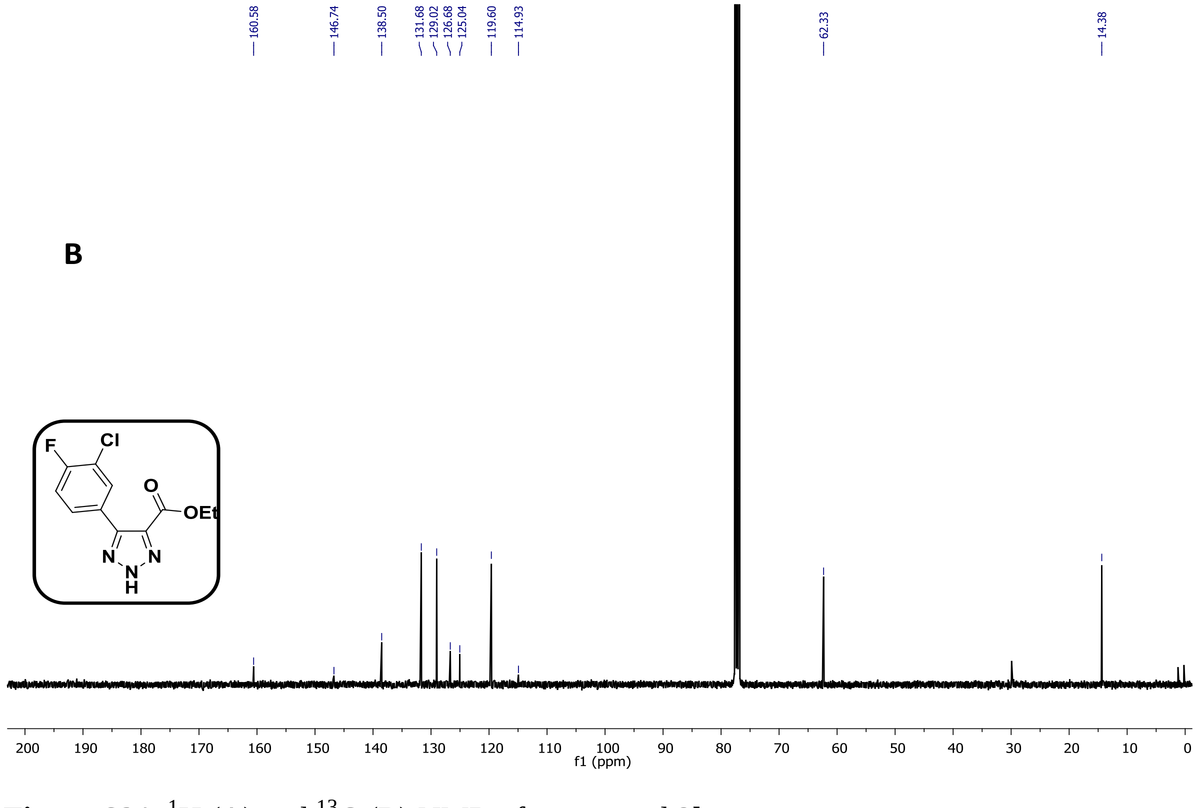

Figure S31. ^{1}H (A) and ^{13}C (B) NMR of compound **2k.**

A

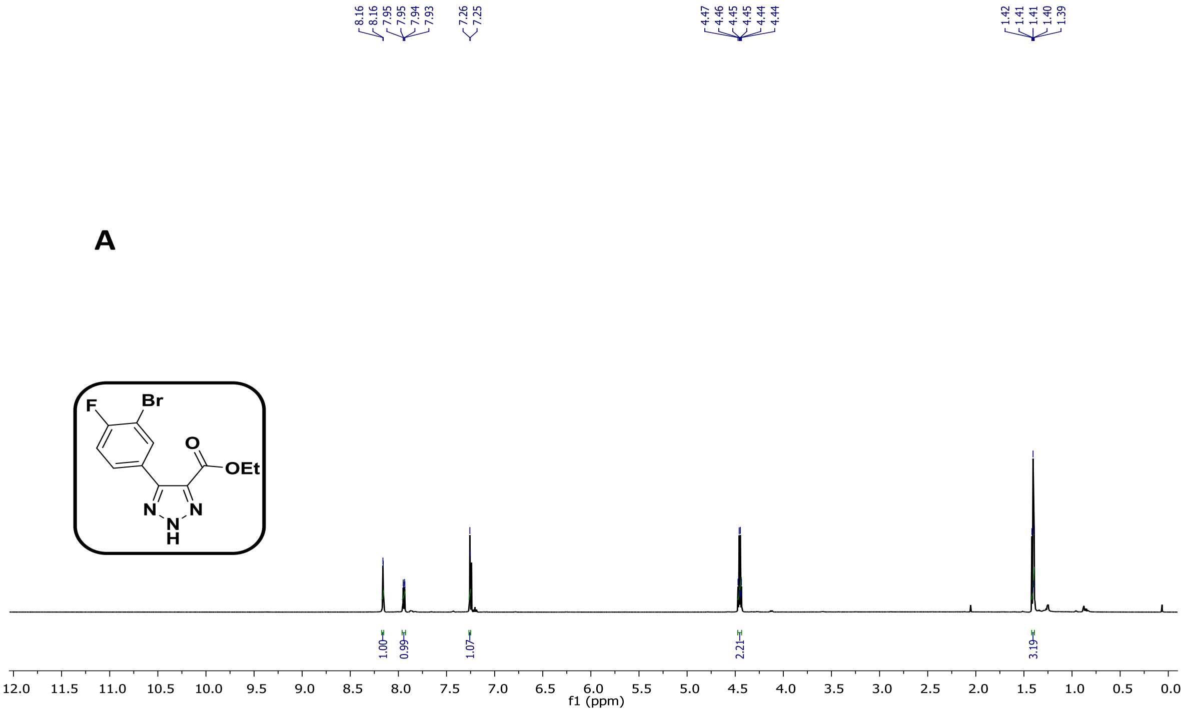

B

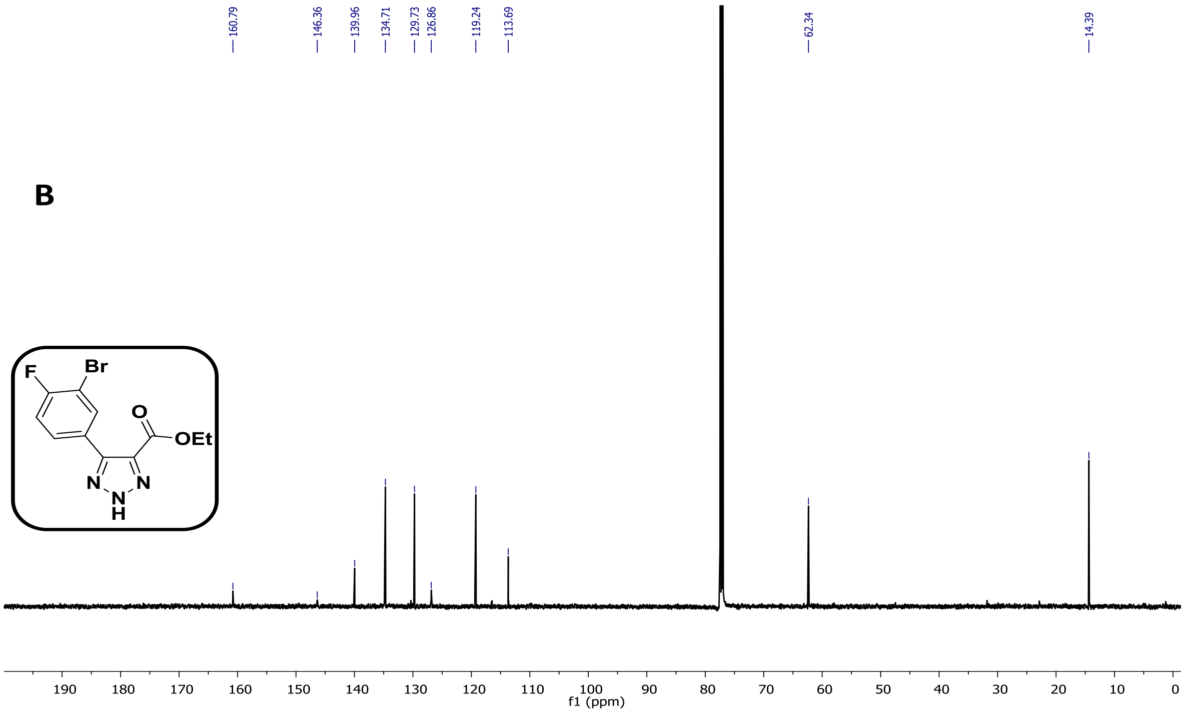

Figure S32. ^{1}H (A) and ^{13}C (B) NMR of compound **2l.**

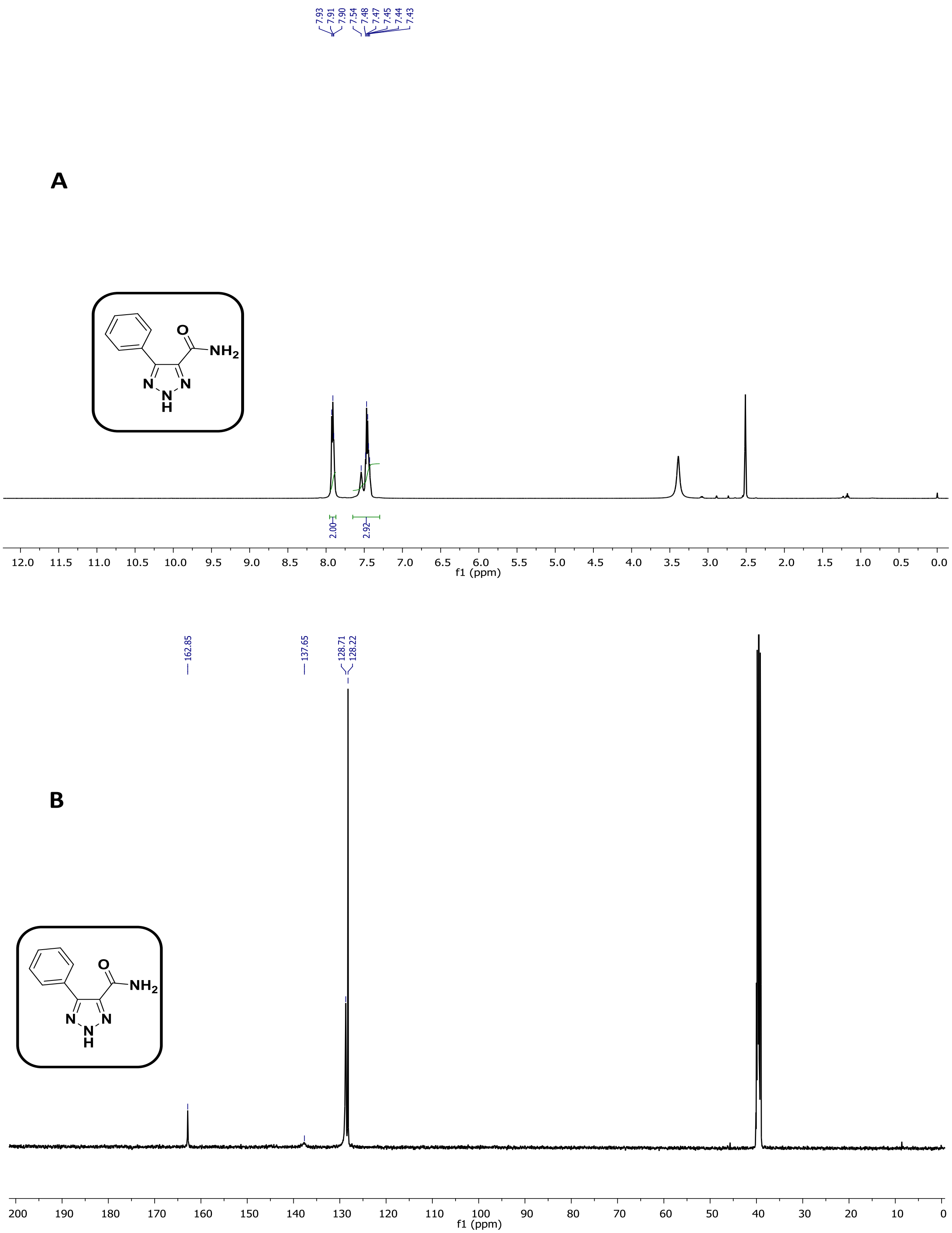

Figure S33. ^{1}H (A) and ^{13}C (B) NMR of compound **3a.**

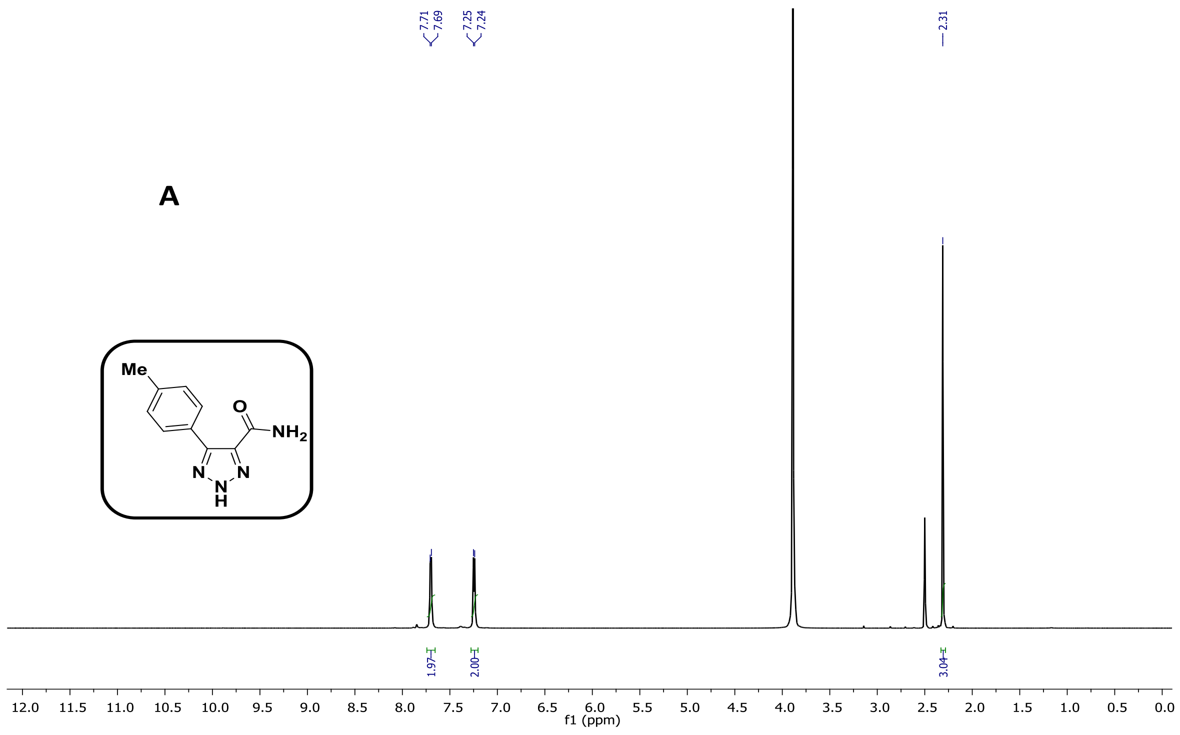

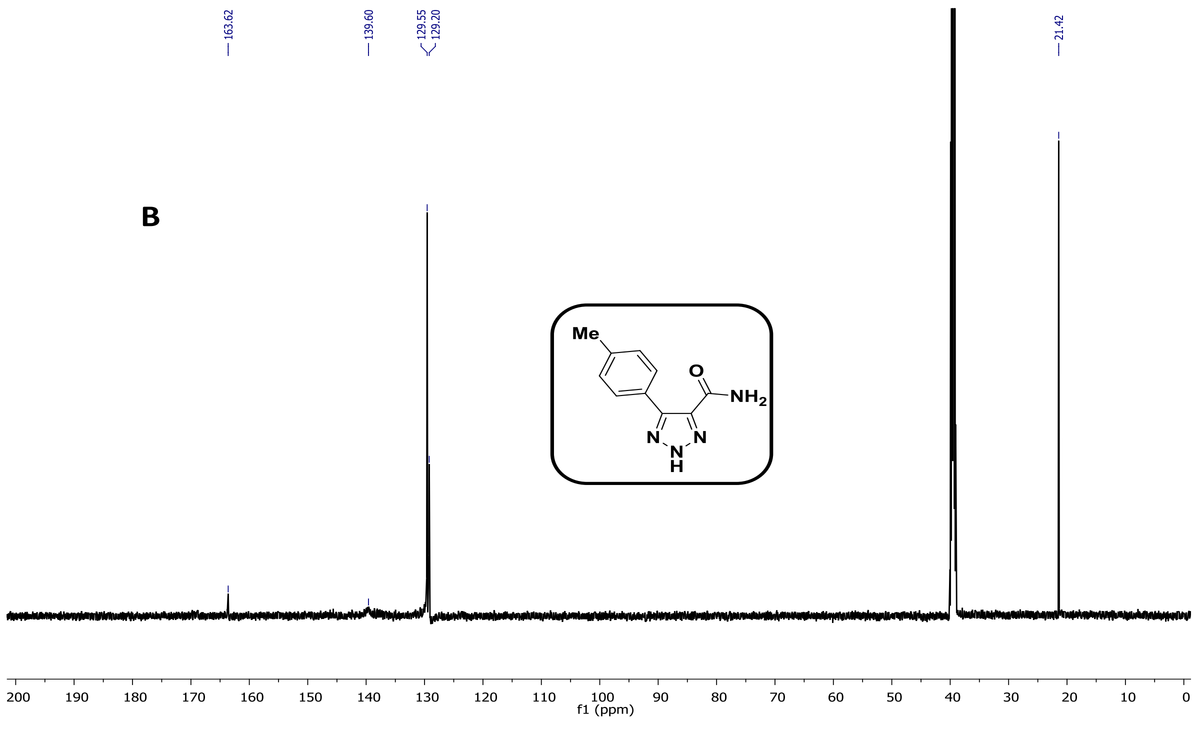

Figure S34. ^{1}H (A) and ^{13}C (B) NMR of compound **3b.**

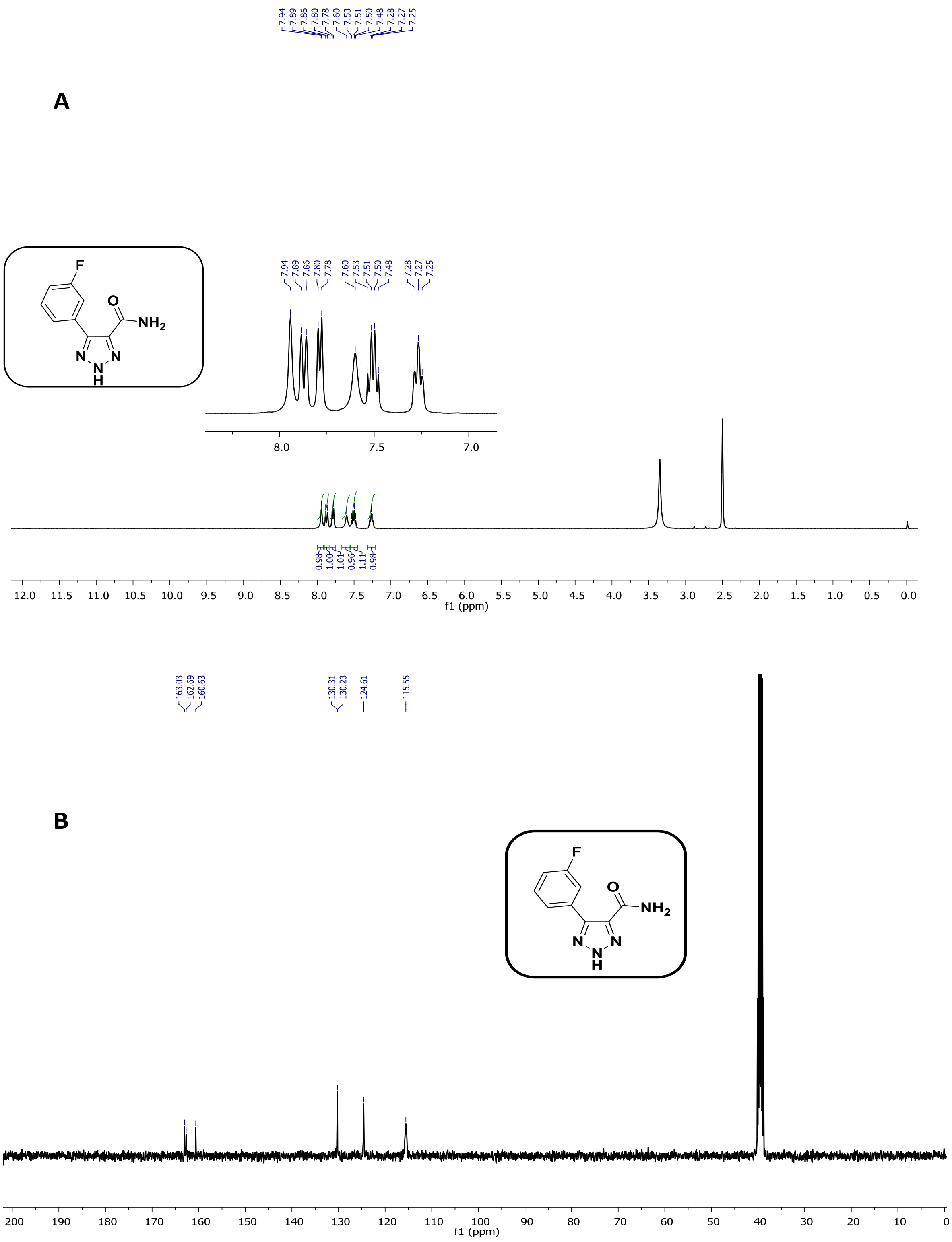

Figure S35. ^{1}H (A) and ^{13}C (B) NMR of compound **3c.**

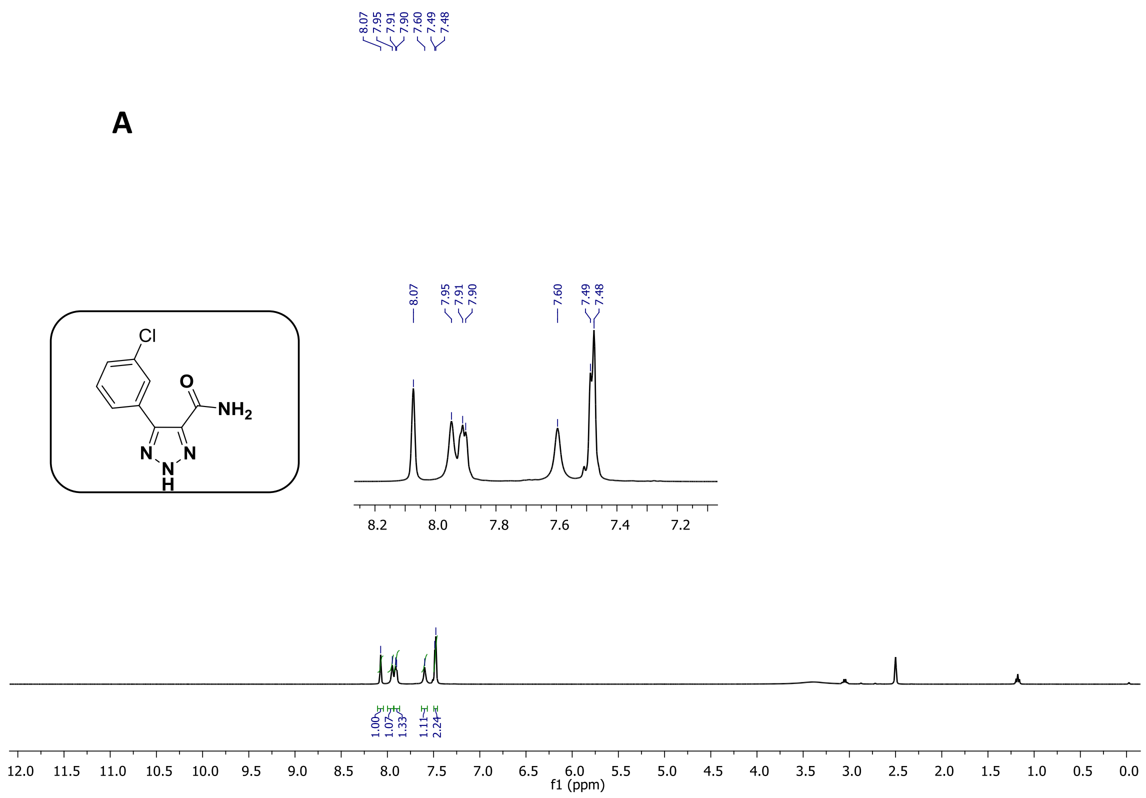

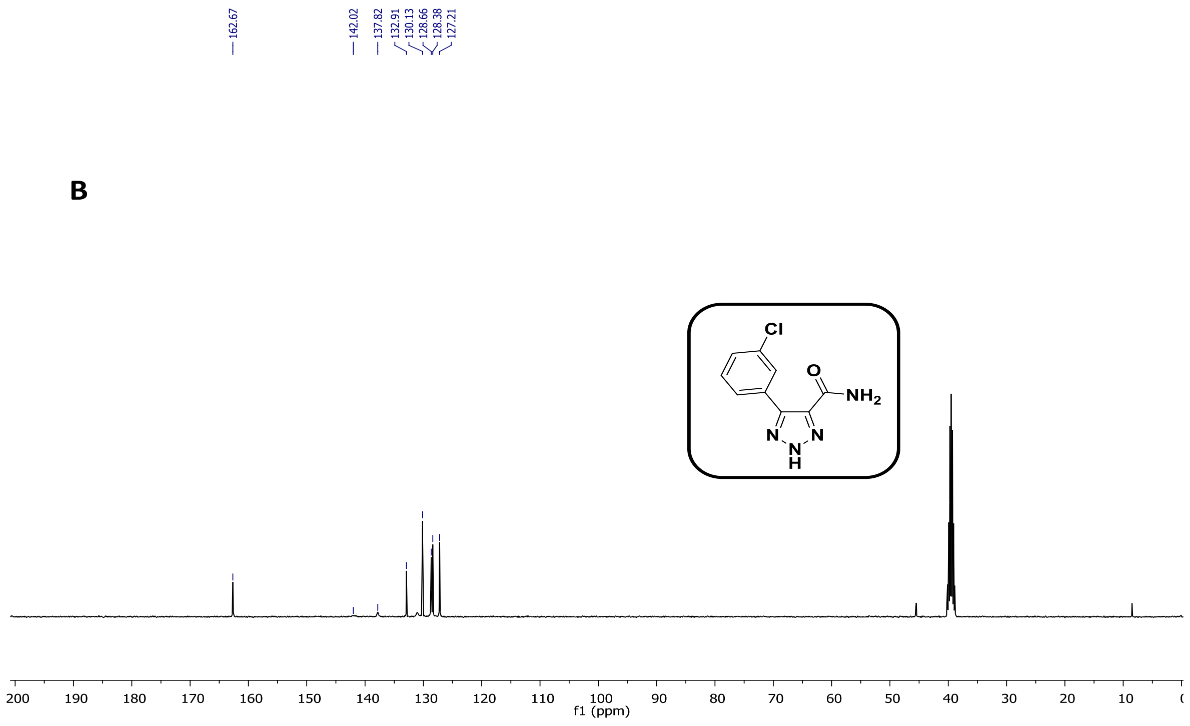

Figure S36. ^{1}H (A) and ^{13}C (B) NMR of compound **3d.**

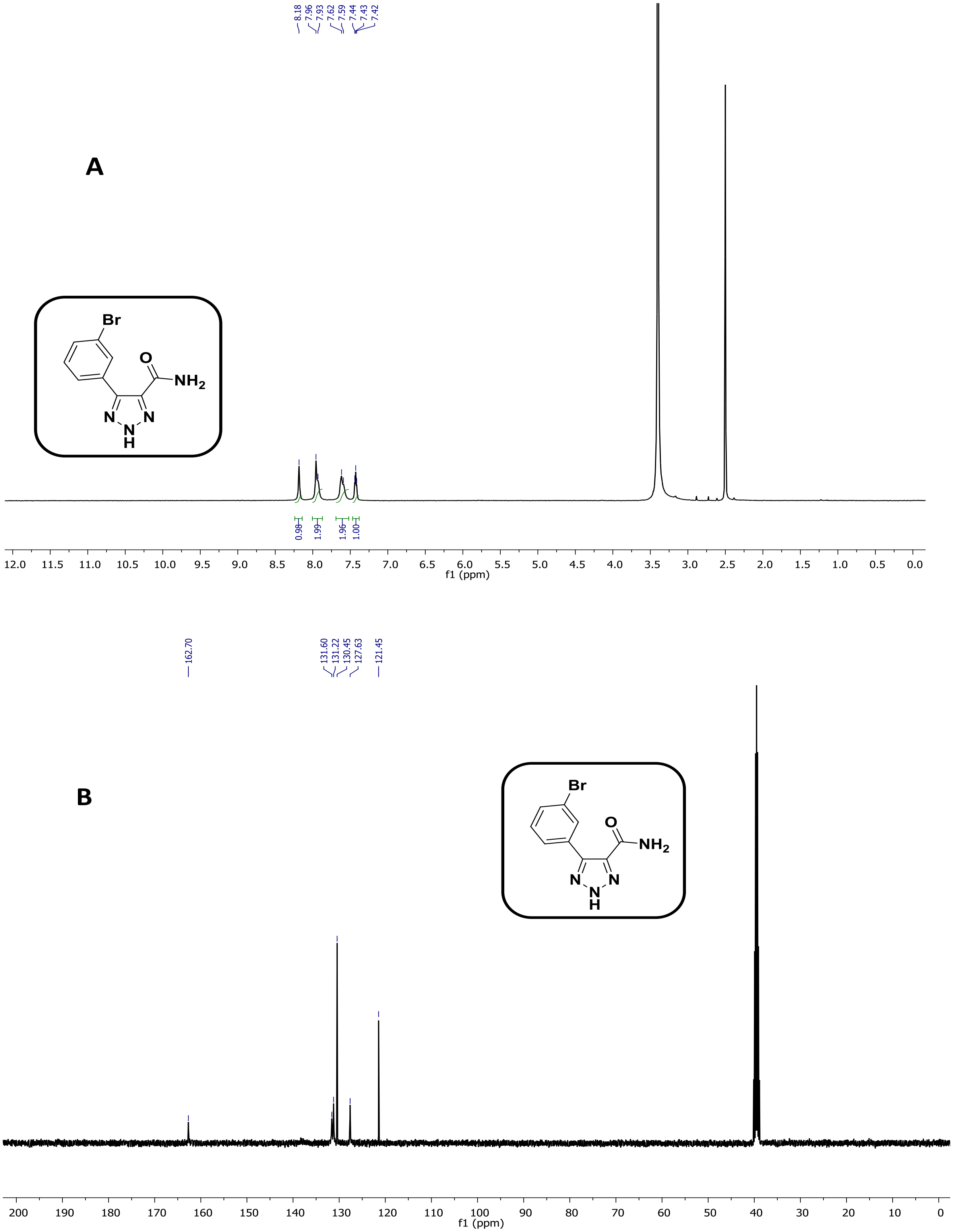

Figure S37. ^{1}H (A) and ^{13}C (B) NMR of compound **3e.**

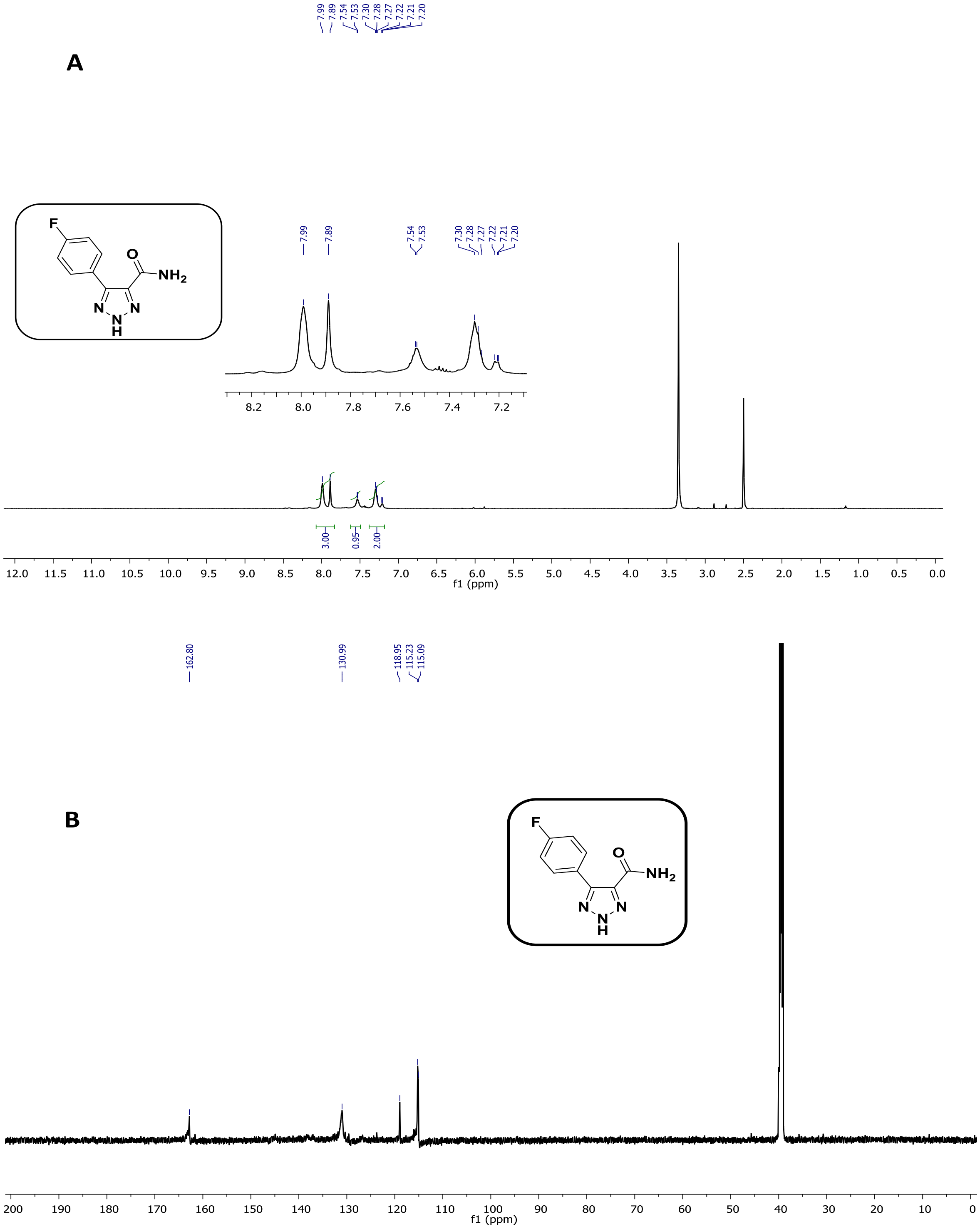

Figure S38. ^{1}H (A) and ^{13}C (B) NMR of compound **3f.**

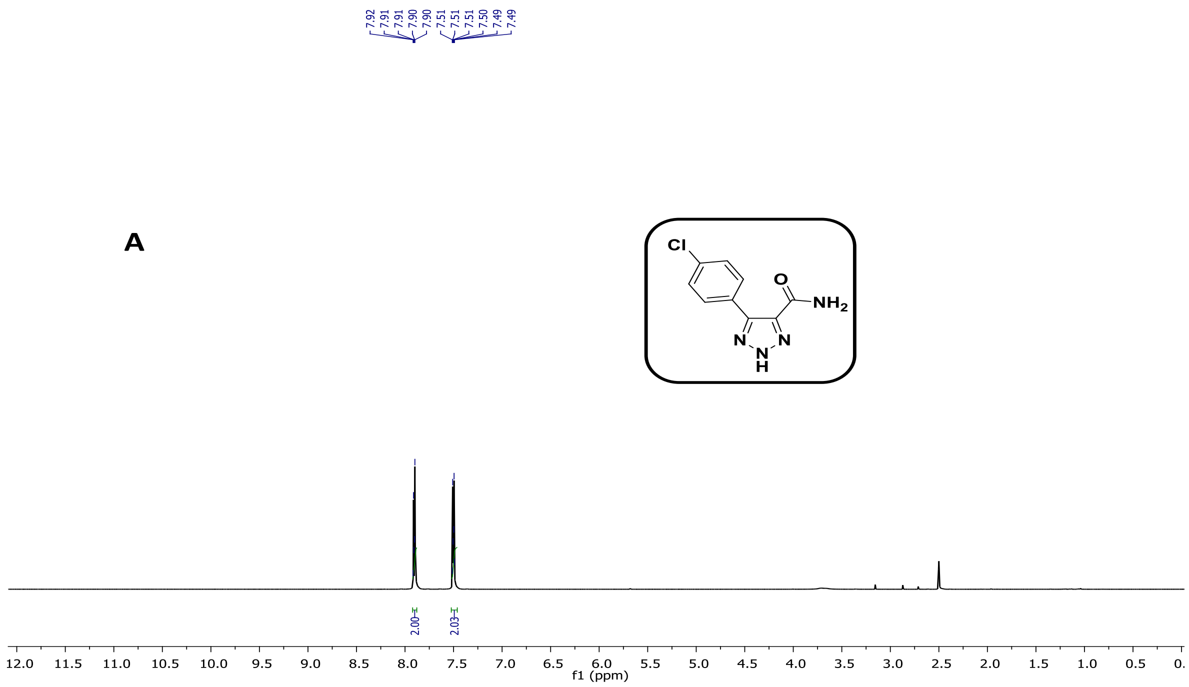

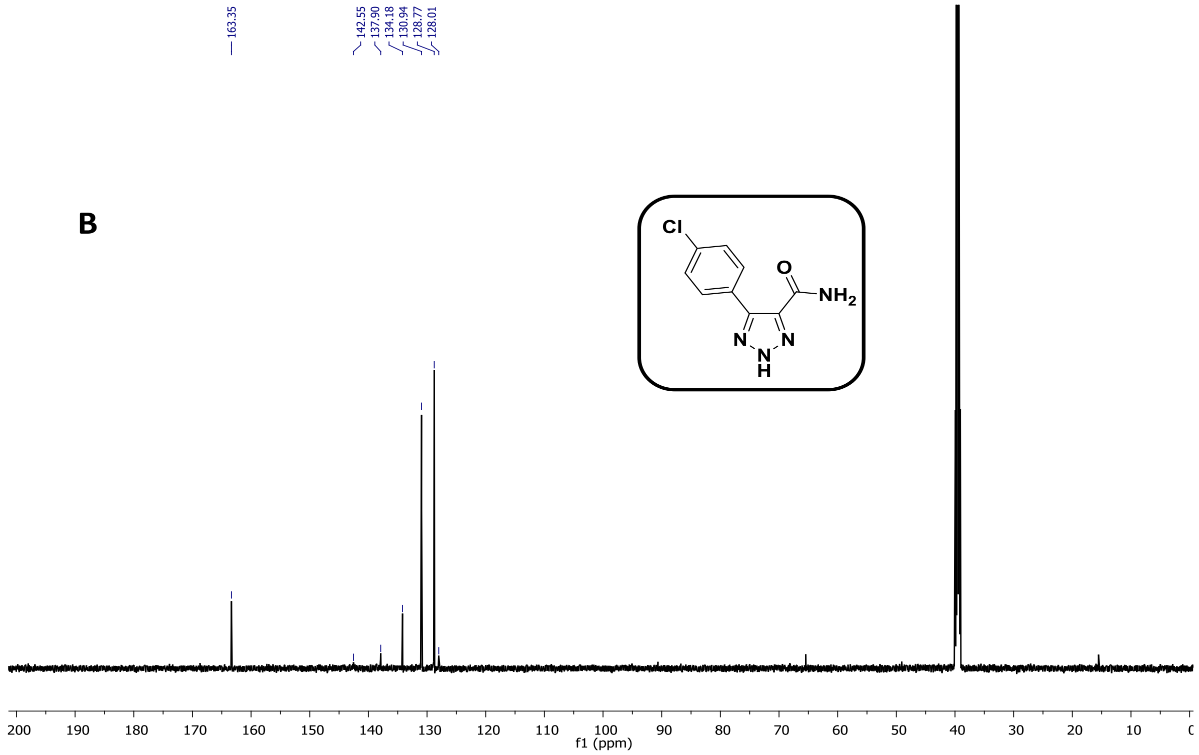

Figure S39. ^{1}H (A) and ^{13}C (B) NMR of compound **3g.**

A

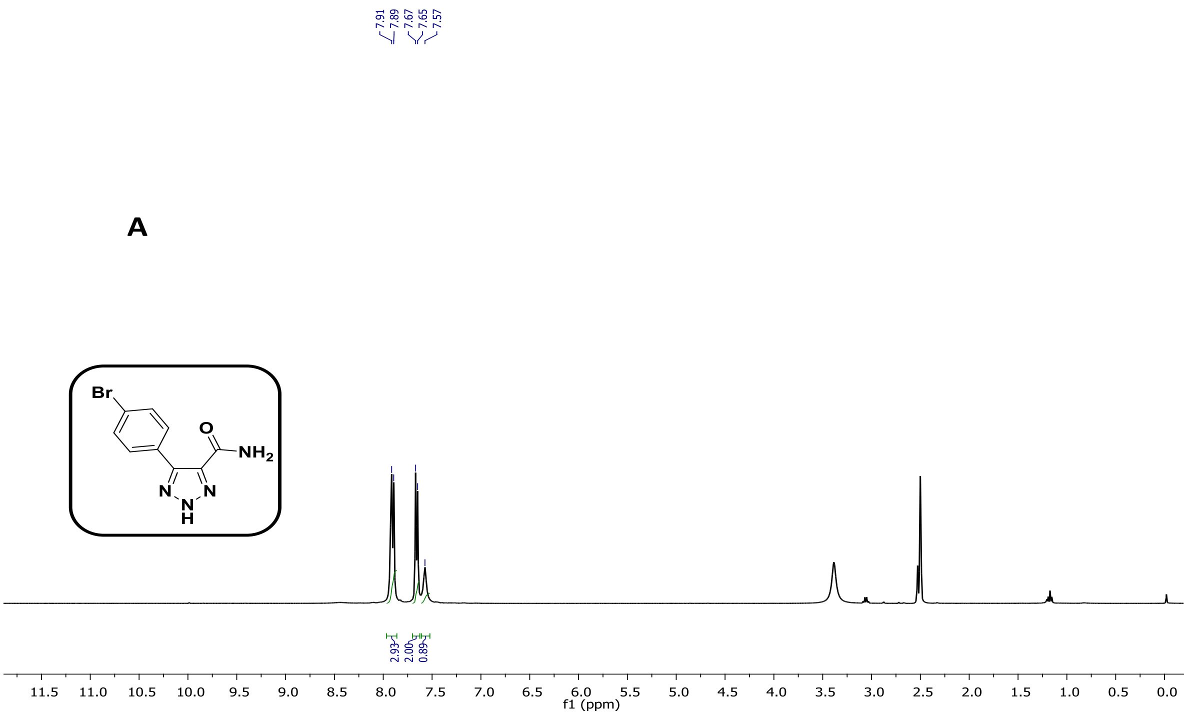

B

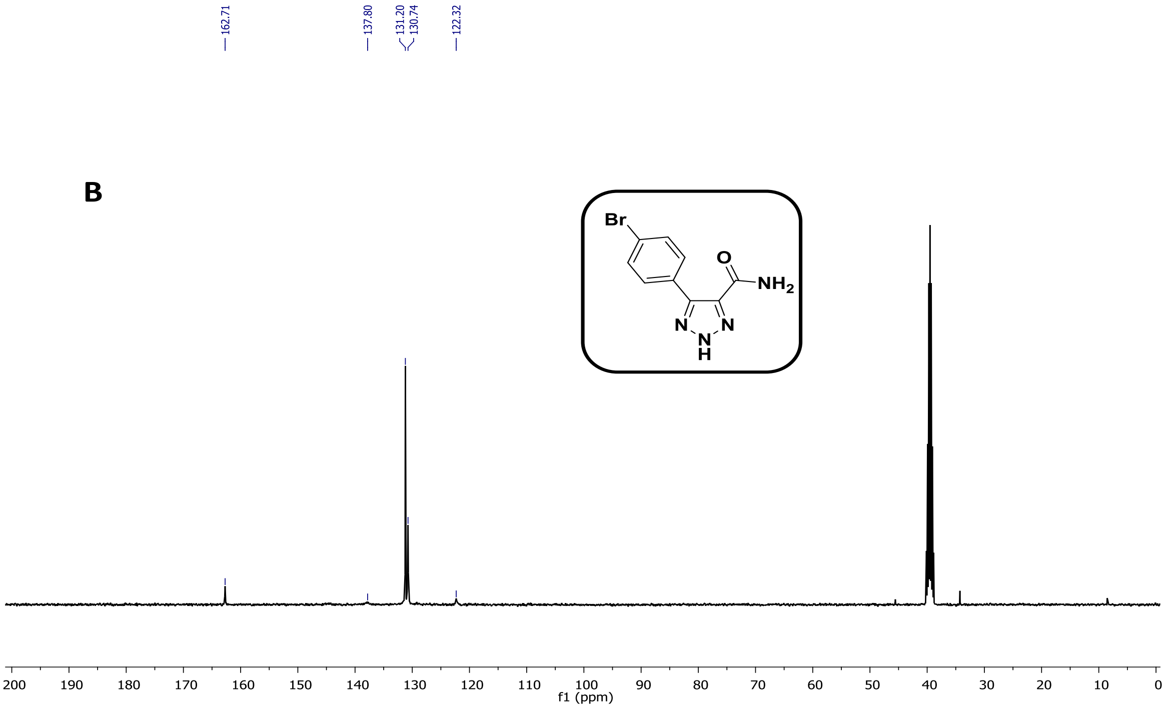

Figure S40. ^{1}H (A) and ^{13}C (B) NMR of compound **3h.**

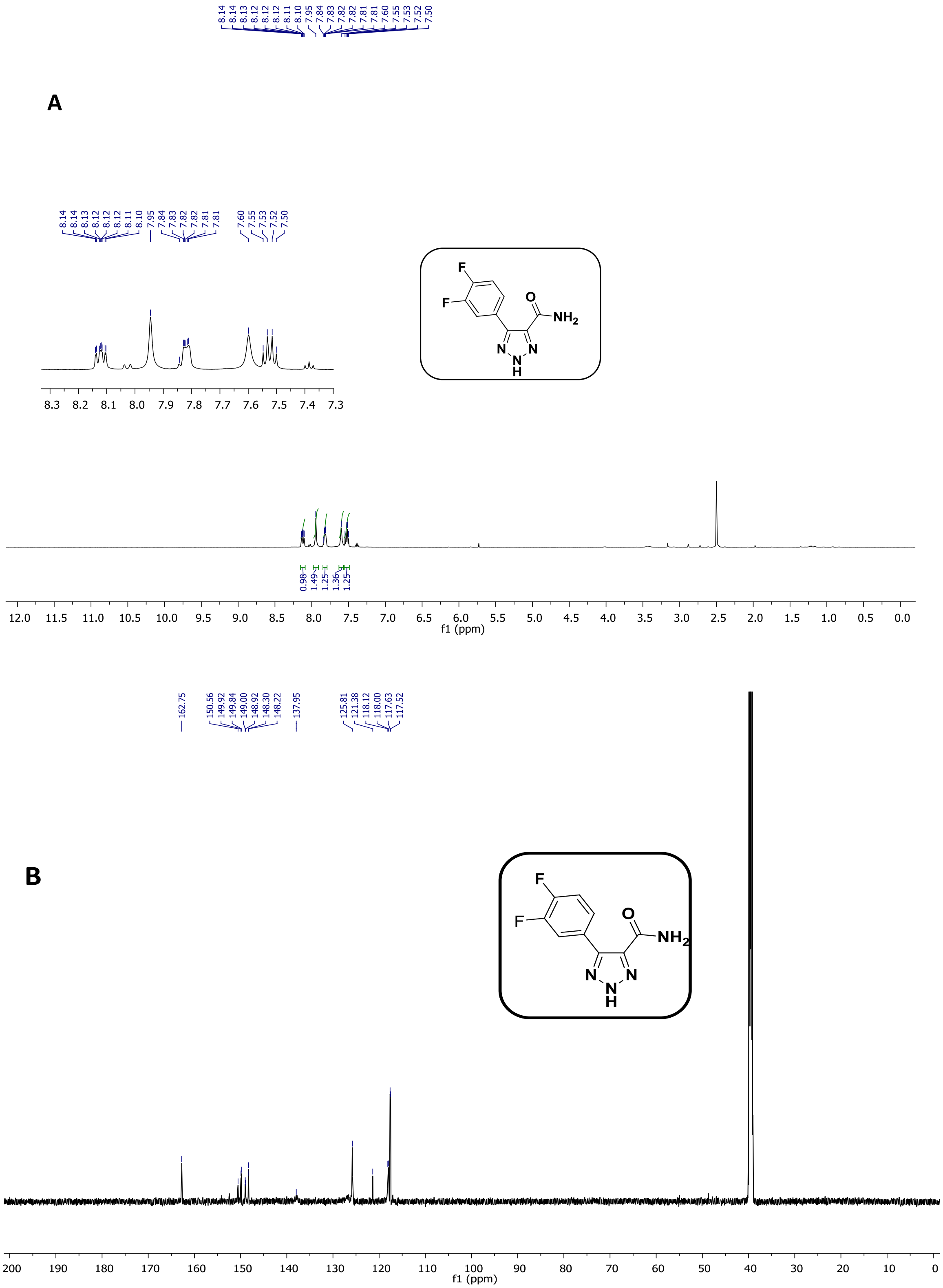

Figure S41. ^{1}H (A) and ^{13}C (B) NMR of compound **3i.**

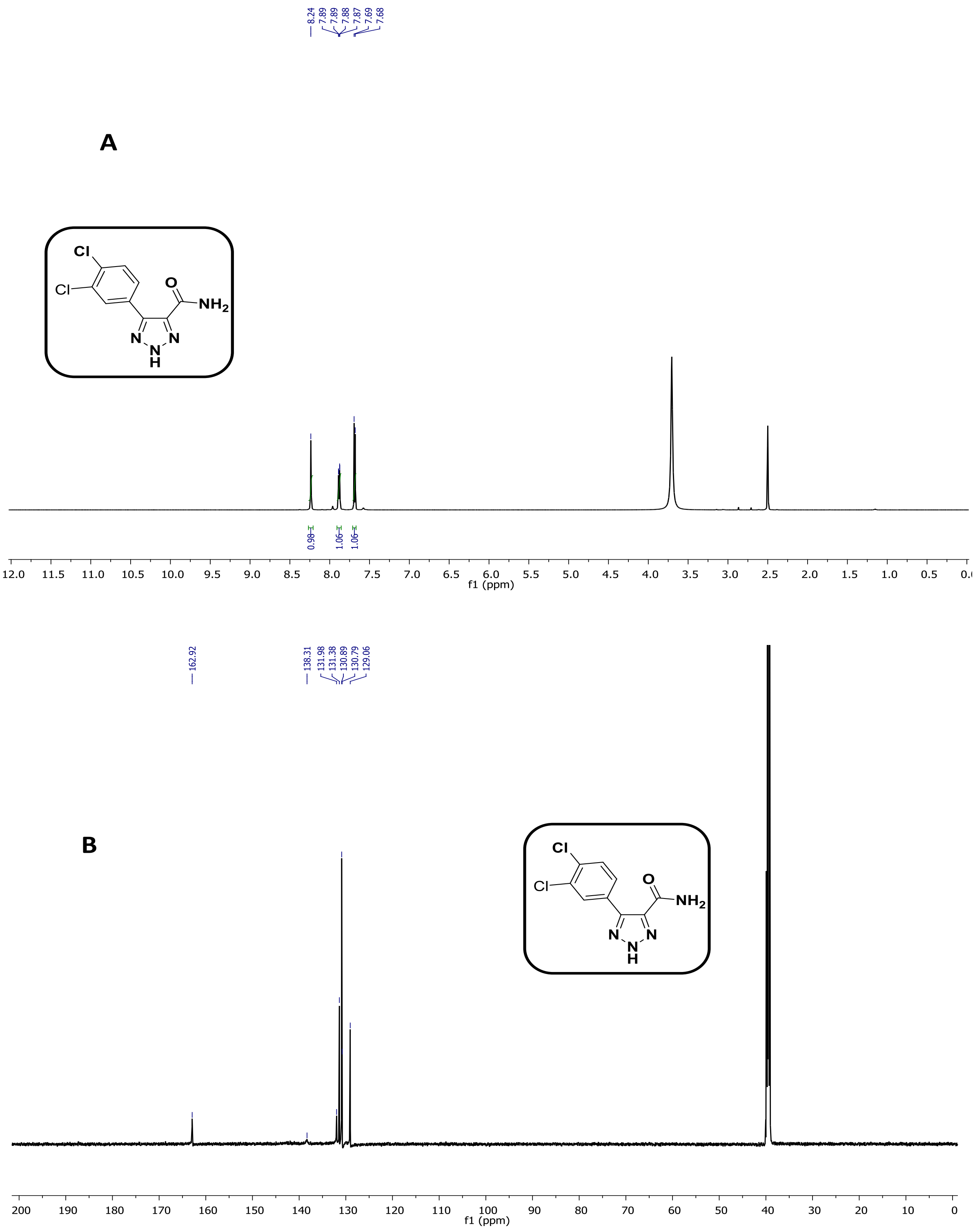

Figure S42. ^{1}H (A) and ^{13}C (B) NMR of compound **3j.**

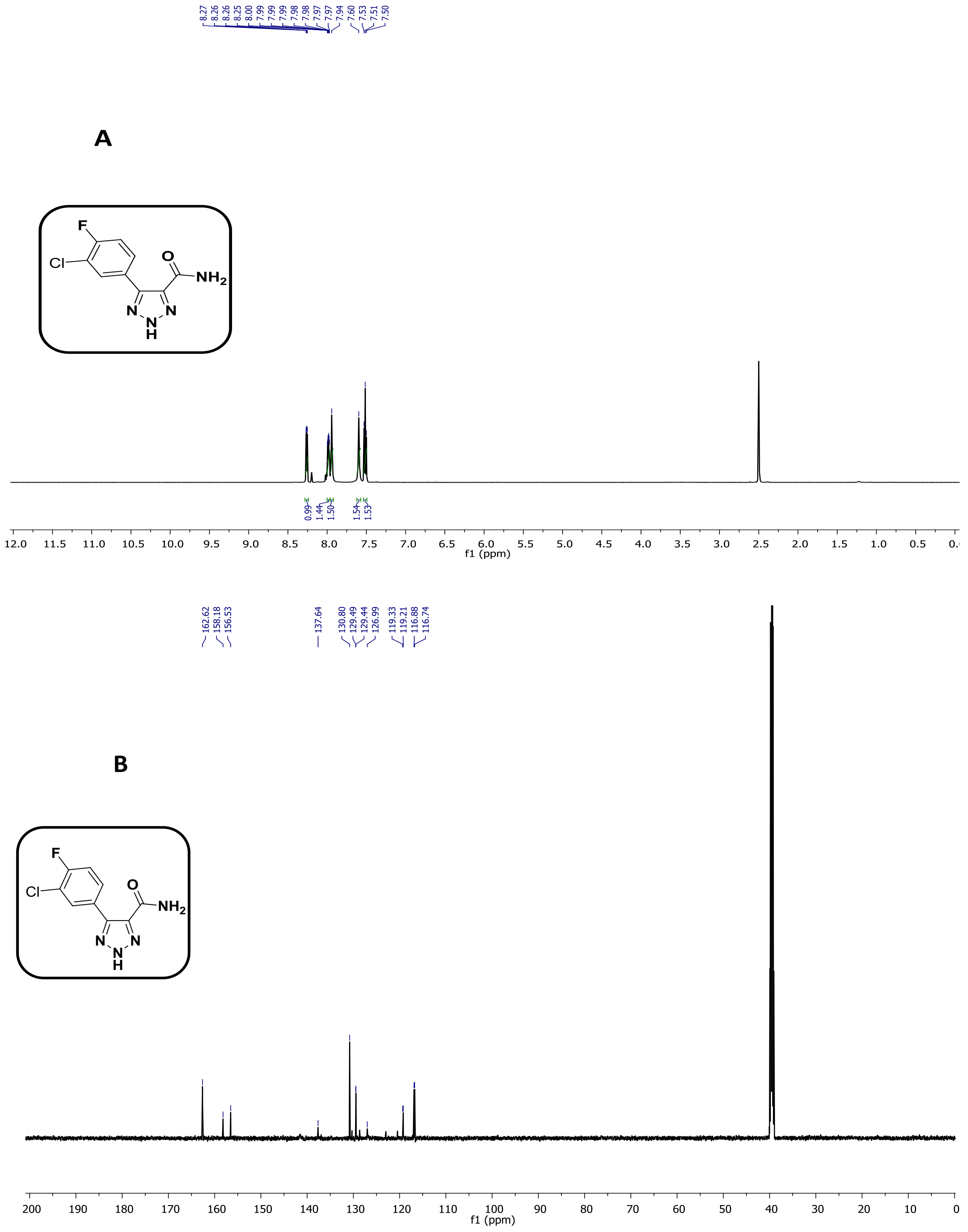

Figure S43. ^{1}H (A) and ^{13}C (B) NMR of compound **3k.**

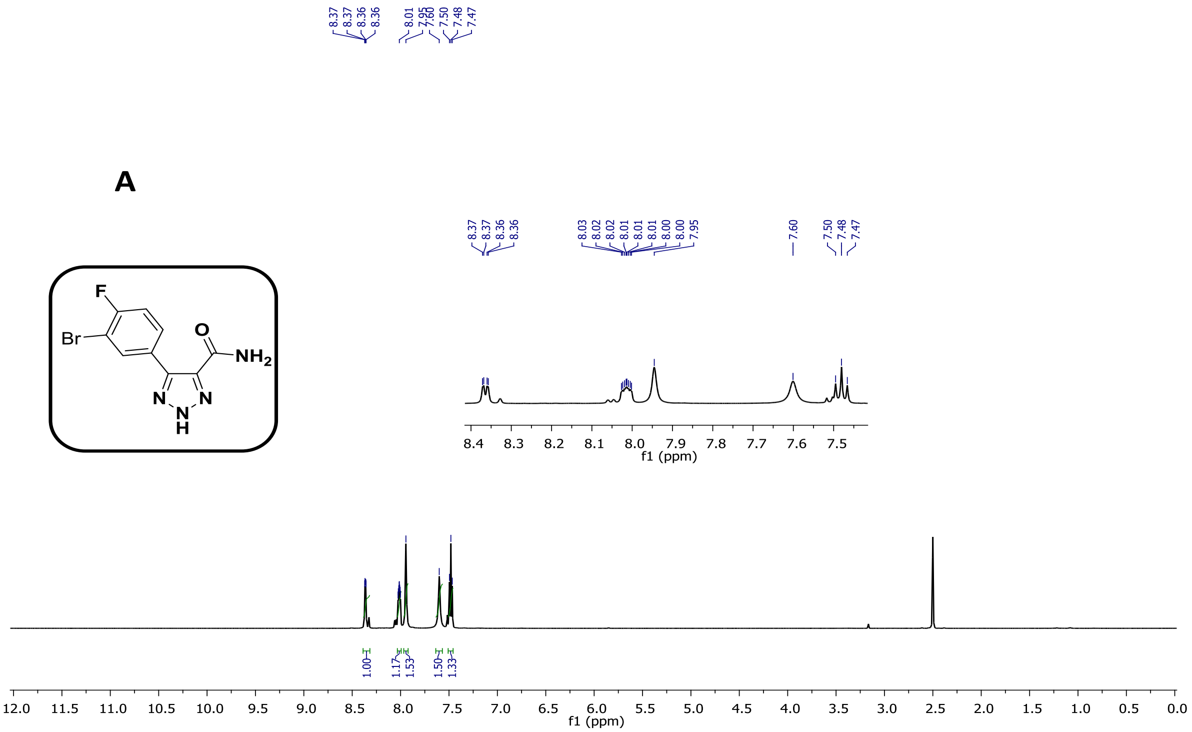

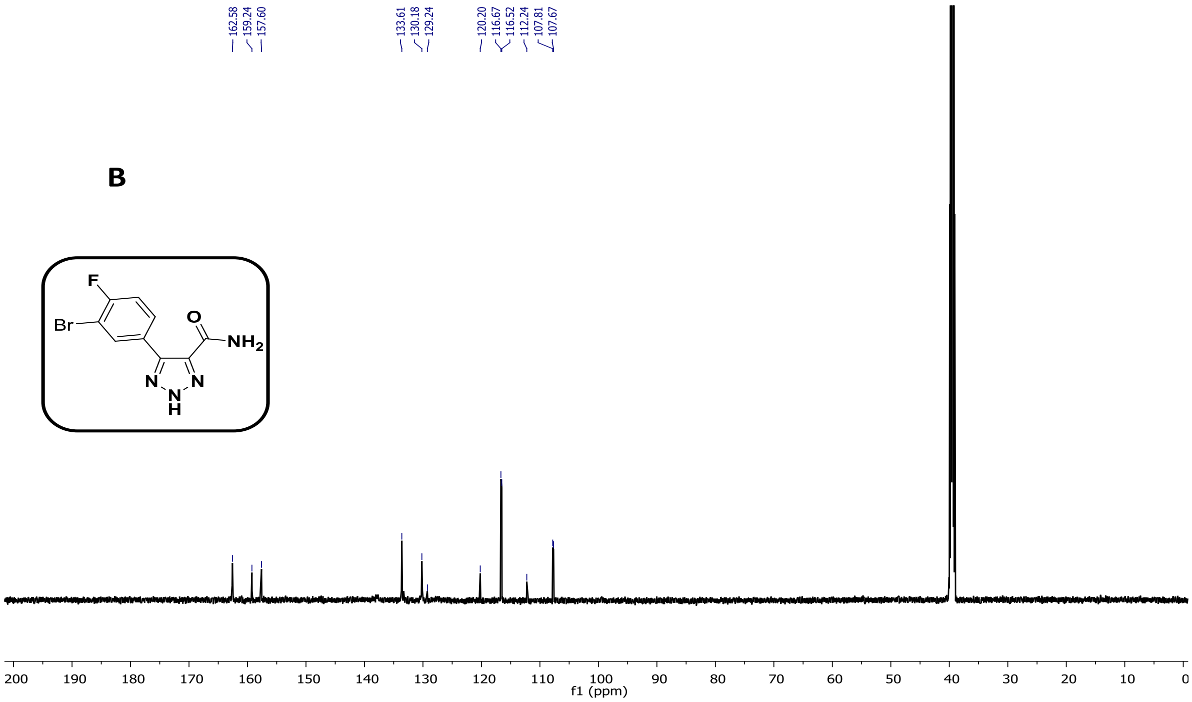

Figure S44. ^{1}H (A) and ^{13}C (B) NMR of compound **3l.**

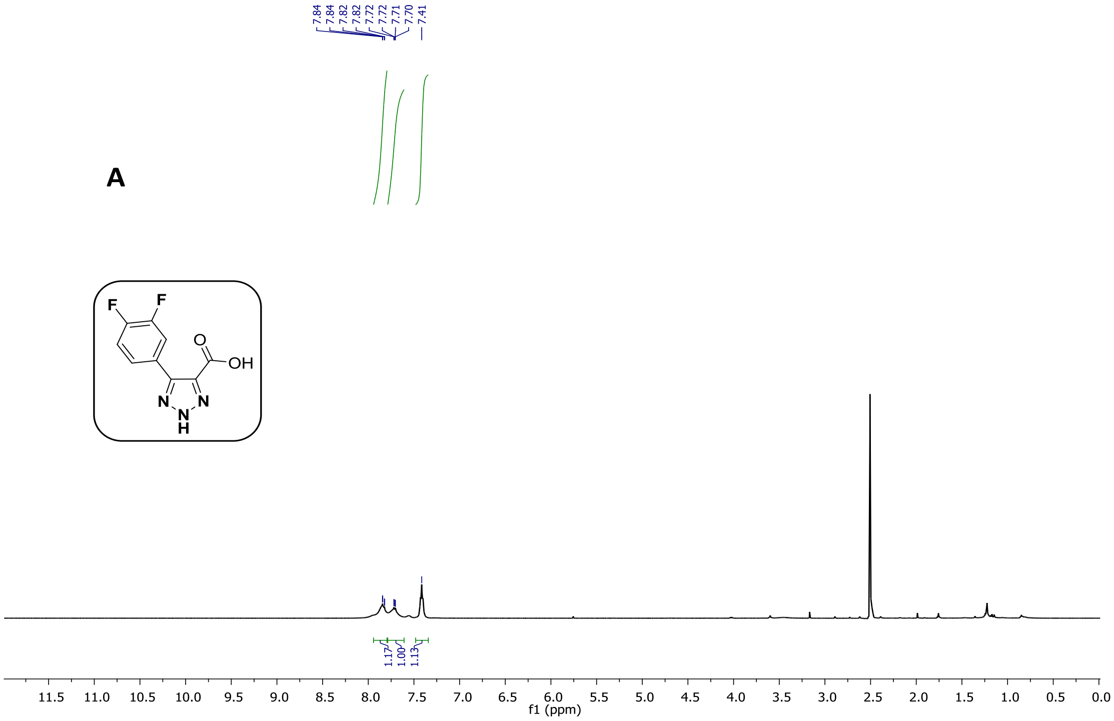

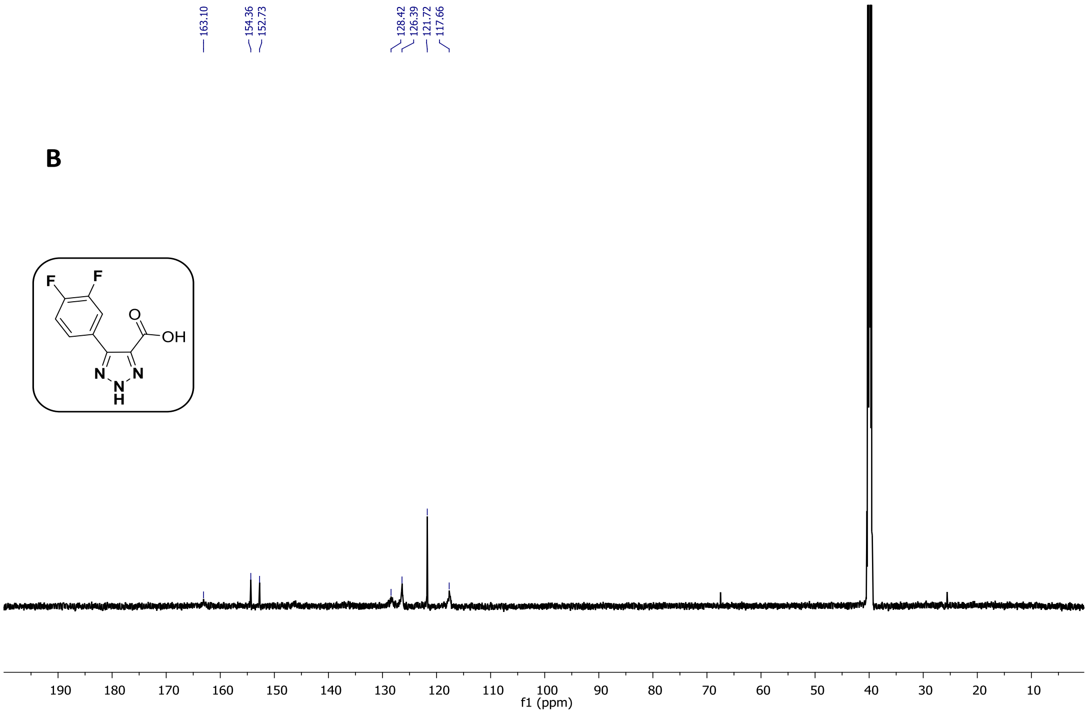

Figure S45. ^{1}H (A) and ^{13}C (B) NMR of compound **4a.**

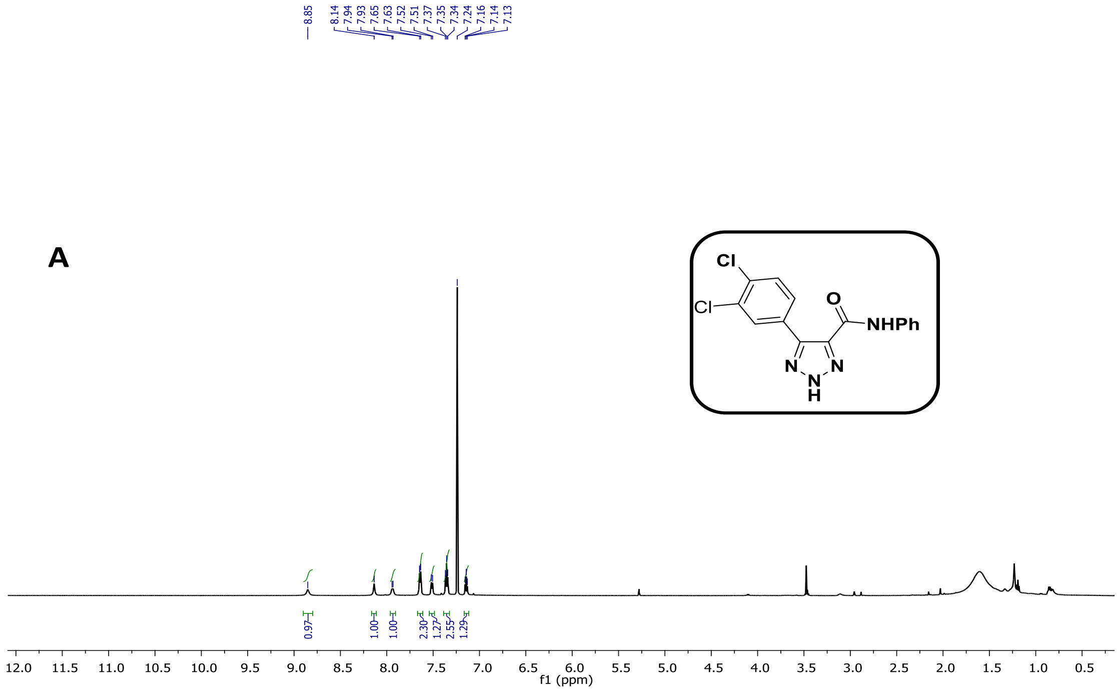

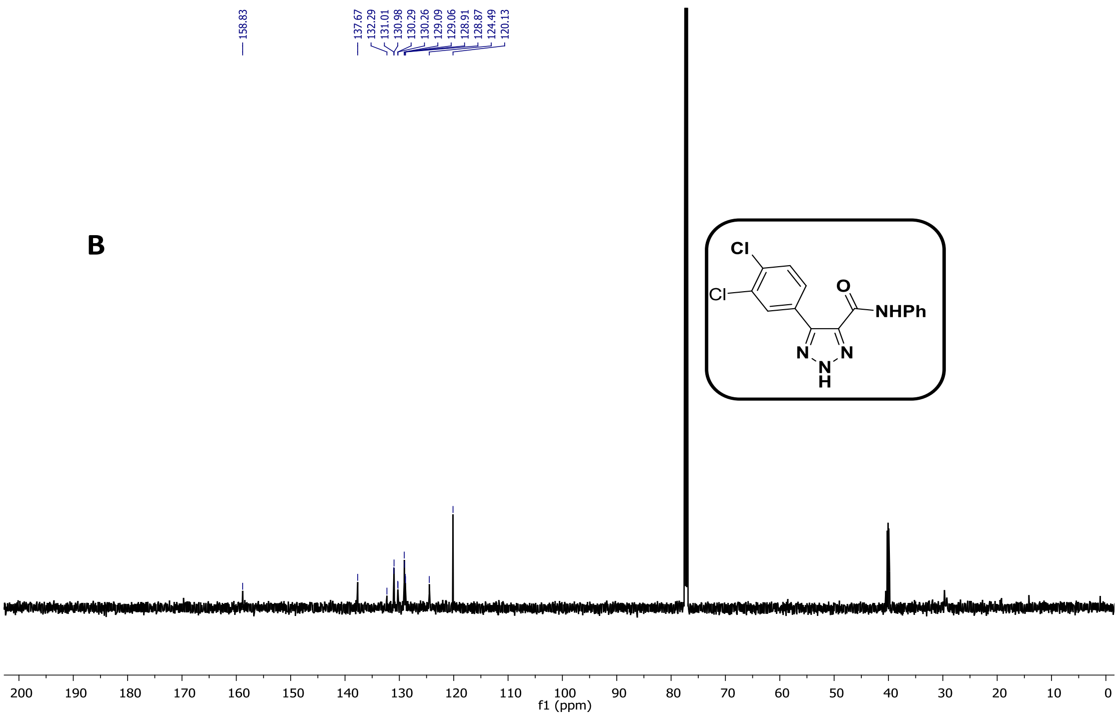

Figure S46. ¹H (A) and ¹³C (B) NMR of compound **4b.**

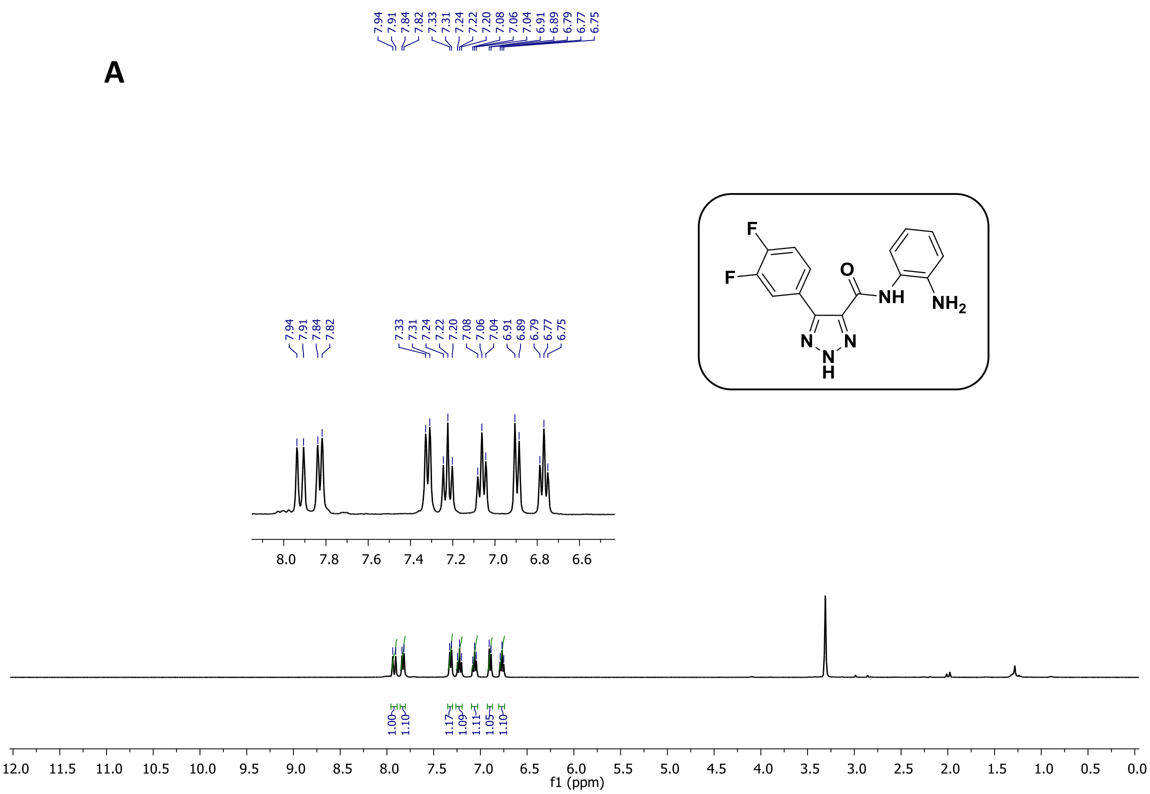

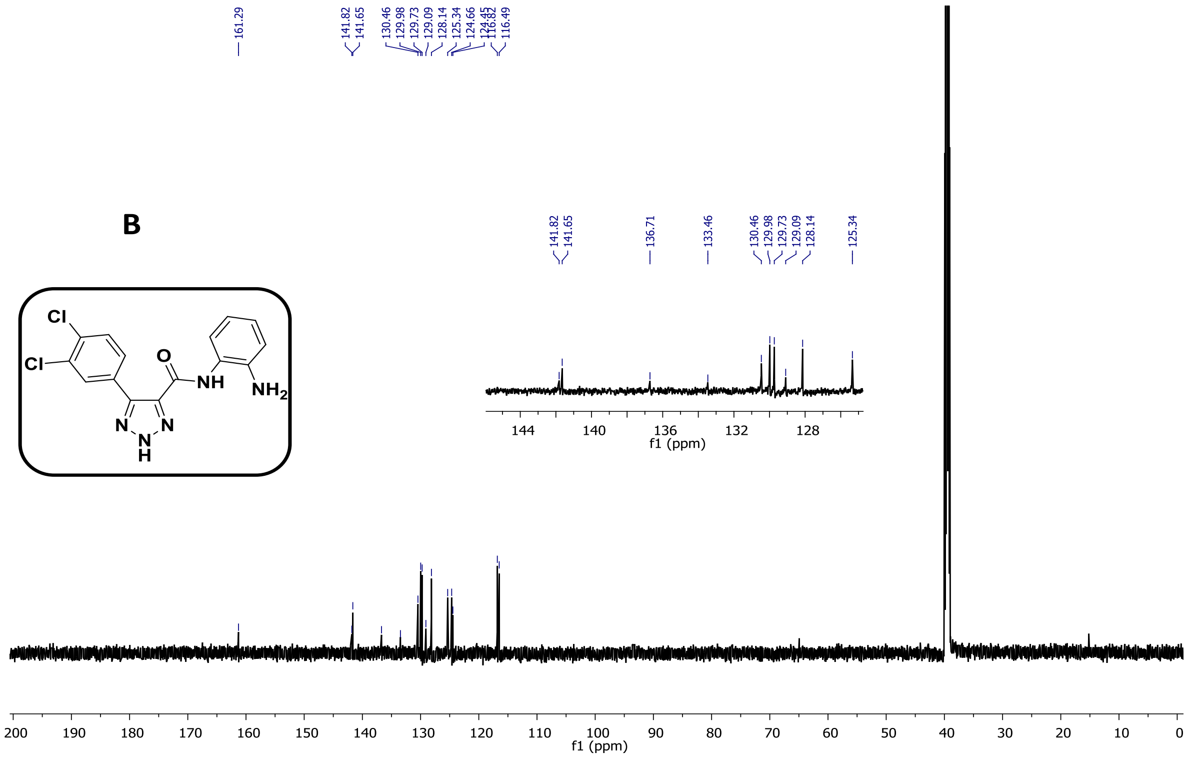

Figure S47. ^{1}H (A) and ^{13}C (B) NMR of compound **4c.**

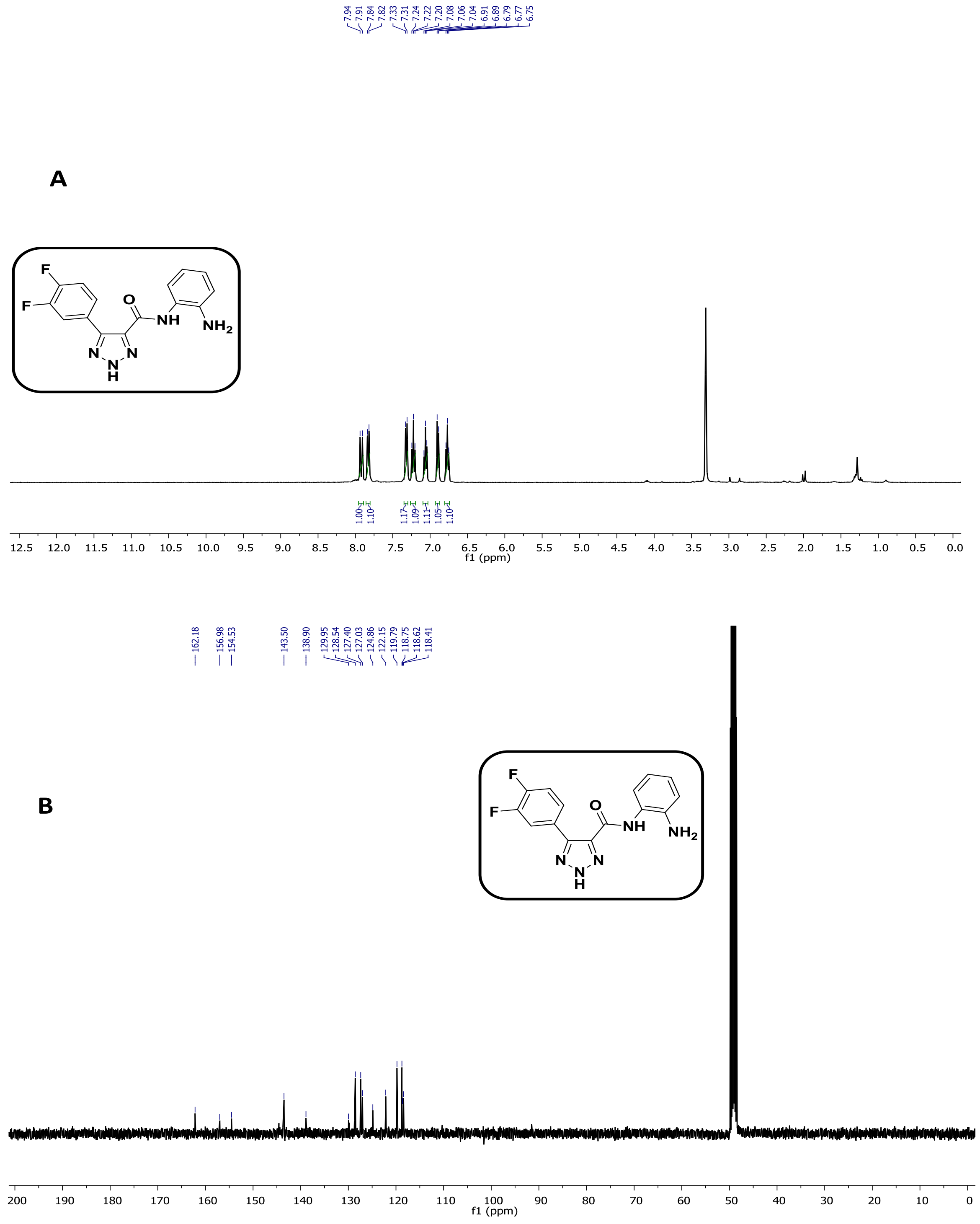

Figure S48. ^{1}H (A) and ^{13}C (B) NMR of compound **4d.**

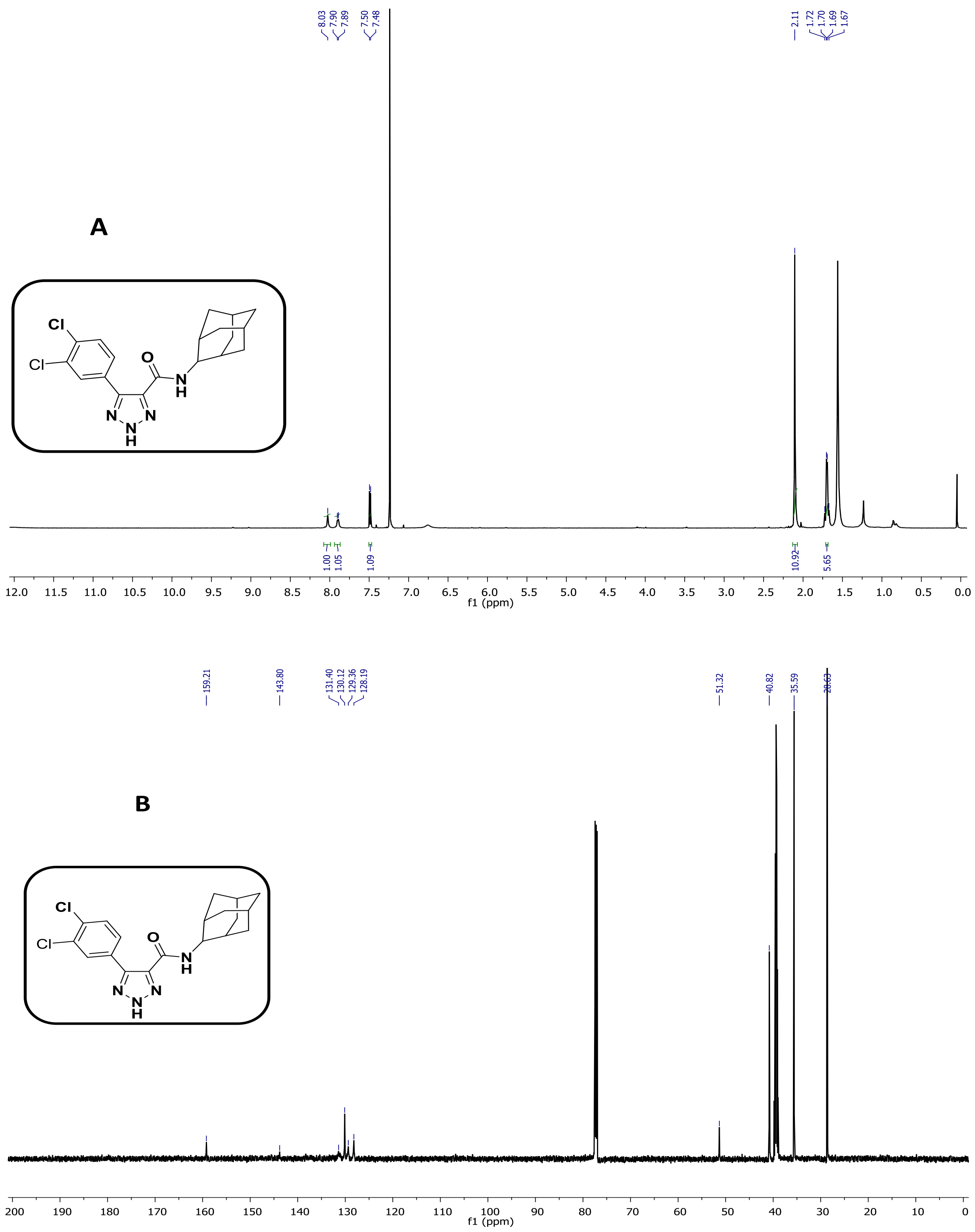

Figure S49. ^{1}H (A) and ^{13}C (B) NMR of compound **4e.**

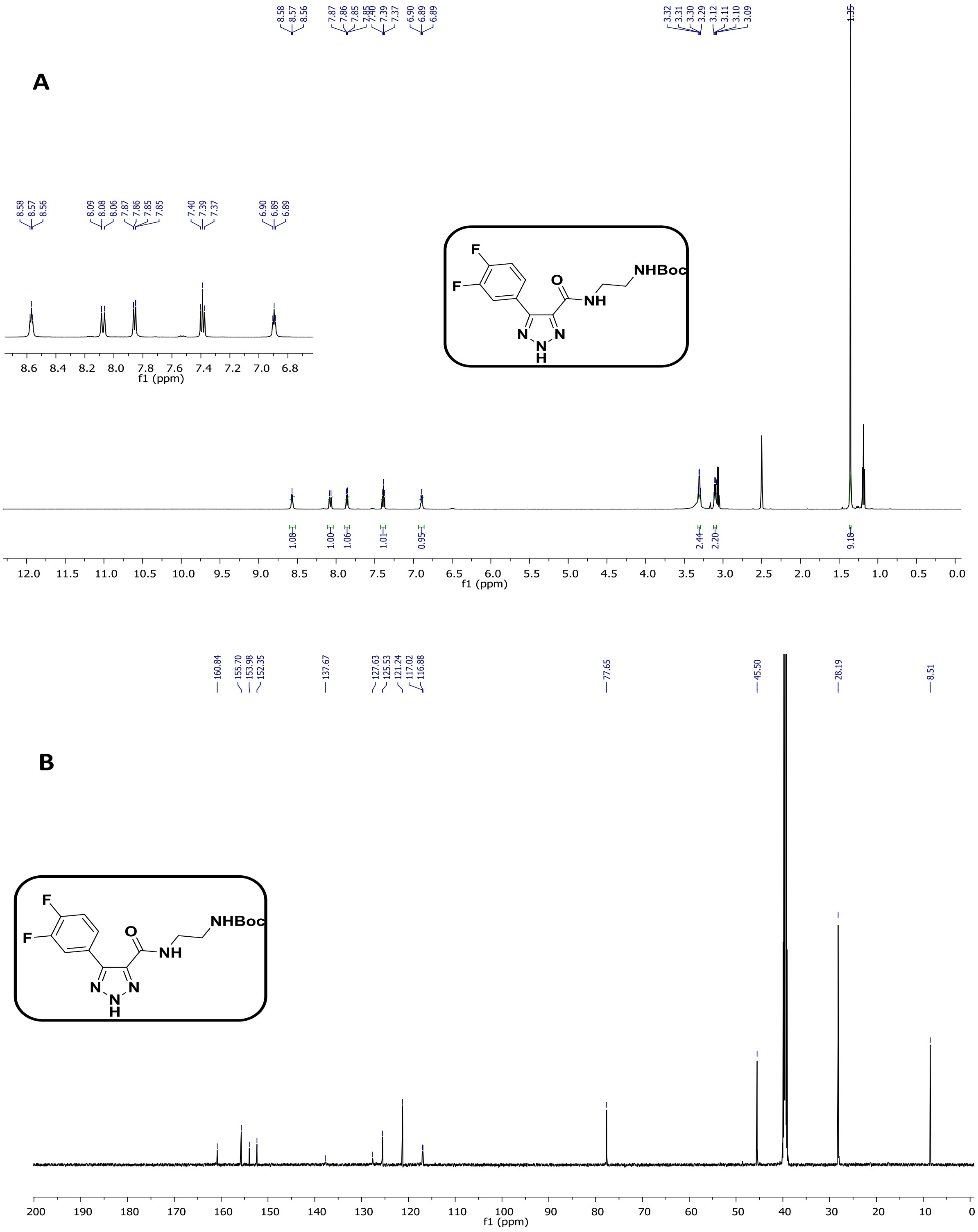

Figure S50. ^{1}H (A) and ^{13}C (B) NMR of compound **4f.**

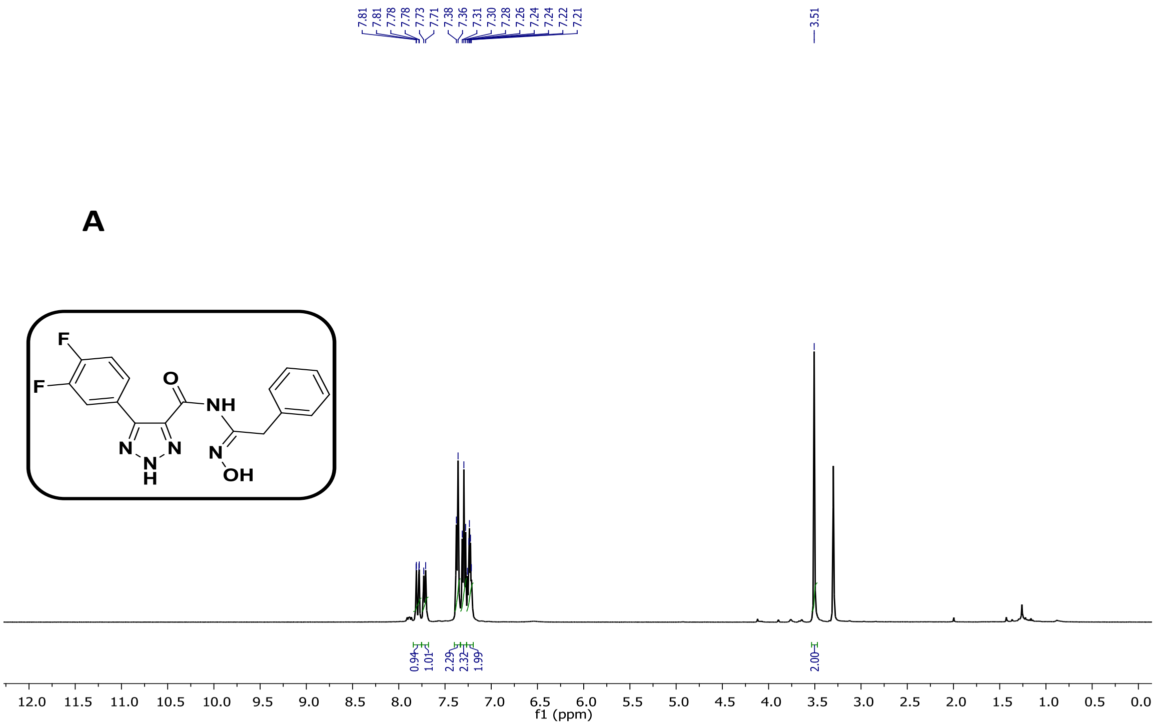

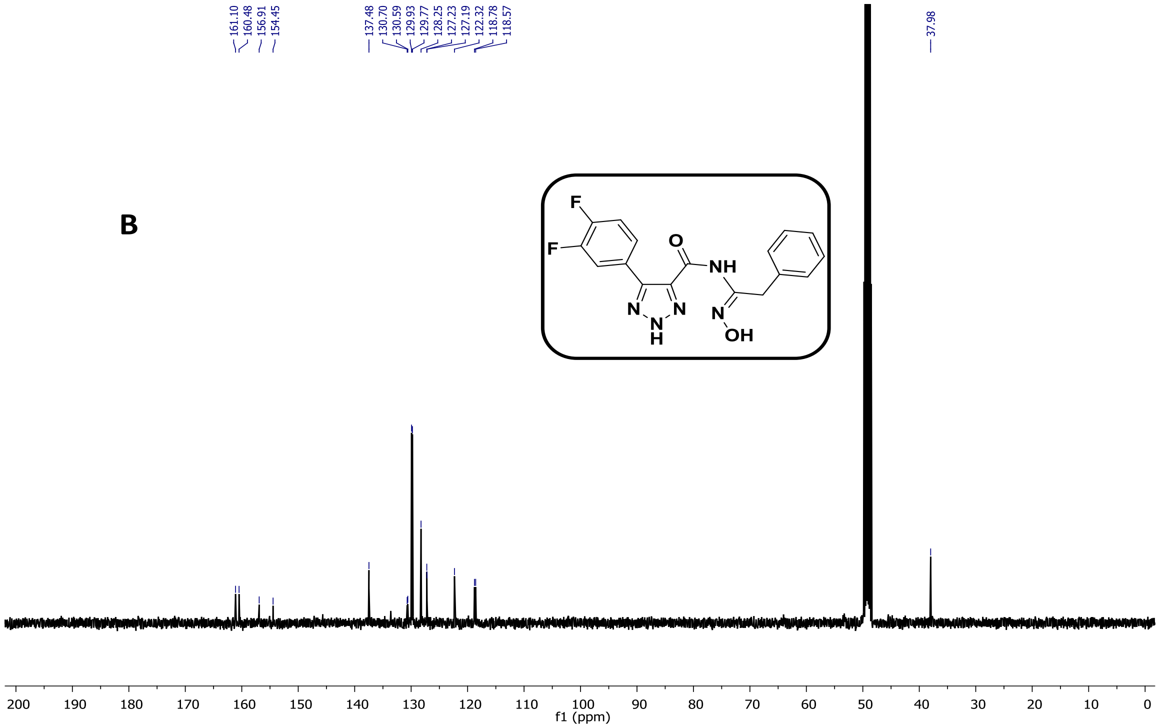

Figure S51. ^{1}H (A) and ^{13}C (B) NMR of compound **4g.**

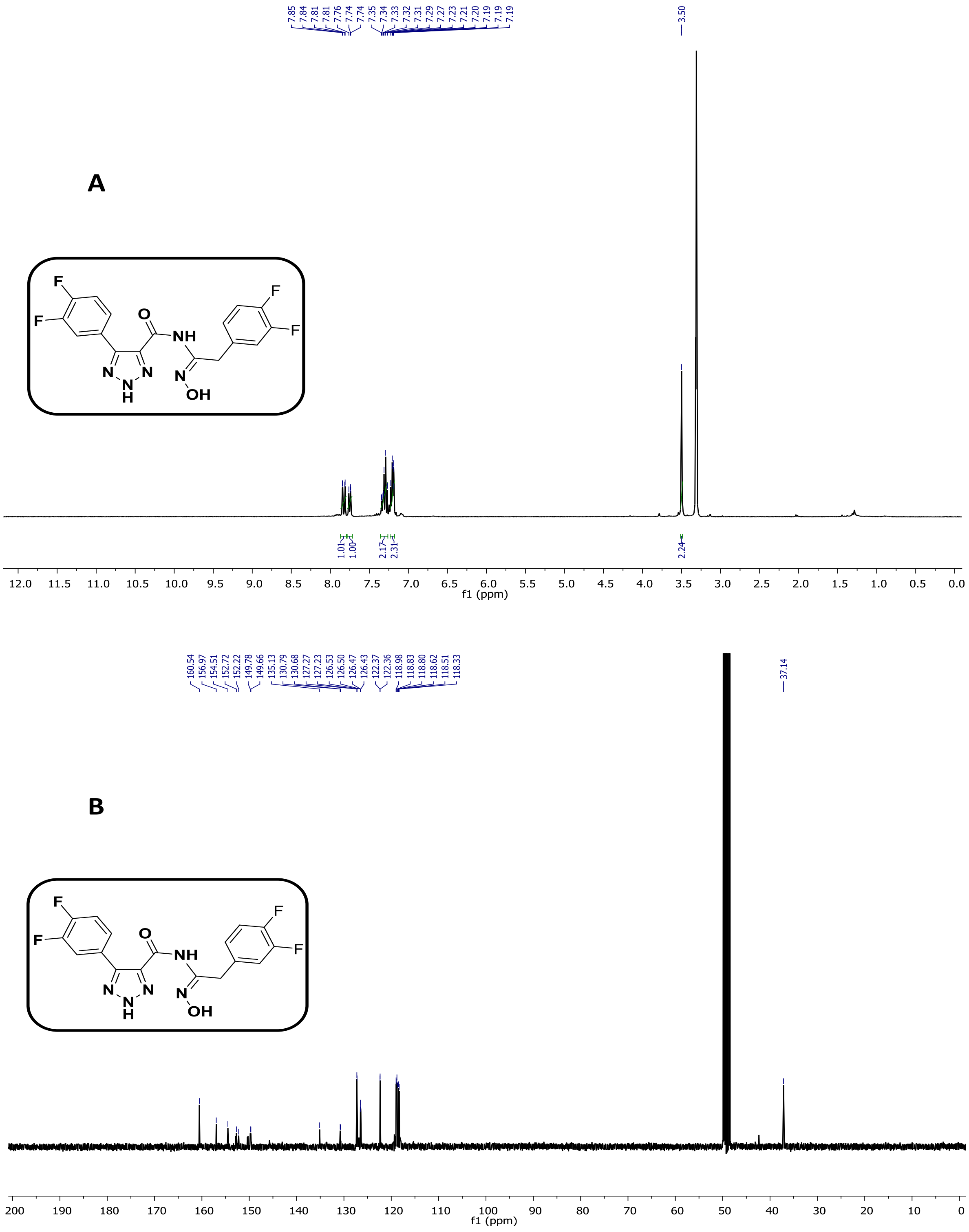

Figure S52. ^{1}H (A) and ^{13}C (B) NMR of compound **4h.**

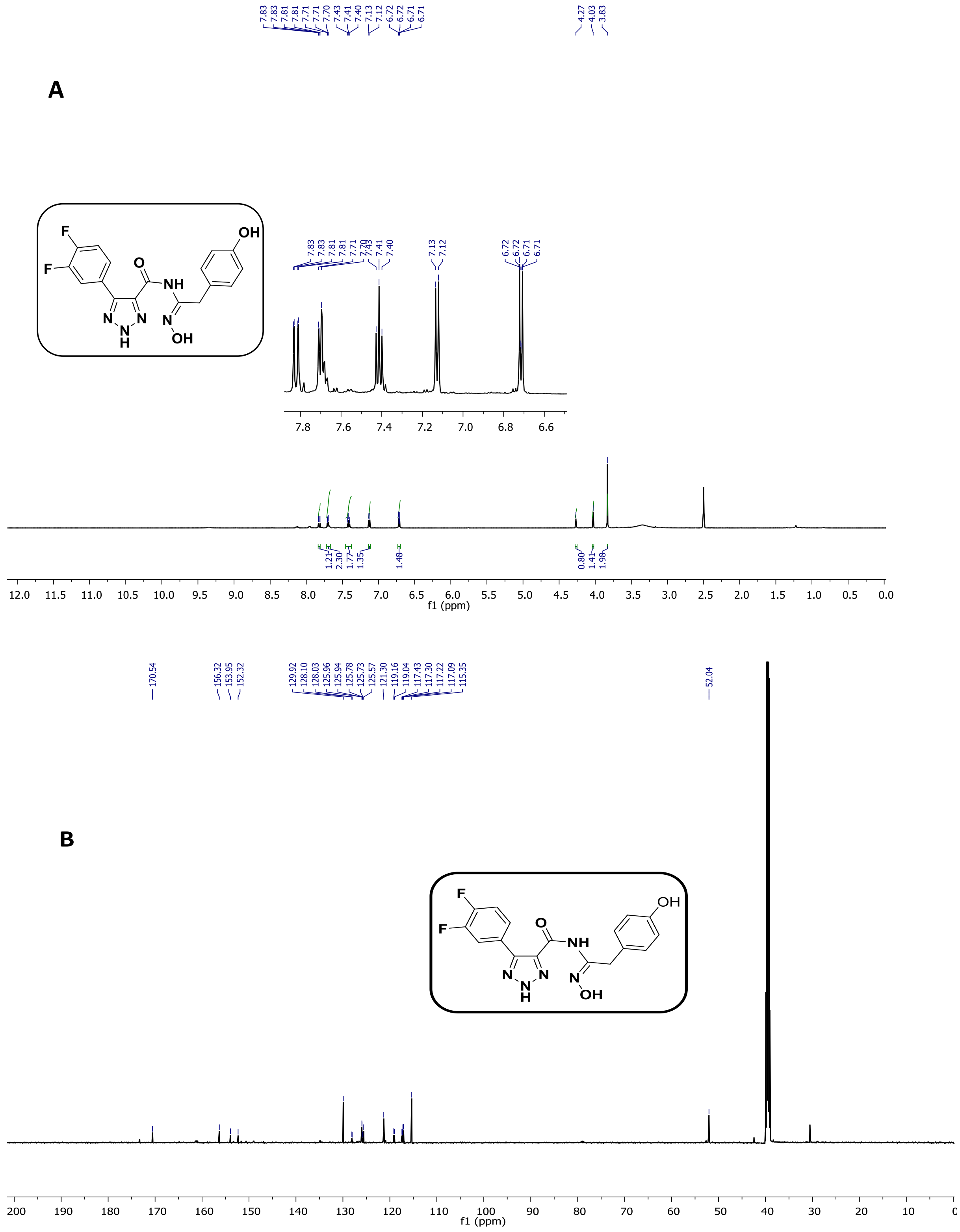

Figure S53. ^{1}H (A) and ^{13}C (B) NMR of compound **4i.**

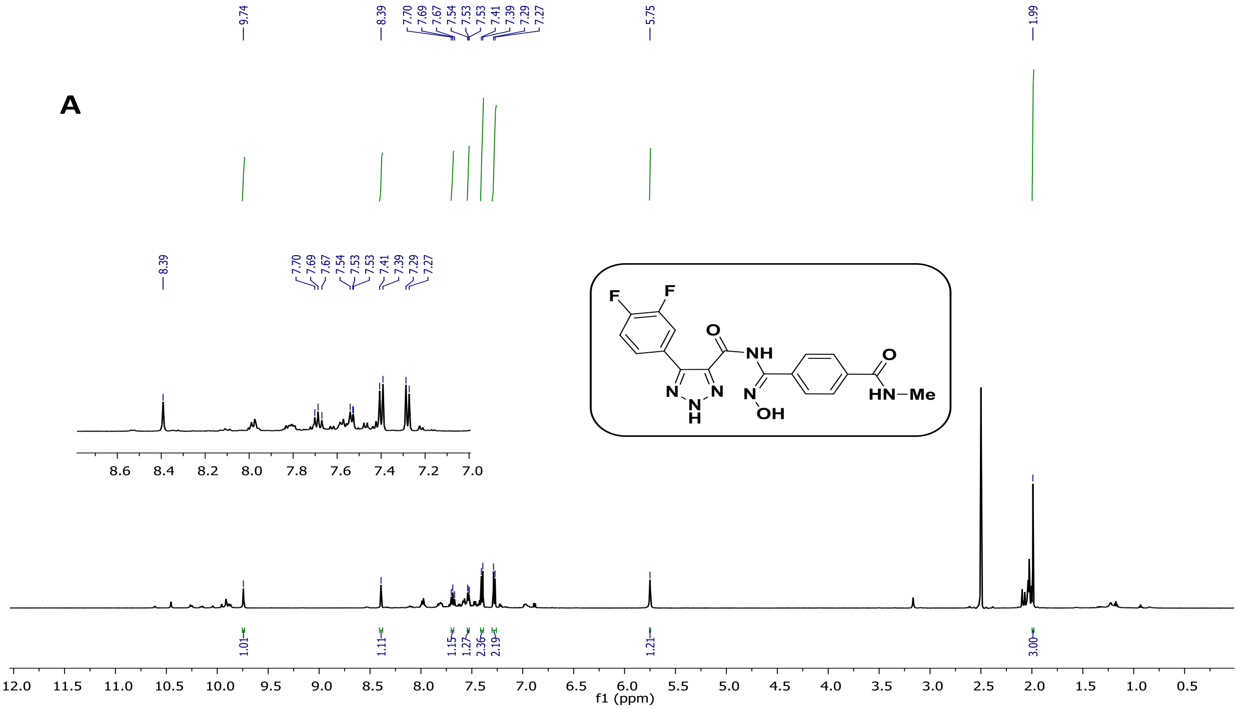

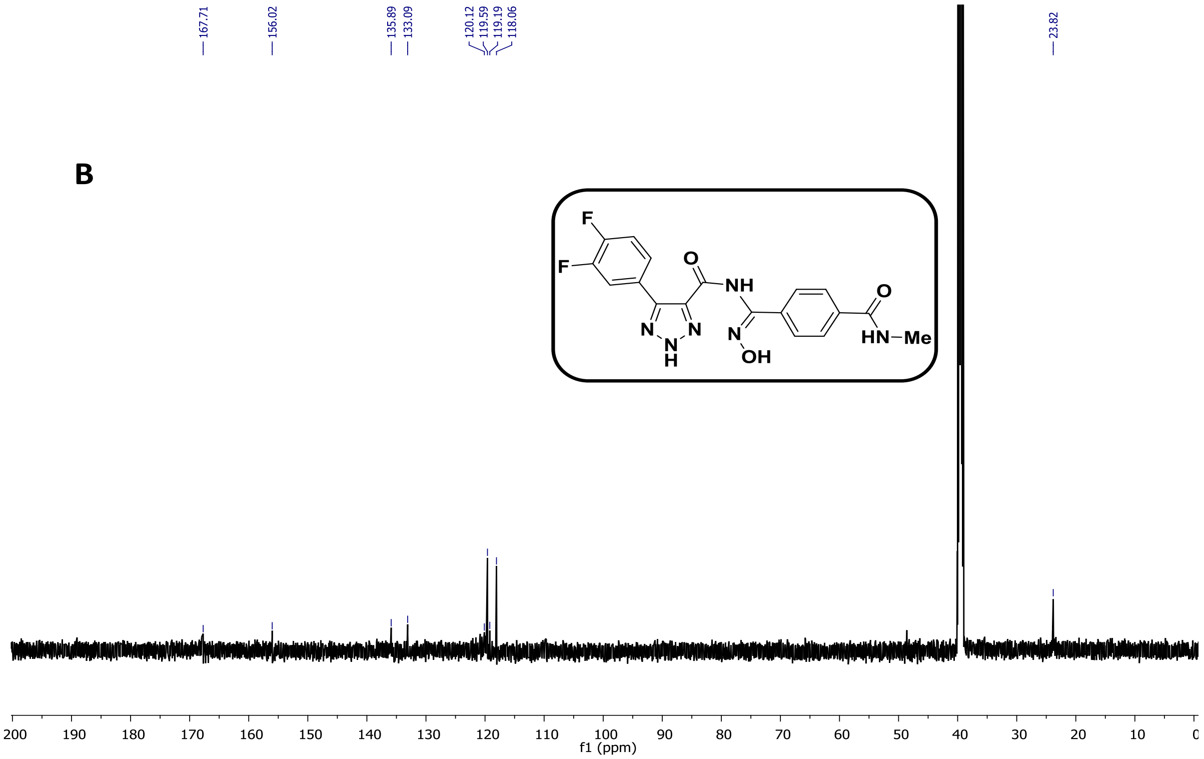

Figure S54. ^{1}H (A) and ^{13}C (B) NMR of compound **4j.**

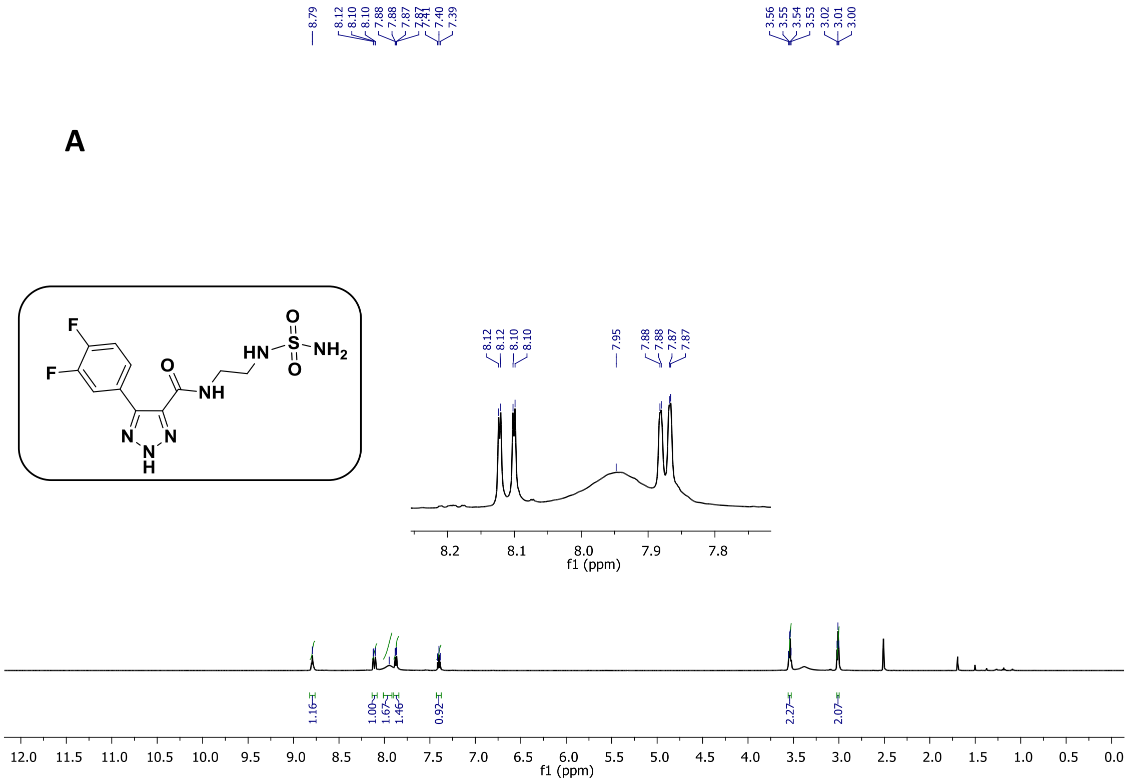

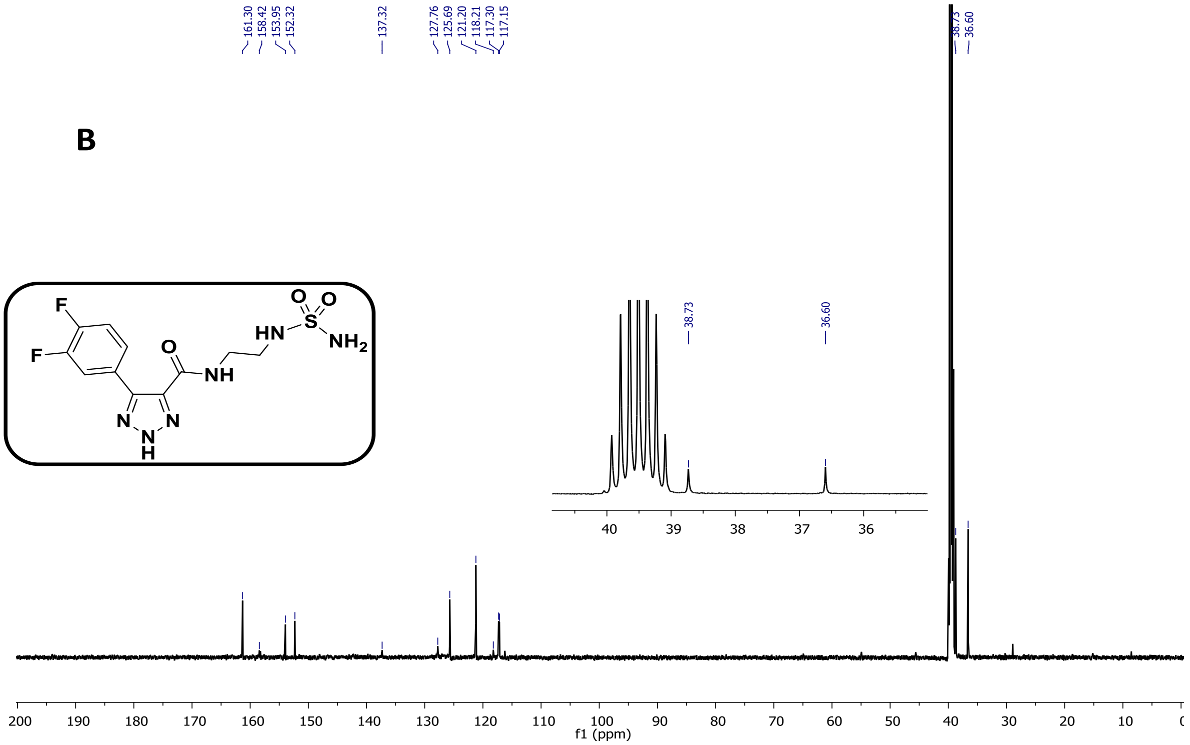

Figure S55. ^{1}H (A) and ^{13}C (B) NMR of compound **4k.**

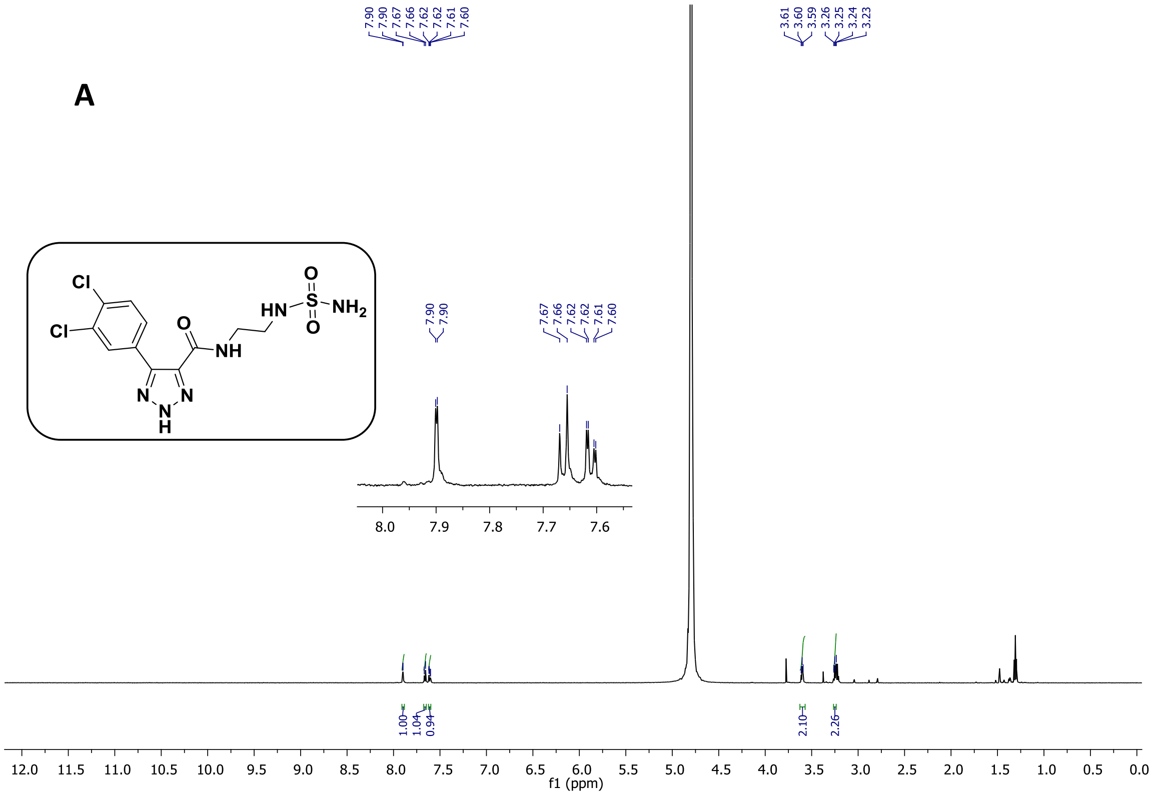

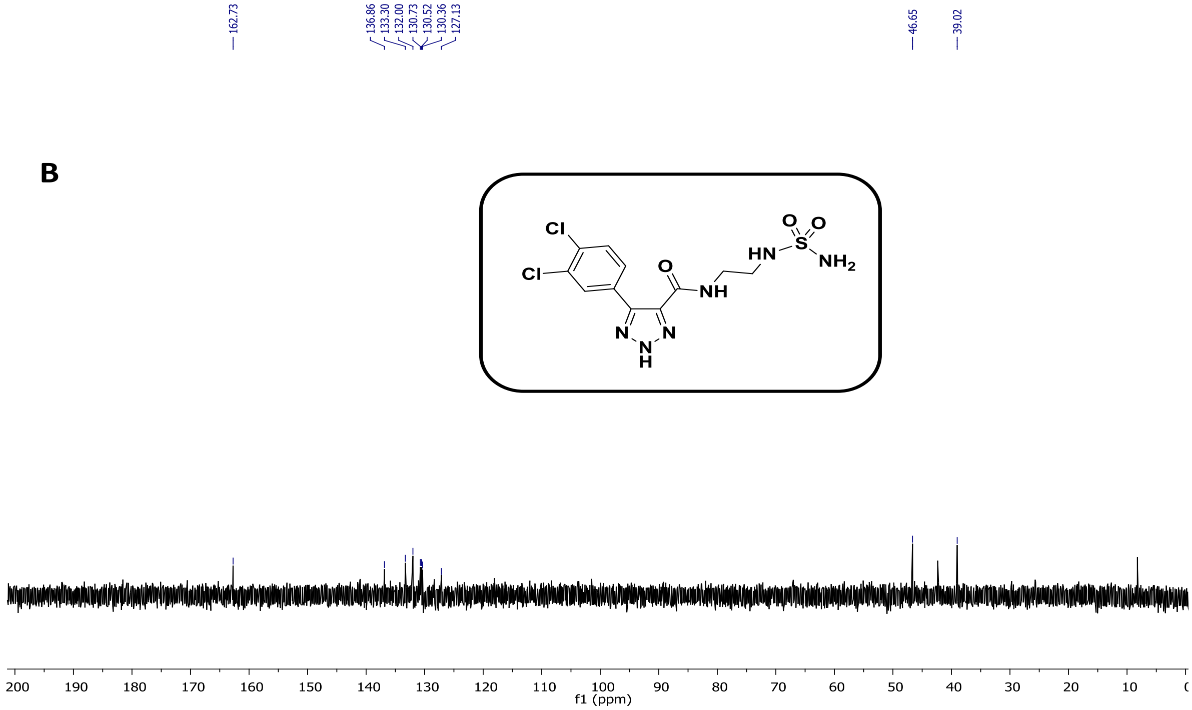

Figure S56. ^{1}H (A) and ^{13}C (B) NMR of compound **4l.**

References

1 Panda, S., Maity, P. & Manna, D. Transition Metal, Azide, and Oxidant-Free Homo- and Heterocoupling of Ambiphilic Tosylhydrazones to the Regioselective Triazoles and Pyrazoles. *Org. Lett.* **19**, 1534-1537 (2017).

2 Butler, R. N., Hanniffy, J. M., Stephens, J. C. & Burke, L. A. A ceric ammonium nitrate N-dearylation of N-p-anisylazoles applied to pyrazole, triazole, tetrazole, and pentazole rings: Release of parent azoles. Generation of unstable pentazole, HN(5)/N(5)(-), in solution. *J. Org. Chem.* **73**, 1354-1364 (2008).

3 Madadi, N. R. *et al.* Synthesis and biological evaluation of novel 4,5-disubstituted 2H-1,2,3-triazoles as cis-constrained analogues of combretastatin A-4. *Eur. J. Med. Chem.* **103**, 123-132 (2015).

4 Ponpandian, T. & Muthusubramanian, S. Tandem Knoevenagel-[3+2] cycloaddition-elimination reactions: one-pot synthesis of 4,5-disubstituted 1,2,3-(NH)-triazoles. *Tett. Lett.* **53**, 59-63 (2012).

5 Buckman, B. O., Nicholas, J. B., Emayan, K. & Seiwert, S. D. Lysophosphatidic acid receptor antagonists. *WO 2013025733 A1* (2013).

6 Glossop, P. A. & Lane, C. A. L. Novel compounds active as muscarinic receptor antagonists. *WO 2010007561 A1* (2010).

7 Yue, E. W. *et al.* INCB24360 (Epacadostat), a Highly Potent and Selective Indoleamine-2,3-dioxygenase 1 (ID01) Inhibitor for Immunooncology. *ACS Med. Chem. Lett.* **8**, 486-491 (2017).

8 Panda, S., Roy, A., Deka, S. J., Trivedi, V. & Manna, D. Fused Heterocyclic Compounds as Potent Indoleamine-2,3-dioxygenase 1 Inhibitors. *ACS Med. Chem. Lett.* **7**, 1167-1172 (2016).

9 Paul, S. *et al.* Synthesis and evaluation of oxindoles as promising inhibitors of the immunosuppressive enzyme indoleamine 2,3-dioxygenase 1. *Med. Chem. Comm.* **8**, 1640-1654 (2017).

10 Paul, S. *et al.* Nitrobenzofurazan derivatives of N '-hydroxyamidines as potent inhibitors of indoleamine-2,3-dioxygenase 1. *Eur. J. Med. Chem.* **121**, 364-375 (2016).

11 Pradhan, N. *et al.* Identification of Substituted 1H-Indazoles as Potent Inhibitors for Immunosuppressive Enzyme Indoleamine 2,3-Dioxygenase 1. *Chemistryselect* **2**, 5511-5517 (2017).
